# Triglyceride–Glucose–Waist‐to‐Weight Index: Novel Biomarker for Mild Cognitive Impairment in Older People With Sarcopenic Obesity

**DOI:** 10.1002/jcsm.70307

**Published:** 2026-05-11

**Authors:** Yue Xing, Qian Liu, Xiangfeng He, Lin Ma, Yanping Song, Nan Chen PhD

**Affiliations:** ^1^ Department of Rehabilitation Xinhua Hospital Affiliated to Shanghai Jiaotong University School of Medicine Shanghai China; ^2^ Department of Rehabilitation Chongming Hospital Affiliated to Shanghai University of Medicine and Health Sciences Shanghai China; ^3^ Shanghai University of Sport Shanghai China

**Keywords:** community‐dwelling, mild cognitive impairment, older people, sarcopenic obesity

## Abstract

**Background:**

Sarcopenic obesity (SO) is closely associated with mild cognitive impairment (MCI). However, traditional body composition‐based diagnostic criteria for SO fail to adequately incorporate insulin resistance, a key pathophysiological mechanism. This study aimed to investigate the association between SO, defined using multiple diagnostic criteria including novel insulin resistance indices, and MCI and to identify the optimal SO criterion for MCI identification.

**Methods:**

This study analysed data from the 2025 Chongming Older People Screening Project, CHARLS (2015**–**2020) and ELSA (2012**–**2017). We used multivariable logistic regression to assess the link between SO and MCI. The optimal obesity indicator for identifying MCI in sarcopenia was identified using ROC analysis and then incorporated into an XGBoost model to detect MCI risk. Subgroup analyses were performed to assess the association between SO and decline in multiple cognitive domains.

**Results:**

This study included 2326 individuals from the 2025 Chongming Older People Screening Project, 3392 from CHARLS and 1825 from ELSA. The mean age was 72.62 ± 5.56 years in the cross‐sectional study, 66.70 ± 5.39 years in CHARLS and 67.99 ± 5.55 years in ELSA. The proportion of female participants was 55.93% in the cross‐sectional study, 57.59% in ELSA and 40.60% in CHARLS. MCI was consistently identified across all assessment tools in three independent cohorts using the metabolically oriented definition integrating sarcopenia with an elevated triglyceride–glucose–waist‐to‐weight index (TyG‐WWI) (Chongming: OR = 3.04, 95% CI: 1.83–5.07, *p* < 0.001; CHARLS: HR = 1.57, 95% CI: 1.01–2.42, *p* = 0.043; and ELSA: HR = 1.87, 95% CI: 1.01–3.46, *p* = 0.047). TyG‐WWI was identified as the optimal obesity indicator to identify MCI (AUC = 0.71, 95% CI: 0.63–0.78). The machine learning model based on TyG‐WWI demonstrated strong discriminative performance in the cross‐sectional study (AUC = 0.93, 95% CI: 0.90–0.97) and maintained robust discriminative ability in external validation (CHARLS: AUC = 0.86, 95% CI: 0.80–0.92; ELSA: AUC = 0.84, 95% CI: 0.73–0.95). Subgroup analyses revealed that SO was associated with decline in multiple cognitive domains, particularly executive function and memory.

**Conclusions:**

This study demonstrates that a metabolically oriented SO definition integrating sarcopenia with elevated TyG‐WWI is a more effective tool for identifying MCI among community‐dwelling older people compared with traditional body composition‐based criteria.

## 1 Introduction

Mild cognitive impairment (MCI), a preclinical stage often preceding dementia, poses a significant challenge to healthy aging [1]. A meta‐analysis estimated its prevalence in China at approximately 25.3% among adults aged 55 years and older in community settings [2], with nearly half of these individuals progressing to dementia within 3 years [3]. Sarcopenic obesity (SO), a condition synergising low muscle mass with excessive adiposity that leads to more severe health outcomes than sarcopenia or obesity alone [4], has a global prevalence of approximately 11% in older people [5]. SO and MCI are already acknowledged as important factors contributing to disability among older people, and their impact is expected to become increasingly profound with the aging population.

The pathogenesis of SO arises from the convergence of age‐associated shifts in body composition and metabolic disturbances [6]. Metabolic disturbances, particularly insulin resistance, are a key pathophysiological mechanism in the development and progression of SO [7]. Insulin resistance, manifesting as diminished cellular insulin sensitivity, adversely affects muscle protein synthesis, leading to a progressive loss of muscle mass and ectopic lipid deposition [8, 9]. Moreover, insulin resistance contributes to cognitive impairment through the disruption of cerebral insulin signalling pathways, establishing a mechanistic link between metabolic dysfunction and cognitive decline [10]. Despite the established involvement of insulin resistance in SO, current diagnostic guidelines fail to incorporate insulin resistance biomarkers, primarily relying instead on body composition criteria [11, 12]. This omission represents a significant limitation, particularly in light of recent studies demonstrating that the triglyceride**–**glucose (TyG) index integrated with anthropometric measures provides a reliable surrogate for assessing insulin resistance and exhibits a strong association with SO [13, 14]. The inclusion of such biomarkers in SO definition could enhance the characterisation of its metabolic abnormality and improve its identification ability for associated cognitive outcomes, including MCI.

The prevalence of cognitive impairment among Chinese older people with SO ranges from 13.4% to 21.1%, establishing SO as an independent risk factor for cognitive impairment [15, 16]. Although several studies explored the relationship between SO and MCI, research that incorporates insulin resistance‐related indices into SO definition remains limited. Therefore, the present study aims to investigate the relationship between SO, defined using multiple diagnostic criteria, and MCI and to identify which specific SO definition exhibits the strongest association with MCI. The findings provide objective evidence to support early identification, intervention and prevention strategies for MCI.

## 2 Methods

### 2.1 Study Population

**FIGURE 1:**
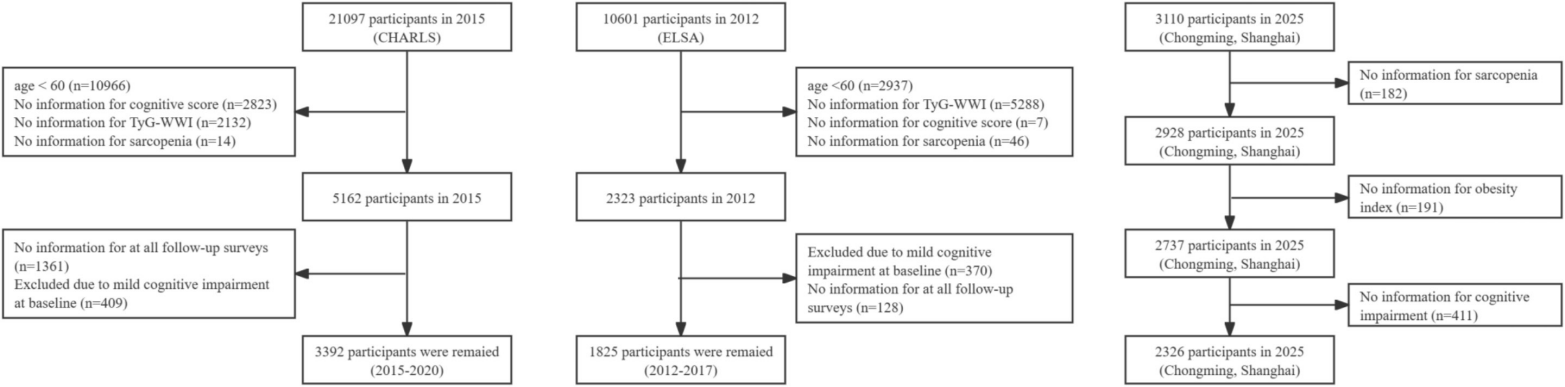
The flowchart of study. Abbreviations: CHARLS, China Health and Retirement Longitudinal Study; ELSA, English Longitudinal Study of Ageing; TyG‐WWI, triglyceride–glucose–waist‐to‐weight index.

This study incorporated data from a 2025 survey of older people in Chongming District, Shanghai, the China Health and Retirement Longitudinal Study (CHARLS) and the English Longitudinal Study of Aging (ELSA). Data from CHARLS waves 3**–**5 (2015**–**2020) and ELSA waves 6**–**8 (2012**–**2017) were utilised. A cross‐sectional exploratory analysis was initially conducted using data from the Shanghai Chongming study, with subsequent validation in independent cohorts from ELSA and CHARLS. The final analytical samples comprised 2326, 3392 and 1825 individuals from the survey, CHARLS and ELSA, respectively. Detailed inclusion and exclusion criteria are presented in Figure 1.

### 2.2 Sarcopenia

The diagnosis of sarcopenia was based on the 2019 criteria established by the Asian Working Group for Sarcopenia [17] and the European Working Group for Sarcopenia in Older People [18], which incorporates assessments of muscle mass, muscle strength, and physical performance. According to these criteria, sarcopenia is characterised by low muscle mass plus either low muscle strength or impaired physical performance. Individuals not meeting any of these conditions were categorised as the nonsarcopenia group as detailed in the Supporting Information.

### 2.3 Obesity

Obesity indicators were classified into two categories: body composition and metabolic abnormality indicators. Body composition indicators, specifically percentage body fat (PBF) and visceral fat area (VFA), were chosen in accordance with the European Society for Clinical Nutrition and Metabolism (ESPEN) and European Association for the Study of Obesity (EASO) Consensus Statement and Japanese guidelines [11, 12]. The selection of metabolic abnormality indicators, including TyG‐body mass index (TyG‐BMI), TyG‐waist circumference (TyG‐WC), TyG‐waist‐to‐hip ratio (TyG‐WHR), TyG‐waist‐to‐height ratio (TyG‐WHtR), TyG‐a body shape index (TyG‐ABSI), TyG‐body roundness index (TyG‐BRI) and TyG‐waist‐to‐weight index (TyG‐WWI), was based on relevant published literature [13, 19, 20]. The calculation formulas were as follows:

$$\mathrm{TyG}=\ln\left(\frac{\text{Triglycerides}\times \text{FastingGlucose}}{2}\right)$$

$$\mathrm{BMI}=\frac{\text{Weight}}{\text{Heigh}t^{2}}$$

$$\mathrm{WHR}=\frac{\text{Waist Circumference}}{\mathrm{Hip}\;\text{Circumference}}$$

$$\text{WHtR}=\frac{\text{Waist Circumference}}{\text{Height}}$$

$$\text{ABSI}=\frac{\mathrm{WC}}{\mathrm{BM}I^{\frac{2}{3}}\times \text{Heigh}t^{\frac{1}{2}}}$$

$$\mathrm{BRI}=364.2-365.5\times \sqrt{1-{\left(\frac{\mathrm{WC}}{2\pi }\right)}^{2}/{\left(0.5\text{Height}\right)}^{2}}$$

$$\mathrm{WWI}=\frac{\mathrm{WC}}{\sqrt{\text{Weight}}}$$

1. TyG‐BMI = TyG*BMI
2. TyG‐WC = TyG*WC
3. TyG‐WHR = TyG*WHR
4. TyG‐WHtR = TyG*WHtR
5. TyG‐ABSI = TyG*ABSI
6. TyG‐BRI = TyG*BRI
7. TyG‐WWI = TyG*WWI;

Cut‐off values for body composition indicators were determined in accordance with established guidelines [11, 12], and those for metabolic abnormality indicators were defined using the Youden index (Table S2). These criteria were applied to the ELSA and CHARLS cohorts. The diagnosis of SO requires the concurrent presence of both sarcopenia and obesity.

### 2.4 MCI

The 2025 Chongming, Shanghai study employed the Chinese versions of mini‐mental state examination (MMSE) and Montreal cognitive assessment (MoCA) to evaluate cognitive function. Both tests have a maximum total score of 30 points, with high scores indicating good cognitive performance. For participants with ≤ 12 years of education, one point was added to their MoCA total score if it was below 30. Education‐specific cut‐offs were applied to the MMSE and MoCA total scores to identify MCI. For the MMSE, the cut‐offs were as follows: ≤ 19 for illiterate individuals, ≤ 22 for those with primary education and ≤ 26 for participants with secondary education or above [21]. For the Chinese version of MoCA, the following education‐stratified cut‐offs were applied: illiterate (≤ 13), 1**–**6 years of education (≤ 19) and ≥ 7 years (≤ 24) [22].

MCI in the CHARLS and ELSA cohorts was evaluated through tests of orientation, memory and executive function detailed in the Supporting Information. The condition was defined using the concept of age‐associated cognitive decline (AACD) [23, 24]. Participants aged 60 years or older were divided into 5‐year age strata. Within each stratum, individuals performing less than one standard deviation below the group mean were classified as having cognitive impairment. Participants with MCI at baseline were excluded from the longitudinal analyses to ensure that only new‐onset MCI was considered during follow‐up.

### 2.5 Covariates and Others

Covariates included demographics (age, gender, education level, marital status and living status), lifestyle factors (smoking, drinking, physical activity and sleep duration), comorbidities (hypertension, diabetes, stroke, heart disease and liver disease) and lipid related blood indicators (HDL‐C, LDL‐C and TC) [25]. Details are available in the Supporting Information.

### 2.6 Statistical Analyses

All statistical analyses were performed using R software (version 4.4.1). Data were presented as mean ± standard deviation or as frequency (percentage). The normality of continuous variables was assessed using the Shapiro**–**Wilk test. Continuous variables conforming to a normal distribution were compared using one‐way ANOVA; nonnormally distributed variables were examined using Mann**–**Whitney U test or Kruskal**–**Wallis test. Categorical variables were compared using chi‐square test, with *p* < 0.05 defining statistical significance. Missing covariates were handled using multiple imputation. The missing rates for all covariates are reported in Table S1, and variables with a missing rate exceeding 50% were excluded from the analysis.

Multivariable logistic and Cox regression models were employed to examine the association between various SO definitions and MCI. Four models were constructed: Model 1 was unadjusted; Model 2 was adjusted for age, gender, education level, marital status and living status; Model 3 was additionally adjusted for smoking, drinking, physical activity and sleep duration; and Model 4 was adjusted similarly to Model 3 plus LDL‐C, HDL‐C, TC, hypertension, diabetes, stroke, liver disease and heart disease. Restricted cubic spline analysis was used to evaluate the dose**–**response relationship between obesity‐related indices and MCI in sarcopenia.

The discriminative ability of each obesity‐related indicator in identifying MCI within the sarcopenia population was evaluated using receiver operating characteristic (ROC) curves and area under the curve (AUC), with statistical comparisons performed using DeLong's test. Furthermore, a two‐stage modelling approach was employed. Firstly, least absolute shrinkage and selection operator (LASSO) regression was applied to screen for discriminative variables. Extreme gradient boosting (XGBoost) model, a tree‐based algorithm recognised for its robustness and high discriminative performance, was then employed to identify individuals with MCI. Internal validation was conducted using 10‐fold cross‐validation. Additionally, mediation analysis was performed using the R mediation package to explore potential mediators in the relationship between SO and MCI.

## 3 Results

### 3.1 Characteristics of Participants

The participant characteristics for the cross‐sectional and longitudinal cohorts are summarised in Tables S3 **–** S12. The mean age was comparable between the CHARLS (66.70 ± 5.39 years) and ELSA (67.99 ± 5.55 years) cohorts, whereas that in the cross‐sectional study was notably older (72.62 ± 5.56 years). The proportion of female participants was higher in the cross‐sectional study (55.93%) and ELSA (57.59%) than in CHARLS (40.60%). According to the MMSE and MoCA, the prevalence of MCI in the cross‐sectional cohort was 30.31% (*n* = 705) and 66.42% (*n* = 1545), respectively. During the 5‐year follow‐up, the incidence of new MCI cases was 582 (17.16%) in CHARLS and 227 (12.44%) in ELSA. Regardless of the diagnostic criteria applied, SO was consistently linked to an elevated prevalence of MCI (Tables S3 **–** 12).

### 3.2 Association of SO With MCI in the Cross‐Sectional Cohort

The results of multivariable logistic regression analyses assessing the associations between various definitions of obesity and SO with MCI (defined using the MMSE or MoCA) are shown in Table 1. The significant association between SO and MCI persisted across all definitions after full covariate adjustment. Specifically, the associations remained robust for S+TyG‐BRI (OR = 3.34, 95% CI: 1.86**–**6.02) and S+TyG‐WWI (OR = 3.04, 95% CI: 1.83**–**5.07) compared with those in the nonsarcopenia and nonobesity group. The associations between different SO definitions and MCI as defined by the MMSE and MoCA are presented in Tables S13 and S14, respectively. However, no significant associations were observed between any SO definition and MCI as defined by the MMSE.

**TABLE 1:** Logistic regression analysis of the association between SO and MCI defined by MOCA or MMSE.

Variables	Model 1		Model 2		Model 3		Model 4	
	OR (95% CI)	*P*	OR (95% CI)	*P*	OR (95% CI)	*P*	OR (95% CI)	*P*
N	Reference		Reference		Reference		Reference	
O	1.21 (0.95, 1.53)	0.125	1.15 (0.89, 1.48)	0.289	1.14 (0.88, 1.48)	0.315	1.19 (0.90, 1.56)	0.218
S	1.53 (0.88, 2.66)	0.130	1.20 (0.67, 2.14)	0.537	1.24 (0.69, 2.23)	0.465	1.21 (0.67, 2.19)	0.521
S+PBF/VFA	2.15 (1.43, 3.25)	**< 0.001**	1.77 (1.14, 2.73)	**0.010**	1.77 (1.14, 2.74)	**0.011**	1.81 (1.16, 2.82)	**0.009**
N	Reference		Reference		Reference		Reference	
O	1.11 (0.90, 1.37)	0.327	1.04 (0.83, 1.29)	0.744	1.02 (0.82, 1.28)	0.835	1.05 (0.81, 1.35)	0.725
S	1.40 (0.96, 2.03)	0.077	1.12 (0.76, 1.66)	0.570	1.14 (0.77, 1.69)	0.522	1.13 (0.76, 1.68)	0.554
S+TyG‐BMI	3.53 (1.84, 6.79)	**< 0.001**	2.91 (1.47, 5.76)	**0.002**	2.88 (1.45, 5.71)	**0.003**	2.89 (1.44, 5.80)	**0.003**
N	Reference		Reference		Reference		Reference	
O	1.14 (0.92, 1.42)	0.232	1.07 (0.85, 1.34)	0.572	1.05 (0.84, 1.33)	0.654	1.06 (0.82, 1.37)	0.670
S	1.31 (0.87, 1.98)	0.200	1.07 (0.70, 1.65)	0.751	1.10 (0.71, 1.70)	0.672	1.08 (0.70, 1.67)	0.733
S+TyG‐WC	2.87 (1.73, 4.78)	**< 0.001**	2.29 (1.34, 3.92)	**0.002**	2.26 (1.32, 3.87)	**0.003**	2.21 (1.28, 3.84)	**0.005**
N	Reference		Reference		Reference		Reference	
O	1.19 (0.97, 1.48)	0.099	1.15 (0.92, 1.44)	0.215	1.15 (0.92, 1.44)	0.231	1.19 (0.92, 1.53)	0.192
S	1.06 (0.68, 1.66)	0.801	0.86 (0.54, 1.37)	0.527	0.87 (0.54, 1.40)	0.570	0.86 (0.53, 1.38)	0.534
S+TyG‐WHR	3.04 (1.92, 4.82)	**< 0.001**	2.57 (1.58, 4.16)	**< 0.001**	2.60 (1.59, 4.22)	**< 0.001**	2.58 (1.57, 4.25)	**< 0.001**
N	Reference		Reference		Reference		Reference	
O	1.26 (1.03, 1.54)	**0.024**	1.07 (0.86, 1.32)	0.556	1.04 (0.84, 1.29)	0.735	1.03 (0.81, 1.32)	0.788
S	1.23 (0.82, 1.84)	0.309	0.96 (0.63, 1.46)	0.842	0.96 (0.63, 1.47)	0.852	0.94 (0.61, 1.44)	0.784
S+TyG‐WHtR	3.58 (2.13, 6.03)	**< 0.001**	2.67 (1.55, 4.61)	**< 0.001**	2.64 (1.52, 4.57)	**< 0.001**	2.57 (1.47, 4.48)	**< 0.001**
N	Reference		Reference		Reference		Reference	
O	1.20 (0.97, 1.49)	0.091	1.06 (0.84, 1.33)	0.619	1.03 (0.82, 1.30)	0.771	0.99 (0.77, 1.27)	0.961
S	0.85 (0.48, 1.51)	0.579	0.65 (0.36, 1.19)	0.163	0.69 (0.38, 1.25)	0.219	0.68 (0.37, 1.24)	0.210
S+TyG‐ABSI	2.50 (1.68, 3.71)	**< 0.001**	1.93 (1.27, 2.93)	**0.002**	1.90 (1.25, 2.90)	**0.003**	1.80 (1.18, 2.76)	**0.007**
N	Reference		Reference		Reference		Reference	
O	1.48 (1.22, 1.80)	**< 0.001**	1.23 (1.00, 1.51)	0.055	1.20 (0.97, 1.48)	0.091	1.24 (0.99, 1.56)	0.065
S	1.28 (0.87, 1.89)	0.214	1.01 (0.67, 1.52)	0.963	1.02 (0.67, 1.54)	0.938	1.00 (0.66, 1.52)	0.999
S+TyG‐BRI	4.71 (2.69, 8.23)	**< 0.001**	3.40 (1.90, 6.08)	**< 0.001**	3.36 (1.87, 6.01)	**< 0.001**	3.34 (1.86, 6.02)	**< 0.001**
N	Reference		Reference		Reference		Reference	
O	1.49 (1.23, 1.80)	**< 0.001**	1.27 (1.04, 1.56)	**0.022**	1.23 (1.01, 1.52)	**0.049**	1.25 (1.00, 1.58)	0.055
S	1.03 (0.67, 1.57)	0.894	0.82 (0.52, 1.27)	0.373	0.82 (0.52, 1.29)	0.387	0.79 (0.50, 1.25)	0.319
S+TyG‐WWI	4.10 (2.54, 6.63)	**< 0.001**	3.14 (1.90, 5.19)	**< 0.001**	3.10 (1.87, 5.13)	**< 0.001**	3.04 (1.83, 5.07)	**< 0.001**

*Note:* Model 1 was a crude model; Model 2 was adjusted for age, gender, education, marital and living status; Model 3 was adjusted as Model 2 plus lifestyle factors (smoking, alcohol consumption, physical activity and sleep duration). Model 4 was adjusted as Model 3 plus LDL‐C, HDL‐C, TC, hypertension, diabetes, stroke, liver disease and heart disease. Bold emphasis indicates statistical significance (*p* < 0.05).

Abbreviations: CI, confidence interval; N, without sarcopenia and obesity; O, only obesity; OR, odd ratio; S, only sarcopenia; S+PBF/VFA, sarcopenia and high body fat percentage or visceral fat area; S+TyG‐ABSI, sarcopenia and high triglyceride–glucose‐a body shape index; S+TyG‐BMI, sarcopenia and high triglyceride–glucose‐body mass index; S+TyG‐BRI: triglyceride–glucose‐body roundness index; S+TyG‐WC, sarcopenia and high triglyceride–glucose‐waist circumference; S+TyG‐WHR, sarcopenia and high triglyceride–glucose–waist‐to‐hip ratio; S+TyG‐WHtR, sarcopenia and high triglyceride–glucose–waist‐to‐height ratio; S+TyG‐WWI, sarcopenia and high triglyceride–glucose–waist‐to‐weight index.

### 3.3 Relationship Between Obesity Indices and MCI in Sarcopenia

**FIGURE 2:**
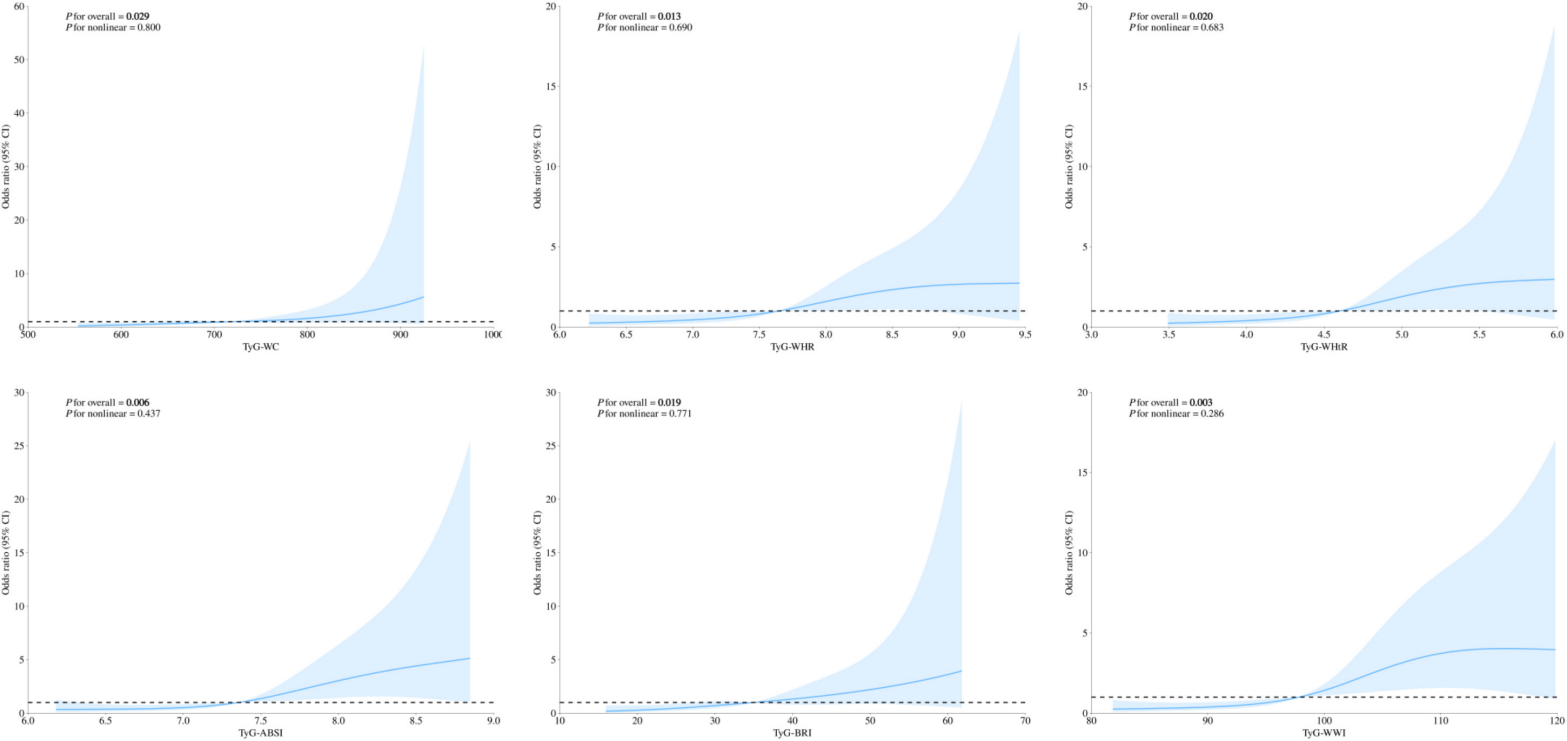
RCS of association between obesity indices and MCI defined by MOCA or MMSE in sarcopenia. Abbreviation: OR, odd ration; TyG‐WC, triglyceride–glucose‐waist circumference; TyG‐WHR, triglyceride–glucose–waist‐to‐hip ratio; TyG‐WHtR, triglyceride–glucose–waist‐to‐height ratio; TyG‐ABSI, triglyceride–glucose‐a body shape index; TyG‐BRI: triglyceride–glucose‐body roundness index; TyG‐WWI, triglyceride–glucose–waist‐to‐weight index.

As shown in Figure 2, a dose**–**response relationship was observed between the increasing values of obesity indices and MCI risk (defined using the MMSE or MoCA). Specifically, high TyG‐WWI levels were significantly associated with an increased risk of MCI (*P* for overall = 0.003). However, this pattern was absent for the indices analysed in Figure S1, which showed no significant trends (PBF: *P* for overall = 0.260; VFA: *P* for overall = 0.401).

### 3.4 Ability of Obesity Indicators to Identify MCI in Sarcopenia

Table S15 presents the identification ability of various obesity indices for MCI as defined by the MMSE. All indicators demonstrated limited discriminative efficacy, and no statistically significant differences were observed compared with VFA. Tables S16 **–** S20 summarise the identification ability of various obesity indices for MoCA‐defined MCI among individuals with sarcopenia, with analyses stratified by age and gender. TyG‐BRI and TyG‐WWI demonstrated moderate screening efficacy for MCI, with AUC values of 0.67 and 0.66 in the total population, respectively; 0.71 and 0.73 in females, respectively; 0.61 and 0.56 in males, respectively; 0.63 and 0.64 in the 60**–**75 age group, respectively; and 0.69 and 0.67 in those over 75 years old, respectively. Their performance was statistically superior to that of body composition indicators in the total population and among female participants.

**FIGURE 3:**
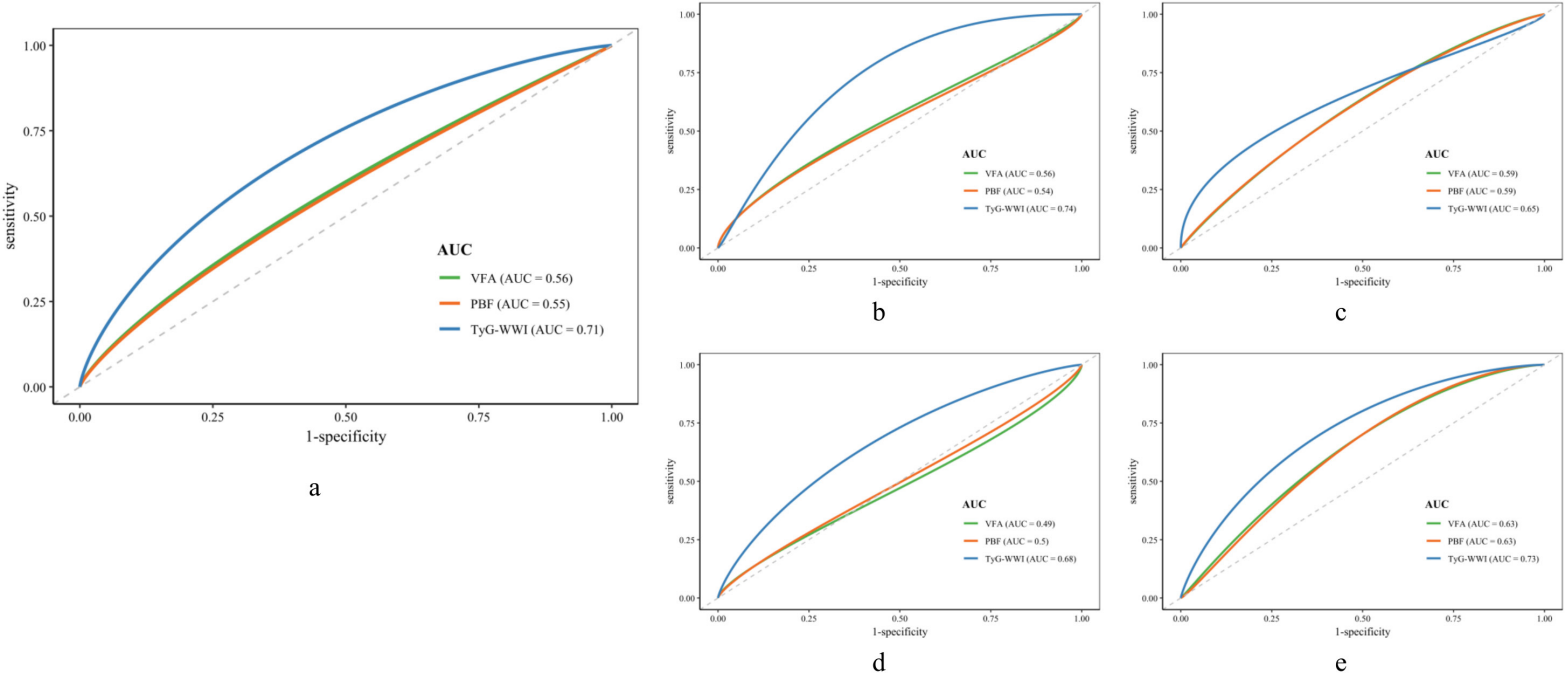
The ability of obesity indicators to identify MCI (defined by MMSE or MoCA) in sarcopenia. *Note:* a, total participants; b, female participants; c, male participants; d, 60–75 participants; e, above 75 participants. Abbreviation: PBF, body fat percentage; VFA, visceral fat area; TyG‐WWI, triglyceride–glucose–waist‐to‐weight index.

**FIGURE 4:**
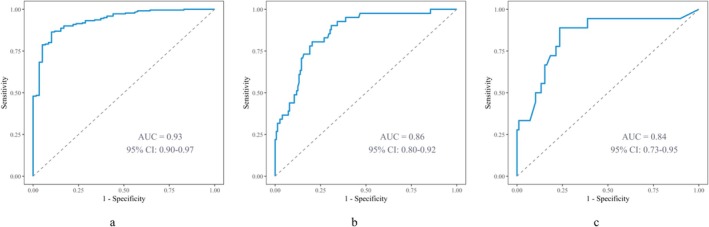
The ability of TyG‐WWI models to predict MCI in sarcopenia. *Note:* a, in 2025 Chongming, Shanghai; b, in CHARLS; c, in ELSA.

Figure 3 and Tables S21 **–** S25 present the identification ability of various obesity indicators for MCI (defined by the MMSE or MoCA) in individuals with sarcopenia and compared with body composition indices. TyG‐WWI emerged as the strongest predictor, showing AUC values of 0.71 (total population), 0.74 (females), 0.65 (males), 0.68 (60**–**75 years) and 0.73 (above 75 years). Its performance was statistically superior to that of body composition‐based (PBF or VFA) screening for MCI in the total population, among females and in participants aged 60 years and above (*p* < 0.05) (Figure 4).

### 3.5 Association of SO With New‐Onset MCI in Longitudinal Cohort

In CHARLS (Table 2), the participants with obesity alone did not show a significantly increased risk of new‐onset MCI across any of the models compared with the nonsarcopenia and nonobesity participants (all *p* > 0.05). By contrast, sarcopenia alone was associated with a significantly high risk of new‐onset MCI in the crude model (HR = 1.74, 95% CI: 1.25**–**2.43), and this association remained significant after partial adjustment (Models 2 and 3) but was attenuated to nonsignificant in the fully adjusted model (HR = 1.40, 95% CI: 0.99**–**1.98). The combination of sarcopenia and obesity defined by TyG‐WWI (S+TyG‐WWI) was consistently associated with an increased risk of new‐onset MCI across all models, with the strongest effect observed in the crude model (HR = 1.97, 95% CI: 1.30**–**2.98). This association remained significant even after full adjustment (HR = 1.57, 95% CI: 1.01**–**2.42).

**TABLE 2:** Cox regression analysis of the association between SO and new‐onset MCI in CHARLS.

Variables	Model 1		Model 2		Model 3		Model 4	
	HR (95% CI)	*P*	HR (95% CI)	*P*	HR (95% CI)	*P*	HR (95% CI)	*P*
N	Reference		Reference		Reference		Reference	
O	1.09 (0.92, 1.31)	0.318	1.00 (0.83, 1.21)	0.992	1.01 (0.84, 1.22)	0.915	1.09 (0.88, 1.34)	0.443
S	1.74 (1.25, 2.43)	**0.001**	1.54 (1.10, 2.16)	**0.013**	1.48 (1.05, 2.08)	**0.024**	1.40 (0.99, 1.98)	0.054

S+TyG‐WWI	1.97 (1.30, 2.98)	**0.002**	1.57 (1.02, 2.41)	**0.041**	1.51 (0.98, 2.32)	0.063	1.57 (1.01, 2.42)	**0.043**

*Note:* Model 1 was a crude model; Model 2 was adjusted for age, gender, education, marital status; Model 3 was adjusted as Model 2 plus lifestyle factors (smoking, alcohol consumption, and sleep duration). Model 4 was adjusted as Model 3 plus LDL‐C, HDL‐C, TC, hypertension, diabetes, stroke, liver disease and heart disease. Bold emphasis indicates statistical significance (*p* < 0.05).

Abbreviations: CI, confidence interval; HR, Hazard ration; N, without sarcopenia and obesity; O, only obesity; S, only sarcopenia; S+TyG‐WWI, sarcopenia and high triglyceride–glucose–waist‐to‐weight index.

In ELSA (Table 3), the participants with obesity alone exhibited a significantly higher risk of new‐onset of MCI in the crude model (HR = 1.52, 95% CI: 1.16**–**2.00) and after partial adjustment (HR = 1.35, 95% CI: 1.02**–**1.78) compared with the nonsarcopenia and nonobesity participants. However, this association was attenuated to nonsignificant after further adjustment in Models 3 and 4 (both *p* > 0.05). By contrast, sarcopenia alone was not associated with new‐onset MCI risk in any of the models (all *p* > 0.05). The combination of sarcopenia and obesity defined by TyG‐WWI (S+TyG‐WWI) was consistently and significantly associated with an increased risk of new‐onset MCI across all models. This association was strongest in the crude model (HR = 2.51, 95% CI: 1.43**–**4.40) and remained significant even after full adjustment (HR = 1.87, 95% CI: 1.01**–**3.46).

**TABLE 3:** Cox regression analysis of the association between SO and new‐onset MCI in ELSA.

Variables	Model 1		Model 2		Model 3		Model 4	
	HR (95% CI)	*P*	HR (95% CI)	*P*	HR (95% CI)	*P*	HR (95% CI)	*P*
N	Reference		Reference		Reference		Reference	
O	1.52 (1.16, 2.00)	**0.003**	1.35 (1.02, 1.78)	**0.035**	1.28 (0.96, 1.70)	0.092	1.32 (0.94, 1.83)	0.106
S	1.16 (0.56, 2.38)	0.695	1.20 (0.58, 2.50)	0.624	1.11 (0.53, 2.33)	0.777	1.03 (0.49, 2.15)	0.943
S+TyG‐WWI	2.51 (1.43, 4.40)	**0.001**	2.14 (1.21, 3.81)	**0.010**	1.95 (1.08, 3.51)	**0.027**	1.87 (1.01, 3.46)	**0.047**

*Note:* Model 1 was a crude model; Model 2 was adjusted for age, gender, education, marital status; Model 3 was adjusted as Model 2 plus lifestyle factors (smoking, alcohol consumption, physical activity and sleep duration). Model 4 was adjusted as Model 3 plus LDL‐C, HDL‐C, TC, hypertension, diabetes, stroke and heart disease. Bold emphasis indicates statistical significance (*p* < 0.05).

Abbreviations: CI, confidence interval; HR, Hazard ratio; N, without sarcopenia and obesity; O, only obesity; S, only sarcopenia; S+TyG‐WWI, sarcopenia and high triglyceride–glucose–waist‐to‐weight index.

### 3.6 Model Development and Validation

Within the sarcopenic population, we applied the XGBoost algorithm incorporating the TyG‐WWI index to identify individuals with MCI. LASSO regression was employed to select candidate variables for model construction. The LASSO regression coefficient path plot and cross‐validation results are presented in Figures S2 and S3, respectively. SHAP analysis was conducted to interpret the model's output, with the results shown in Figure S4. The model developed in the Chongming cohort demonstrated excellent discriminatory performance with an AUC of 0.93 (95% CI: 0.90**–**0.97). External validation in the CHARLS and ELSA cohorts further confirmed the model's generalisability with AUC values of 0.86 (95% CI: 0.80**–**0.92) and 0.84 (95% CI: 0.73**–**0.95), respectively, indicating its robust discriminative performance across different populations.

### 3.7 Subgroup Analysis

Although no significant interactions were detected across most subgroups, the association between SO and MCI appeared to be strong in certain populations (Table S26). Specifically, the odds ratios were notably high among individuals with low education level (below high school: OR = 3.74, 95% CI: 1.95**–**7.17), those who never smoked (OR = 4.19, 95% CI: 2.16**–**8.14) or never drank (OR = 3.88, 95% CI: 2.04**–**7.39) and those with long sleep duration (> 8 h: OR = 14.02, 95% CI: 1.59**–**123.87). This finding suggests that these subgroups may be highly vulnerable to the combined effect of sarcopenia and obesity defined by TyG‐WWI on MCI risk.

The association between S+TyG‐WWI and cognitive function was consistently observed across the three independent cohorts. In the Chongming cohort (Table S27), S+TyG‐WWI was significantly associated with poor performance across multiple cognitive domains including visuoconstructional skills (β = **−**0.56, 95% CI: from **−**0.76 to **−**0.36), executive function (β = **−**0.28, 95% CI: from **−**0.54 to **−**0.02), naming (β = **−**0.32, 95% CI: from **−**0.51 to **−**0.13) and recall (β = **−**0.26, 95% CI: from **−**0.51 to **−**0.01) after full covariate adjustment. Similarly, in the CHARLS cohort (Table S28), S+TyG‐WWI was significantly associated with low scores in memory (β = **−**0.69, 95% CI: from **−**1.31 to **−**0.08) and drawing ability (β = **−**0.18, 95% CI: from **−**0.27 to **−**0.09). In the ELSA cohort (Table S29), S+TyG‐WWI was also significantly associated with poor memory performance (β = **−**0.98, 95% CI: from **−**1.59 to **−**0.37).

### 3.8 Mediated Effect

Inflammatory factors were tested as mediators of the association between SO and cognitive function (Table S30). However, no significant mediation effects were observed.

## 4 Discussion

To the best of our knowledge, this study is the first to investigate the association between SO defined using multiple diagnostic criteria, including traditional body composition (PBF and VFA) and novel insulin resistance indices, and MCI among community‐dwelling older people aged 60 years and above in China. The SO definition incorporating sarcopenia and a high TyG‐WWI served as a more effective discriminative indicator for MCI compared with the definitions based on sarcopenia and high PBF or VFA, outperforming the other criteria used in this study.

This finding is significant in the state of an unstandardised SO definition. Current frameworks, such as those from ESPEN and EASO or the Japanese Working Group, primarily define SO on the basis of alterations in body composition [11, 12]. However, this phenotype‐focused approach fails to capture a key pathophysiological mechanism: insulin resistance, which is initiated by adipose tissue accumulation and ectopic lipid deposition [9]. This metabolic dysfunction is further exacerbated by the chronic inflammatory and metabolically abnormal state of sarcopenia, creating a vicious cycle where muscle loss impairs glucose disposal, worsens insulin resistance and mutually aggravates both conditions, ultimately leading to severe health outcomes in older people [26]. Our study incorporated an insulin resistance‐related indicator (TyG‐WWI) into the SO definition. We demonstrated that SO (s+TyG‐WWI) was cross‐sectionally and longitudinally associated with new‐onset MCI in the CHARLS and ELSA cohorts, indicating the stability of this indicator across different populations and study designs. Only SO (s+TyG‐WWI) showed a significant association with MCI; sarcopenia alone or obesity alone was not significantly associated with MCI after full covariate adjustment. These findings are consistent with a recent longitudinal study with a 6‐year follow‐up of 1097 participants by Vázquez‐Lorente et al. [27], who reported that neither sarcopenia alone nor obesity alone was significantly associated with cognitive outcomes relative to the nonsarcopenia or nonobesity participants. However, a study in Tokyo reported that sarcopenia was associated with a 3.21 times higher risk of dementia, and SO was associated with a 2.11 times higher risk of MCI and a 6.17 times higher risk of dementia [4]. This discrepancy may be partly attributed to the lack of uniformity in SO definitions across studies. The Tokyo study used BMI to define obesity, a metric that may not adequately capture obesity‐related metabolic risks such as insulin resistance, which has been shown to significantly influence cognitive decline [28]. In summary, these findings underscore the importance of integrating insulin resistance assessment into SO evaluation. A definition that captures body composition and metabolic dysfunction may elucidate the underlying mechanisms linking SO to cognitive impairment and improve risk stratification in older populations.

TyG index integrated with anthropometric measures provides a reliable surrogate for assessing insulin resistance and exhibits a strong association with SO [13, 14]. However, the existing literature on the relationship between TyG‐based indices and cognitive outcomes remains inconsistent [29, 30, 31]. For example, Ze‐Kun Wei et al. [30] reported that the combination of elevated TyG levels and obesity indices was independently associated with an increased risk of MCI, particularly among older people (≥ 60 years). By contrast, Zhang et al. [31] observed a negative correlation between TyG‐BMI and MMSE, suggesting a possible protective effect. These discrepancies highlight the need for a systematic evaluation of TyG‐based indices. Our study addresses this gap by systematically evaluating the association between multiple TyG‐based indices and MCI in a sarcopenic population. A key finding is that TyG‐WWI emerged as the optimal obesity‐related indicator in the cross‐sectional analyses. It demonstrated superior performance in identifying MCI (defined by either the MMSE or MoCA) with an AUC of 0.71, outperforming PBF (AUC = 0.55) and VFA (AUC = 0.56) alone. The screening capability of TyG‐WWI was significantly stronger in females than in males. This observed gender disparity may be related to postmenopausal declines in ovarian hormones. Loss of oestrogen postmenopause, independent of the aging process, increases total adipose tissue mass and reduces lean mass, thereby intensifying the central obesity burden in women [32]. Previous studies suggested that SO is part of an obesity paradox; however, our findings did not corroborate this relationship. The positive association between this indicator and MCI persisted even among adults aged 75 years and above, a finding consistent with prior evidence from China [33]. Moreover, the TyG‐WWI‐based model demonstrated robust discriminatory ability for MCI in the Chongming cohort (AUC = 0.93), with excellent generalisability confirmed through external validation in CHARLS (AUC = 0.86) and ELSA (AUC = 0.84). We attribute the superior performance of TyG‐WWI to its ability to comprehensively capture the dual pathophysiological hazards of insulin resistance and central obesity. Central obesity, a key driver of insulin resistance through mechanisms such as oxidative stress, is more strongly associated with cerebral structural abnormalities and MCI than general obesity [34]. By integrating waist circumference and body weight, WWI is a robust indicator of central obesity that has been independently linked to cognitive decline [35]. Consequently, TyG‐WWI provides a more comprehensive assessment of the metabolic abnormality posed by obesity to cognitive function than PBF or VFA alone, explaining its strength as a biomarker in our sarcopenic population.

Subgroup analyses revealed that SO exerted a broad impact on cognitive function, affecting memory and nonmemory domains. The association between SO and MCI was consistently observed across the three independent cohorts (Chongming, CHARLS and ELSA), with memory and executive‐related functions emerging as the most vulnerable domains. These findings align with the core diagnostic criteria for MCI, which emphasise the importance of assessing amnestic and nonamnestic domains. The particularly strong and consistent association with memory function across all three cohorts suggests that SO may preferentially target the hippocampus, which is critical for memory consolidation and known to be susceptible to metabolic and vascular insults [36]. Similarly, the observed deficits in executive function—a domain dependent on frontal‐subcortical integrity—point to potential pathways involving insulin resistance or chronic inflammation, all of which are characteristic of SO [37]. Despite the consistent pattern of associations, differences in cognitive assessment tools across cohorts may have influenced the sensitivity of domain‐specific detection. The Chongming cohort employed the MoCA, which has great sensitivity for detecting executive dysfunction and visuoconstructional impairments, potentially explaining the broad range of significant domains observed in this cohort. By contrast, CHARLS and ELSA used AACD criteria. Although these criteria are validated and widely applied, they may be less sensitive to subtle deficits in nonmemory domains. This tool‐related heterogeneity may partly account for the variations in the number and type of cognitive domains identified across the cohorts. Despite these methodological differences, the convergence of findings on memory and executive function reinforces the robustness of the association between SO and these core cognitive domains. These domain‐specific findings have important clinical implications. The consistent involvement of memory and executive function suggests that cognitive screening in individuals with SO should encompass a broad range of domains rather than focusing solely on memory. Furthermore, the vulnerability of executive function, a key determinant of functional independence in older people, underscores the need for early intervention in this population to prevent or delay disability.

We conducted a mediation analysis to further investigate the pathophysiological process linking the metabolic dysfunction of SO to the observed cognitive decline. However, no significant mediation effects were detected for these markers. This finding suggests that the specific inflammatory indicators measured in our study may not serve as primary mediators in the relationship between SO and MCI. However, we cannot exclude the possibility that other inflammatory markers not assessed in this study, such as interleukin‐6 (IL‐6) or tumour necrosis factor‐α, may play a mediating role. Future studies incorporating a broad panel of inflammatory cytokines are warranted to further elucidate the potential involvement of inflammation in the SO‐MCI pathway. Other mechanisms, including oxidative stress, mitochondrial dysfunction or dysregulation of neurotrophic factors, may also contribute and deserve investigation.

This study was strengthened by its comprehensive methodology which involved applying multiple diagnostic criteria for SO. This design enabled a significant finding: the novel TyG‐WWI criterion was more significantly associated with MCI in sarcopenia compared with traditional body composition definitions, highlighting the critical importance of incorporating metabolic dimensions into SO diagnosis. Furthermore, we developed a discriminative model based on the TyG‐WWI index, the performance of which was subsequently validated in two independent external cohorts, CHARLS and ELSA. The findings demonstrate the broad applicability and robustness of this indicator across diverse populations. We further explored the domain‐specific associations between SO and MCI across different cognitive subdomains in three independent cohorts. However, some limitations should be acknowledged. Firstly, the assessment tools for cognitive impairment were not uniform across the included databases, which may introduce heterogeneity and potential measurement bias. Secondly, the enrollment periods for the two independent external cohorts differed slightly because of inherent data limitations. Although we restricted the inclusion timeframe to minimise this discrepancy, a residual gap of approximately 3 years remained and might have introduced potential bias and affected the comparability of the findings. During this period, changes in lifestyle, environmental factors and clinical practices may have occurred, potentially influencing the observed results. Furthermore, the longitudinal cohorts used for external validation were conducted earlier than the cross‐sectional development cohort. Given this temporal misalignment, the model's applicability to future populations remains to be further validated. Thirdly, owing to the inherent limitations of the database, missing data were present for certain covariates. Although multiple imputation was performed to address this issue, we cannot rule out the possibility that selection bias may persist despite these efforts. Furthermore, despite the extensive adjustment for known confounders, residual confounding from other unmeasured or imperfectly measured variables remains a possibility.

## 5 Conclusion

This study demonstrates that a metabolically oriented definition of SO, integrating sarcopenia with an elevated TyG‐WWI, is significantly associated with MCI among community‐dwelling older people in China. This metabolically oriented definition of SO proved to be a more effective screening tool than traditional body composition‐based criteria. We also developed a TyG‐WWI‐based model and confirmed its robustness through external validation in diverse populations.

## Funding

This work was supported by the National Natural Science Foundation of China “Mechanism of BCAT2‐mediated branched‐chain amino acid metabolism inhibiting ferroptosis in the progression of sarcopenia alleviated by intestinal Parabacteroides merdae” (No. 82372575), the Shanghai “Rising Stars of Medical Talent” Youth Development Program for Outstanding Youth Medical Talents (SHWSRS2025–71), the Research Projects of Shanghai Municipal Health Commission “Research on early screening and intervention strategies for cognitive impairment in community‐dwelling older people with sarcopenia” (No. 20254Z0006) and the “Research physician” program of Shanghai Jiao Tong University School of Medicine, “Early screening, risk prediction and rehabilitation intervention of sarcopenia and cognitive impairment in the elderly in the community” (No.20250515).

## Ethics Statement

The study was conducted in accordance with the Declaration of Helsinki and approved by the Ethics Committee of Chongming Hospital Affiliated to Shanghai University of Medicine and Health Sciences (no. CMEC‐2025‐KT‐45). The ELSA was approved by the London Multicentre Research Ethics Committee. All participants provided written informed consent prior to enrollment. The authors confirm adherence to the ethical guidelines for authorship and publishing as stipulated by the Journal of Cachexia, Sarcopenia and Muscle.

## Conflicts of Interest

The authors declare no conflicts of interest.

## Supporting information


**Figure S1:** RCS of association between obesity indices and MCI defined by MOCA or MMSE in sarcopenia.
**Figure S2:** LASSO regression coefficient path plot.
**Figure S3:** LASSO regression cross‐validation results.
**Figure S4:** The model's interpretation.
**Table S1:** Missing data and miss rates of covariates.
**Table S2:** ROC‐derived optimal cut‐off points of TyG‐related obesity indices for obesity (defined by PBF/VFA) based on the Youden index.
**TABLE S3:** Baseline characteristics of participants stratified by sarcopenia and obesity defined by PBF or VFA.
**Table S4:** Baseline characteristics of participants stratified by sarcopenia and obesity defined by TyG‐BMI.
**Table S5:** Baseline characteristics of participants stratified by sarcopenia and obesity defined by TyG‐WC.
**Table S6:** Baseline characteristics of participants stratified by sarcopenia and obesity defined by TyG‐WHR.
**Table S7:** Baseline characteristics of participants stratified by sarcopenia and obesity defined by TyG‐WHtR.
**Table S8:** Baseline characteristics of participants stratified by sarcopenia and obesity defined by TyG‐ABSI.
**Table S9:** Baseline characteristics of participants stratified by sarcopenia and obesity defined by TyG‐BRI.
**Table S10:** Baseline characteristics of participants stratified by sarcopenia and obesity defined by TyG‐WWI.
**Table S11:** Basic characteristics of participants by sarcopenia and obesity defined by TyG‐WWI in CHARLS.
**Table S12:** Basic characteristics of participants by sarcopenia and obesity defined by TyG‐WWI in ELSA.
**Table S13:** Logistic regression analysis of the association between SO and MCI defined by MMSE.
**Table S14:** Logistic regression analysis of the association between SO and MCI defined by MOCA.
**Table S15:** The ability of obesity indicators in identifying mild cognitive impairment defined by MMSE in older people with sarcopenia.
**Table S16:** The ability of obesity indicators in identifying mild cognitive impairment defined by MOCA in older people with sarcopenia.
**Table S17:** The ability of obesity indicators in identifying mild cognitive impairment defined by MOCA in women with sarcopenia.
**Table S18:** The ability of obesity indicators in identifying mild cognitive impairment defined by MOCA in men with sarcopenia.
**Table S19:** The ability of obesity indicators in identifying mild cognitive impairment defined by MOCA with sarcopenia aged 60–75.
**Table S20:** The ability of obesity indicators in identifying mild cognitive impairment defined by MOCA with sarcopenia aged 75 above.
**Table S21:** The ability of obesity indicators in identifying mild cognitive impairment defined by MMSE or MOCA in older people with sarcopenia.
**Table S22:** The ability of obesity indicators in identifying mild cognitive impairment defined by MMSE or MOCA in women with sarcopenia.
**Table S23:** The ability of obesity indicators in identifying mild cognitive impairment defined by MMSE or MOCA in men with sarcopenia.
**Table S24:** The ability of obesity indicators in identifying mild cognitive impairment defined by MMSE or MOCA with sarcopenia aged 60–75.
**Table S25:** The ability of obesity indicators in identifying mild cognitive impairment defined by MMSE or MOCA with sarcopenia aged 75 above.
**Table S26:** Subgroup analysis of the association between sarcopenic obesity (defined by the combination of sarcopenia and TyG‐WWI) and mild cognitive impairment defined by MOCA or MMSE.
**Table S27:** Association between SO (defined by sarcopenia and TyG‐WWI) and cognitive subdomains in the Chongming cohort (assessed by MoCA).
**Table S28:** Association between SO (defined by sarcopenia and TyG‐WWI) and cognitive subdomains in the CHARLS cohort (Wave 3, assessed by AACD criteria).
**Table S29:** Association between SO (defined by sarcopenia and TyG‐WWI) and cognitive subdomains in the ELSA cohort (Wave 6, assessed by AACD criteria).
**Table S30:** The mediating effect of inflammatory markers on the association between sarcopenic obesity (defined by the combination of sarcopenia and TyG‐WWI) and mild cognitive impairment defined by MOCA or MMSE.

**Data S1:** Supporting information.
