# Supplementary material for: Triglyceride–Glucose–Waist‐to‐Weight Index: Novel Biomarker for Mild Cognitive Impairment in Older People With Sarcopenic Obesity
Source: J Cachexia Sarcopenia Muscle. 2026 May 11;17(3):e70307. doi: 10.1002/jcsm.70307 (PMC13161463; doi:10.1002/jcsm.70307)
Supplement: Supplementary file 1 — Figure S1: RCS of association between obesity indices and MCI defined by MOCA or MMSE in sarcopenia. Figure S2: LASSO regression coefficient path plot. Figure S3: LASSO regression cross‐validation results. Figure S4: The model's interpretation. Table S1: Missing data and miss rates of covariates. Table S2: ROC‐derived optimal cut‐off points of TyG‐related obesity indices for obesity (defined by PBF/VFA) based on the Youden index. TABLE S3: Baseline characteristics of participants stratified by sarcopenia and obesity defined by PBF or VFA. Table S4: Baseline characteristics of participants stratified by sarcopenia and obesity defined by TyG‐BMI. Table S5: Baseline characteristics of participants stratified by sarcopenia and obesity defined by TyG‐WC. Table S6: Baseline characteristics of participants stratified by sarcopenia and obesity defined by TyG‐WHR. Table S7: Baseline characteristics of participants stratified by sarcopenia and obesity defined by TyG‐WHtR. Table S8: Baseline characteristics of participants stratified by sarcopenia and obesity defined by TyG‐ABSI. Table S9: Baseline characteristics of participants stratified by sarcopenia and obesity defined by TyG‐BRI. Table S10: Baseline characteristics of participants stratified by sarcopenia and obesity defined by TyG‐WWI. Table S11: Basic characteristics of participants by sarcopenia and obesity defined by TyG‐WWI in CHARLS. Table S12: Basic characteristics of participants by sarcopenia and obesity defined by TyG‐WWI in ELSA. Table S13: Logistic regression analysis of the association between SO and MCI defined by MMSE. Table S14: Logistic regression analysis of the association between SO and MCI defined by MOCA. Table S15: The ability of obesity indicators in identifying mild cognitive impairment defined by MMSE in older people with sarcopenia. Table S16: The ability of obesity indicators in identifying mild cognitive impairment defined by MOCA in older people with sarcopenia. Table S17: The ability of obe [file JCSM-17-e70307-s002.docx]

**Supplementary Information for “Triglyceride-glucose-waist-to-weight index: novel biomarker for mild cognitive impairment in older people with sarcopenic obesity”**

**Journal name:** Journal of Cachexia, Sarcopenia and Muscle

**Author names and affiliations**

Yue Xing^1,2,#^, Qian Liu^3,#^, Xiangfeng He^1,#^, Lin Ma^1^, Yanping Song^1^, Nan Chen PhD ^1,3*^

^1^ Department of Rehabilitation, Chongming Hospital Affiliated to Shanghai University of Medicine and Health Sciences, Shanghai, China

^2^ Shanghai university of sport, Shanghai, China

^3^ Department of Rehabilitation, Xinhua Hospital Affiliated to Shanghai Jiaotong University School of Medicine, Shanghai, China

^#^ Yue Xing, Qian Liu and Xiangfeng He contributed equally to this study

* Corresponding author:

Nan Chen, PhD, Department of Rehabilitation, Chongming Hospital Affiliated to Shanghai University of Medicine and Health Sciences, Shanghai, China; Department of Rehabilitation, Xinhua Hospital Affiliated to Shanghai Jiaotong University School of Medicine, Shanghai, China; Email: chennanreh2020@126.com

**Supplementary method**

**Supplementary Table list:**

**Table S1. Missing data and miss rates of covariates.**

**Table S2. ROC-derived optimal cut-off points of TyG-related obesity indices for obesity (defined by PBF/VFA) based**

**on the Youden index.**

**Table S3. Baseline characteristics of participants stratified by sarcopenia and obesity defined by PBF or VFA.**

**Table S4. Baseline characteristics of participants stratified by sarcopenia and obesity defined by TyG-BMI.**

**Table S5. Baseline characteristics of participants stratified by sarcopenia and obesity defined by TyG-WC.**

**Table S6. Baseline characteristics of participants stratified by sarcopenia and obesity defined by TyG-WHR.**

**Table S7. Baseline characteristics of participants stratified by sarcopenia and obesity defined by TyG-WHtR.**

**Table S8. Baseline characteristics of participants stratified by sarcopenia and obesity defined by TyG-ABSI.**

**Table S9. Baseline characteristics of participants stratified by sarcopenia and obesity defined by TyG-BRI.**

**Table S10. Baseline characteristics of participants stratified by sarcopenia and obesity defined by TyG-WWI.**

**Table S11. Basic characteristics of participants by sarcopenia and obesity defined by TyG-WWI in CHARLS.**

**Table S12. Basic characteristics of participants by sarcopenia and obesity defined by TyG-WWI in ELSA.**

**Table S13. Logistic regression analysis of the association between SO and MCI defined by MMSE.**

**Table S14. Logistic regression analysis of the association between SO and MCI defined by MOCA.**

**Table S15. The ability of obesity indicators in identifying mild cognitive impairment defined by MMSE in older people with sarcopenia.**

**Table S16. The ability of obesity indicators in identifying mild cognitive impairment defined by MOCA in older people with sarcopenia.**

**Table S17. The ability of obesity indicators in identifying mild cognitive impairment defined by MOCA in women with sarcopenia.**

**Table S18. The ability of obesity indicators in identifying mild cognitive impairment defined by MOCA in men with sarcopenia.**

**Table S19. The ability of obesity indicators in identifying mild cognitive impairment defined by MOCA with sarcopenia aged 60-75.**

**Table S20. The ability of obesity indicators in identifying mild cognitive impairment defined by MOCA with sarcopenia aged 75 above.**

**Table S21. The ability of obesity indicators in identifying mild cognitive impairment defined by MMSE or MOCA in older people with sarcopenia.**

**Table S22. The ability of obesity indicators in identifying mild cognitive impairment defined by MMSE or MOCA in women with sarcopenia.**

**Table S23. The ability of obesity indicators in identifying mild cognitive impairment defined by MMSE or MOCA in men with sarcopenia.**

**Table S24. The ability of obesity indicators in identifying mild cognitive impairment defined by MMSE or MOCA with sarcopenia aged 60-75.**

**Table S25. The ability of obesity indicators in identifying mild cognitive impairment defined by MMSE or MOCA with sarcopenia aged 75 above.**

**Table S26. Subgroup analysis of the association between sarcopenic obesity (defined by the combination of sarcopenia and TyG-WWI) and mild cognitive impairment defined by MOCA or MMSE.**

**Table S27. Association between SO (defined by sarcopenia and TyG-WWI) and cognitive subdomains in the Chongming cohort (assessed by MoCA)**

**Table S28. Association between SO (defined by sarcopenia and TyG-WWI) and cognitive subdomains in the CHARLS cohort (Wave 3, assessed by AACD criteria)**

**Table S29. Association between SO (defined by sarcopenia and TyG-WWI) and cognitive subdomains in the ELSA cohort (Wave 6, assessed by AACD criteria)**

**Table S30. The mediating effect of inflammatory markers on the association between sarcopenic obesity (defined by the combination of sarcopenia and TyG-WWI) and mild cognitive impairment defined by MOCA or MMSE.**

**Figure S1. RCS of association between obesity indices and MCI defined by MOCA or MMSE in sarcopenia.**

**Figure S2. LASSO regression coefficient path plot.**

**Figure S3. LASSO regression cross validation results.**

**Figure S4. The model’s interpretation.**

**Supplementary method**

**Sarcopenia**

The diagnosis of sarcopenia in the Shanghai Chongming study was based on the AWGS 2019 criteria. Similarly, the CHARLS cohort adopted the AWGS 2019 framework for sarcopenia diagnosis. However, due to the absence of direct appendicular skeletal muscle mass index (ASMI) measurements in CHARLS, ASM was estimated using a previously validated prediction equation . The derived ASM was then divided by the square of height to calculate ASMI, with the lowest 20% of ASMI values classified as low muscle mass. In accordance with AWGS 2019, low handgrip strength was defined as <28 kg for men and <18 kg for women. Physical performance measures included gait speed, the five-times sit-to-stand test, and the short physical performance battery (SPPB). Low physical performance was defined as gait speed <1.0 m/s, five-times sit-to-stand time ≥12 seconds, or SPPB score <9 points, consistent with AWGS 2019 recommendations. Sarcopenia was defined as the presence of both low muscle mass and low muscle strength, or low physical performance. In the ELSA cohort, sarcopenia was defined according to the EWGSOP framework, with low handgrip strength defined as <27 kg for men and <16 kg for women [1].

ASM = 0.193 * weight (kg) + 0.107 * height (cm) - 4.157 * gender - 0.037 * age - 2.631

If male, gender was set to 1, otherwise to 0.

**MCI**

Cognitive function among CHARLS participants was assessed across three domains: orientation, memory, and executive function. The orientation domain was evaluated through five items—current year, month, date, day of the week, and season—with each correct response scoring 1 point, yielding a maximum of 5 points. Memory was measured using a 10-word recall test, in which participants were asked to recall the words immediately after presentation and again after a delay following other cognitive tasks. Each correctly recalled word was assigned 1 point, resulting in a maximum memory score of 20 points (10 points for immediate recall and 10 for delayed recall). Executive function was examined through two tasks: serial subtraction and drawing. In the serial subtraction task, participants were required to subtract 7 from 100 sequentially five times, with each correct subtraction earning 1 point (up to 5 points). In the drawing task, participants were shown an image of two overlapping pentagons and asked to reproduce it; a correct drawing was awarded 1 point. The global cognitive score was calculated as the sum of scores from the orientation (5 points), serial subtraction (5 points), memory (20 points), and figure drawing (1 point) domains, with a maximum total score of 31 points [2].

As executive function was not assessed in wave 6 of the ELSA study, cognitive function was evaluated across the two available core domains: orientation and memory. The orientation domain consisted of four items—year, month, date, and day of the week—with each correct response assigned 1 point, resulting in a maximum of 4 points. Memory was evaluated using a 10-word list recall task. Participants were asked to recall the words immediately after hearing them (immediate recall) and again after a delay during which other cognitive tests were administered (delayed recall). Each correctly recalled word was scored 1 point, leading to a maximum memory score of 20 points (10 points for each recall phase). The global cognitive score was derived as the sum of the orientation (4 points) and memory (20 points) domain scores, with a maximum total of 24 points [3].

**Covariates and others**

The covariates in this analysis were defined as follows: age (continuous, calculated as the participant's actual age at the time of the interview); gender (male or female); educational level (below high school, high school, or above high school); marital status [married or Others (never married, divorced, separated, or widowed)]; living status (living alone, living with spouse, living with children, or living with both spouse and children; data unavailable in CHARLS and ELSA); smoking status and alcohol consumption (never, former, or current for each); physical activity (low, moderate, or high) [4, 5]. Sleep duration was defined as the actual number of hours slept per night, rather than time spent in bed, and was categorized into three groups: <6 hours, 6–8 hours, and >8 hours. HDL-C, LDL-C, and TC were analyzed as continuous variables. Additionally, self-reported information on comorbidities, including hypertension, diabetes, stroke, heart disease, and liver disease, was collected. Liver disease was not available in the ELSA dataset.

**Reference**

1. Hamer M, O'Donovan G. Sarcopenic obesity, weight loss, and mortality: the English Longitudinal Study of Ageing. Am J Clin Nutr. 2017;106:125-9. doi:10.3945/ajcn.117.152488

2. Hu Y, Peng W, Ren R, Wang Y, Wang G. Sarcopenia and mild cognitive impairment among elderly adults: The first longitudinal evidence from CHARLS. J Cachexia Sarcopenia Muscle. 2022;13:2944-52. doi:10.1002/jcsm.13081

3. Wang Y, Wu Z, Duan L, Liu S, Chen R, Sun T, et al. Digital exclusion and cognitive impairment in older people: findings from five longitudinal studies. BMC Geriatr. 2024;24:406. doi:10.1186/s12877-024-05026-w

4. Qu NN, Li KJ. [Study on the reliability and validity of international physical activity questionnaire (Chinese Vision, IPAQ)]. Zhonghua Liu Xing Bing Xue Za Zhi. 2004;25:265-8.

5. Hamer M, Lavoie KL, Bacon SL. Taking up physical activity in later life and healthy ageing: the English longitudinal study of ageing. Br J Sports Med. 2014;48:239-43. doi:10.1136/bjsports-2013-092993

**Table S1. Missing data and miss rates of covariates.**

|  | **Chongming, Shanghai** | | **CHARLS** | | **ELSA** | |
| --- | --- | --- | --- | --- | --- | --- |
| **Variables** | **Missing** | **Missing Rate (%)** | **Missing** | **Missing Rate (%)** | **Missing** | **Missing Rate (%)** |
| Age | 0 | 0 | 0 | 0 | 0 | 0 |
| Gender | 0 | 0 | 0 | 0 | 0 | 0 |
| Education | 0 | 0 | 0 | 0 | 157 | 8.6 |
| Marital | 0 | 0 | 0 | 0 | 1 | 0.05 |
| Living status | 0 | 0 | - | - | - | - |
| Smoke | 0 | 0 | 1 | <0.1 | 0 | 0 |
| Drink | 0 | 0 | 3 | <0.1 | 89 | 4.88 |
| Physical activity | 12 | 0.5 | 1766 | 52 | 1 | 0.05 |
| Sleep | 12 | 0.5 | 14 | 0.4 | 1 | 0.05 |
| Hypertension | 0 | 0 | 209 | 6.1 | 0 | 0 |
| Diabetes | 0 | 0 | 247 | 7.2 | 0 | 0 |
| Stroke | 0 | 0 | 209 | 6.1 | 0 | 0 |
| Liver disease | 0 | 0 | 224 | 6.2 | - | - |
| Heart disease | 0 | 0 | 230 | 6.7 | 0 | 0 |
| LDL-C | 0 | 0 | 0 | 0 | 8 | 0.44 |
| HDL-C | 0 | 0 | 0 | 0 | 0 | 0 |
| TC | 0 | 0 | 0 | 0 | 0 | 0 |
| Abbreviation: CHARLS, China Health and Retirement Longitudinal Study; ELSA, English Longitudinal Study of Ageing; LDL-C, low-density lipoprotein cholesterol; HDL-C, high-density lipoprotein cholesterol; TC, total cholesterol. | | | | | | |

**Table S2. ROC-derived optimal cut-off points of TyG-related obesity indices for obesity (defined by PBF/VFA) based**

**on the Youden index.**

|  | **AUC (95%CI)** | **Accuracy (95%CI)** | **Sensitivity (95%CI)** | **Specificity (95%CI)** | **PPV (95%CI)** | **NPV (95%CI)** | **Cut off** |
| --- | --- | --- | --- | --- | --- | --- | --- |
| TyG-BMI | 0.89 (0.87-0.91) | 0.80 (0.79-0.82) | 0.84 (0.80 - 0.87) | 0.79 (0.78 - 0.81) | 0.49 (0.46 - 0.53) | 0.95 (0.94 - 0.96) | 200.48 |
| TyG-WC | 0.83 (0.80-0.85) | 0.79 (0.78-0.81) | 0.68 (0.64 - 0.72) | 0.82 (0.80 - 0.84) | 0.47 (0.43 - 0.51) | 0.92 (0.90 - 0.93) | 720.89 |
| TYG-WHR | 0.74 (0.72-0.77) | 0.76 (0.75-0.78) | 0.60 (0.55 - 0.64) | 0.80 (0.79 - 0.82) | 0.42 (0.38 - 0.46) | 0.89 (0.88 - 0.91) | 7.37 |
| TyG-WHtR | 0.82 (0.80-0.84) | 0.76 (0.74-0.77) | 0.73 (0.69 - 0.78) | 0.76 (0.74 - 0.78) | 0.42 (0.39 - 0.45) | 0.92 (0.91 - 0.94) | 4.59 |
| TyG-ABSI | 0.61 (0.58-0.64) | 0.72 (0.70-0.74) | 0.40 (0.36 - 0.45) | 0.80 (0.78 - 0.81) | 0.32 (0.28 - 0.36) | 0.85 (0.83 - 0.87) | 6.81 |
| TyG-BRI | 0.83 (0.81-0.85) | 0.75 (0.73-0.76) | 0.76 (0.72 - 0.80) | 0.74 (0.72 - 0.76) | 0.41 (0.38 - 0.45) | 0.93 (0.92 - 0.94) | 35.13 |
| TyG-WWI | 0.68 (0.65-0.71) | 0.67 (0.65-0.69) | 0.61 (0.57 - 0.66) | 0.68 (0.66 - 0.70) | 0.31 (0.28 - 0.34) | 0.88 (0.87 - 0.90) | 94.74 |
| Abbreviation: AUC, area under the curve; PPV, positive predictive value; NPV, negative predictive value; TyG-BMI, triglyceride-glucose-body mass index; TyG-WC, triglyceride-glucose-waist circumference; TyG-WHR, triglyceride-glucose-waist-to-hip ratio; TyG-WHtR, triglyceride-glucose-waist-to-height ratio; TyG-ABSI, triglyceride-glucose-a body shape index; TyG-BRI: triglyceride-glucose-body roundness index; TyG-WWI, triglyceride-glucose-waist-to-weight index. | | | | | | | |

**Table S3. Baseline characteristics of participants stratified by sarcopenia and obesity defined by PBF or VFA.**

| **Variables** | **Total (n = 2326)** | **N (n = 366)** | **O (n = 1680)** | **S (n = 79)** | **S+PBF/VFA (n = 201)** | ***P*** |
| --- | --- | --- | --- | --- | --- | --- |
| Age, Mean ± SD | 72.62 ± 5.56 | 71.55 ± 4.61 | 72.33 ± 5.43 | 75.15 ± 6.05 | 75.98 ± 6.45 | **<0.001** |
| Gender, n(%) |  |  |  |  |  | **<0.001** |
| Female | 1301 (55.93) | 257 (70.22) | 890 (52.98) | 54 (68.35) | 100 (49.75) |  |
| Male | 1025 (44.07) | 109 (29.78) | 790 (47.02) | 25 (31.65) | 101 (50.25) |  |
| Education, n(%) |  |  |  |  |  | **0.009** |
| Below high school | 1871 (80.44) | 270 (73.77) | 1361 (81.01) | 66 (83.54) | 174 (86.57) |  |
| High school | 409 (17.58) | 84 (22.95) | 289 (17.20) | 12 (15.19) | 24 (11.94) |  |
| Above high school | 46 (1.98) | 12 (3.28) | 30 (1.79) | 1 (1.27) | 3 (1.49) |  |
| Marital, n(%) |  |  |  |  |  | **0.007** |
| Married | 1956 (84.09) | 310 (84.70) | 1430 (85.12) | 63 (79.75) | 153 (76.12) |  |
| Others | 370 (15.91) | 56 (15.30) | 250 (14.88) | 16 (20.25) | 48 (23.88) |  |
| Living status, n(%) |  |  |  |  |  | **0.039** |
| Living alone | 328 (14.10) | 55 (15.03) | 225 (13.39) | 12 (15.19) | 36 (17.91) |  |
| Living with spouse and children | 147 (6.32) | 22 (6.01) | 111 (6.61) | 2 (2.53) | 12 (5.97) |  |
| Living with spouse | 1770 (76.10) | 279 (76.23) | 1292 (76.90) | 61 (77.22) | 138 (68.66) |  |
| Living with children | 81 (3.48) | 10 (2.73) | 52 (3.10) | 4 (5.06) | 15 (7.46) |  |
| Smoke, n(%) |  |  |  |  |  | **0.008** |
| Never | 1657 (71.24) | 287 (78.42) | 1173 (69.82) | 62 (78.48) | 135 (67.16) |  |
| Former | 289 (12.42) | 28 (7.65) | 223 (13.27) | 6 (7.59) | 32 (15.92) |  |
| Now | 380 (16.34) | 51 (13.93) | 284 (16.90) | 11 (13.92) | 34 (16.92) |  |
| Drink, n(%) |  |  |  |  |  | **<0.001** |
| Never | 1624 (69.82) | 285 (77.87) | 1139 (67.80) | 64 (81.01) | 136 (67.66) |  |
| Former | 186 (8.00) | 14 (3.83) | 143 (8.51) | 7 (8.86) | 22 (10.95) |  |
| Now | 516 (22.18) | 67 (18.31) | 398 (23.69) | 8 (10.13) | 43 (21.39) |  |
| Sleep duration, n(%) |  |  |  |  |  | **<0.001** |
| <6 | 503 (21.74) | 99 (27.97) | 340 (20.24) | 28 (35.44) | 36 (17.91) |  |
| 6-8 | 1297 (56.05) | 198 (55.93) | 944 (56.19) | 42 (53.16) | 113 (56.22) |  |
| >8 | 514 (22.21) | 57 (16.10) | 396 (23.57) | 9 (11.39) | 52 (25.87) |  |
| Physical activity, n(%) |  |  |  |  |  | **<0.001** |
| Low | 211 (9.12) | 22 (6.21) | 160 (9.52) | 6 (7.59) | 23 (11.44) |  |
| Moderate | 568 (24.55) | 68 (19.21) | 408 (24.29) | 23 (29.11) | 69 (34.33) |  |
| High | 1535 (66.34) | 264 (74.58) | 1112 (66.19) | 50 (63.29) | 109 (54.23) |  |
| MMSE, n(%) |  |  |  |  |  | 0.428 |
| Normal | 1621 (69.69) | 257 (70.22) | 1177 (70.06) | 57 (72.15) | 130 (64.68) |  |
| Mild cognitive impairment | 705 (30.31) | 109 (29.78) | 503 (29.94) | 22 (27.85) | 71 (35.32) |  |
| MOCA , n(%) |  |  |  |  |  | **0.002** |
| Normal | 781 (33.58) | 146 (39.89) | 563 (33.51) | 23 (29.11) | 49 (24.38) |  |
| Mild cognitive impairment | 1545 (66.42) | 220 (60.11) | 1117 (66.49) | 56 (70.89) | 152 (75.62) |  |
| Hypertension, n(%) |  |  |  |  |  | **<0.001** |
| NO | 817 (35.12) | 188 (51.37) | 498 (29.64) | 51 (64.56) | 80 (39.80) |  |
| YES | 1509 (64.88) | 178 (48.63) | 1182 (70.36) | 28 (35.44) | 121 (60.20) |  |
| Diabetes, n(%) |  |  |  |  |  | **0.049** |
| NO | 1639 (70.46) | 271 (74.04) | 1162 (69.17) | 64 (81.01) | 142 (70.65) |  |
| YES | 687 (29.54) | 95 (25.96) | 518 (30.83) | 15 (18.99) | 59 (29.35) |  |
| Stroke, n(%) |  |  |  |  |  | 0.140 |
| NO | 1732 (74.46) | 289 (78.96) | 1243 (73.99) | 57 (72.15) | 143 (71.14) |  |
| YES | 594 (25.54) | 77 (21.04) | 437 (26.01) | 22 (27.85) | 58 (28.86) |  |
| Liver disease, n(%) |  |  |  |  |  | **<0.001** |
| NO | 497 (21.37) | 116 (31.69) | 302 (17.98) | 27 (34.18) | 52 (25.87) |  |
| YES | 1829 (78.63) | 250 (68.31) | 1378 (82.02) | 52 (65.82) | 149 (74.13) |  |
| Heart disease, n(%) |  |  |  |  |  | 0.148 |
| NO | 2147 (92.30) | 348 (95.08) | 1539 (91.61) | 74 (93.67) | 186 (92.54) |  |
| YES | 179 (7.70) | 18 (4.92) | 141 (8.39) | 5 (6.33) | 15 (7.46) |  |
| LDL-C, Mean ± SD | 2.92 ± 0.83 | 2.81 ± 0.81 | 2.95 ± 0.83 | 2.68 ± 0.73 | 2.91 ± 0.87 | **0.001** |
| HDL-C, Mean ± SD | 1.85 ± 0.49 | 2.10 ± 0.54 | 1.76 ± 0.43 | 2.29 ± 0.58 | 1.92 ± 0.50 | **<0.001** |
| TC, Mean ± SD | 5.37 ± 1.11 | 5.44 ± 1.07 | 5.35 ± 1.11 | 5.45 ± 1.05 | 5.40 ± 1.20 | 0.503 |
| ALT, Mean ± SD | 12.21 ± 6.20 | 11.12 ± 4.96 | 12.64 ± 6.49 | 10.67 ± 6.17 | 11.19 ± 5.30 | **<0.001** |
| AST, Mean ± SD | 22.75 ± 6.93 | 22.57 ± 5.90 | 22.82 ± 7.19 | 23.18 ± 8.17 | 22.31 ± 5.80 | 0.682 |
| WBC, Mean ± SD | 6.64 ± 1.79 | 6.21 ± 1.63 | 6.74 ± 1.79 | 6.08 ± 1.70 | 6.85 ± 2.00 | **<0.001** |
| NEU, Mean ± SD | 3.82 ± 1.42 | 3.58 ± 1.33 | 3.86 ± 1.41 | 3.58 ± 1.40 | 3.98 ± 1.58 | **<0.001** |
| LYM, Mean ± SD | 2.21 ± 0.68 | 2.06 ± 0.58 | 2.25 ± 0.69 | 1.92 ± 0.65 | 2.22 ± 0.69 | **<0.001** |
| NLR, Mean ± SD | 1.87 ± 0.88 | 1.86 ± 0.86 | 1.85 ± 0.84 | 2.11 ± 1.43 | 1.94 ± 0.95 | **0.048** |
| PLR, Mean ± SD | 100.23 ± 35.66 | 104.40 ± 35.84 | 98.41 ± 34.25 | 119.94 ± 54.33 | 100.19 ± 35.09 | **<0.001** |
| SII, Mean ± SD | 387.13 ± 222.09 | 379.35 ± 211.83 | 383.61 ± 206.54 | 455.60 ± 425.98 | 403.73 ± 245.53 | **0.023** |
| Abbreviation: N, normal; O, obesity; S, sarcopenia; PBF, body fat percentage; VFA, visceral fat area; MMSE, mini-mental state examination; MOCA, montreal cognitive assessment; LDL-C, low-density lipoprotein cholesterol; HDL-C, high-density lipoprotein cholesterol; TC, total cholesterol; ALT, alanine aminotransferase; AST, aspartate aminotransferase; WBC, white blood cell; NEU, neutrophil; LYM, lymphocyt; NLR, neutrophil-to-lymphocyte ratio; PLR, platelet-to-lymphocyte ratio; SII, systemic immune-inflammation index. | | | | | | |

**Table S4. Baseline characteristics of participants stratified by sarcopenia and obesity defined by TyG-BMI.**

| **Variables** | **Total (n = 2326)** | **N (n = 571)** | **O (n = 1475)** | **S (n = 188)** | **S+TyG-BMI (n = 92)** | ***P*** |
| --- | --- | --- | --- | --- | --- | --- |
| Age, Mean ± SD | 72.62 ± 5.56 | 72.06 ± 5.26 | 72.24 ± 5.32 | 75.34 ± 6.11 | 76.57 ± 6.73 | **<0.001** |
| Gender, n(%) |  |  |  |  |  | 0.701 |
| Female | 1301 (55.93) | 322 (56.39) | 825 (55.93) | 99 (52.66) | 55 (59.78) |  |
| Male | 1025 (44.07) | 249 (43.61) | 650 (44.07) | 89 (47.34) | 37 (40.22) |  |
| Education, n(%) |  |  |  |  |  | 0.067 |
| Below high school | 1871 (80.44) | 436 (76.36) | 1195 (81.02) | 161 (85.64) | 79 (85.87) |  |
| High school | 409 (17.58) | 121 (21.19) | 252 (17.08) | 25 (13.30) | 11 (11.96) |  |
| Above high school | 46 (1.98) | 14 (2.45) | 28 (1.90) | 2 (1.06) | 2 (2.17) |  |
| Marital, n(%) |  |  |  |  |  | **0.002** |
| Married | 1956 (84.09) | 481 (84.24) | 1259 (85.36) | 150 (79.79) | 66 (71.74) |  |
| Others | 370 (15.91) | 90 (15.76) | 216 (14.64) | 38 (20.21) | 26 (28.26) |  |
| Living status, n(%) |  |  |  |  |  | **0.022** |
| Living alone | 328 (14.10) | 78 (13.66) | 202 (13.69) | 28 (14.89) | 20 (21.74) |  |
| Living with spouse and children | 147 (6.32) | 39 (6.83) | 94 (6.37) | 10 (5.32) | 4 (4.35) |  |
| Living with spouse | 1770 (76.10) | 433 (75.83) | 1138 (77.15) | 139 (73.94) | 60 (65.22) |  |
| Living with children | 81 (3.48) | 21 (3.68) | 41 (2.78) | 11 (5.85) | 8 (8.70) |  |
| Smoke, n(%) |  |  |  |  |  | 0.066 |
| Never | 1657 (71.24) | 414 (72.50) | 1046 (70.92) | 122 (64.89) | 75 (81.52) |  |
| Former | 289 (12.42) | 59 (10.33) | 192 (13.02) | 31 (16.49) | 7 (7.61) |  |
| Now | 380 (16.34) | 98 (17.16) | 237 (16.07) | 35 (18.62) | 10 (10.87) |  |
| Drink, n(%) |  |  |  |  |  | 0.057 |
| Never | 1624 (69.82) | 418 (73.20) | 1006 (68.20) | 132 (70.21) | 68 (73.91) |  |
| Former | 186 (8.00) | 39 (6.83) | 118 (8.00) | 23 (12.23) | 6 (6.52) |  |
| Now | 516 (22.18) | 114 (19.96) | 351 (23.80) | 33 (17.55) | 18 (19.57) |  |
| Sleep duration, n(%) |  |  |  |  |  | **0.005** |
| <6 | 503 (21.74) | 150 (26.83) | 289 (19.59) | 49 (26.06) | 15 (16.30) |  |
| 6-8 | 1297 (56.05) | 298 (53.31) | 844 (57.22) | 104 (55.32) | 51 (55.43) |  |
| >8 | 514 (22.21) | 111 (19.86) | 342 (23.19) | 35 (18.62) | 26 (28.26) |  |
| Physical activity, n(%) |  |  |  |  |  | **<0.001** |
| Low | 211 (9.12) | 35 (6.26) | 147 (9.97) | 18 (9.57) | 11 (11.96) |  |
| Moderate | 568 (24.55) | 111 (19.86) | 365 (24.75) | 60 (31.91) | 32 (34.78) |  |
| High | 1535 (66.34) | 413 (73.88) | 963 (65.29) | 110 (58.51) | 49 (53.26) |  |
| MMSE, n(%) |  |  |  |  |  | 0.102 |
| Normal | 1621 (69.69) | 385 (67.43) | 1049 (71.12) | 131 (69.68) | 56 (60.87) |  |
| Mild cognitive impairment | 705 (30.31) | 186 (32.57) | 426 (28.88) | 57 (30.32) | 36 (39.13) |  |
| MOCA , n(%) |  |  |  |  |  | **0.003** |
| Normal | 781 (33.58) | 212 (37.13) | 497 (33.69) | 55 (29.26) | 17 (18.48) |  |
| Mild cognitive impairment | 1545 (66.42) | 359 (62.87) | 978 (66.31) | 133 (70.74) | 75 (81.52) |  |
| Hypertension, n(%) |  |  |  |  |  | **<0.001** |
| NO | 817 (35.12) | 297 (52.01) | 389 (26.37) | 98 (52.13) | 33 (35.87) |  |
| YES | 1509 (64.88) | 274 (47.99) | 1086 (73.63) | 90 (47.87) | 59 (64.13) |  |
| Diabetes, n(%) |  |  |  |  |  | **<0.001** |
| NO | 1639 (70.46) | 470 (82.31) | 963 (65.29) | 151 (80.32) | 55 (59.78) |  |
| YES | 687 (29.54) | 101 (17.69) | 512 (34.71) | 37 (19.68) | 37 (40.22) |  |
| Stroke, n(%) |  |  |  |  |  | **0.030** |
| NO | 1732 (74.46) | 441 (77.23) | 1091 (73.97) | 142 (75.53) | 58 (63.04) |  |
| YES | 594 (25.54) | 130 (22.77) | 384 (26.03) | 46 (24.47) | 34 (36.96) |  |
| Liver disease, n(%) |  |  |  |  |  | **<0.001** |
| NO | 497 (21.37) | 199 (34.85) | 219 (14.85) | 61 (32.45) | 18 (19.57) |  |
| YES | 1829 (78.63) | 372 (65.15) | 1256 (85.15) | 127 (67.55) | 74 (80.43) |  |
| Heart disease, n(%) |  |  |  |  |  | 0.564 |
| NO | 2147 (92.30) | 534 (93.52) | 1353 (91.73) | 175 (93.09) | 85 (92.39) |  |
| YES | 179 (7.70) | 37 (6.48) | 122 (8.27) | 13 (6.91) | 7 (7.61) |  |
| LDL-C, Mean ± SD | 2.92 ± 0.83 | 2.70 ± 0.75 | 3.02 ± 0.84 | 2.71 ± 0.79 | 3.12 ± 0.88 | **<0.001** |
| HDL-C, Mean ± SD | 1.85 ± 0.49 | 2.14 ± 0.48 | 1.70 ± 0.41 | 2.13 ± 0.55 | 1.80 ± 0.47 | **<0.001** |
| TC, Mean ± SD | 5.37 ± 1.11 | 5.32 ± 1.01 | 5.39 ± 1.14 | 5.33 ± 1.11 | 5.58 ± 1.24 | 0.165 |
| ALT, Mean ± SD | 12.21 ± 6.20 | 10.83 ± 4.04 | 12.96 ± 6.85 | 10.79 ± 5.41 | 11.57 ± 5.83 | **<0.001** |
| AST, Mean ± SD | 22.75 ± 6.93 | 22.79 ± 5.54 | 22.77 ± 7.46 | 22.81 ± 6.90 | 22.03 ± 5.79 | 0.794 |
| WBC, Mean ± SD | 6.64 ± 1.79 | 6.24 ± 1.67 | 6.80 ± 1.79 | 6.32 ± 1.73 | 7.26 ± 2.21 | **<0.001** |
| NEU, Mean ± SD | 3.82 ± 1.42 | 3.61 ± 1.40 | 3.89 ± 1.39 | 3.66 ± 1.43 | 4.29 ± 1.68 | **<0.001** |
| LYM, Mean ± SD | 2.21 ± 0.68 | 2.05 ± 0.55 | 2.28 ± 0.71 | 2.03 ± 0.64 | 2.35 ± 0.75 | **<0.001** |
| NLR, Mean ± SD | 1.87 ± 0.88 | 1.88 ± 0.88 | 1.84 ± 0.82 | 2.00 ± 1.22 | 1.95 ± 0.82 | 0.081 |
| PLR, Mean ± SD | 100.23 ± 35.66 | 105.29 ± 36.45 | 97.23 ± 33.61 | 108.93 ± 44.69 | 99.28 ± 36.23 | **<0.001** |
| SII, Mean ± SD | 387.13 ± 222.09 | 385.72 ± 220.11 | 381.74 ± 202.40 | 414.53 ± 339.67 | 426.21 ± 229.18 | 0.086 |
| Abbreviation: N, normal; O, obesity; S, sarcopenia; TyG-BMI, triglyceride-glucose-body mass index; MMSE, mini-mental state examination; MOCA, montreal cognitive assessment; LDL-C, low-density lipoprotein cholesterol; HDL-C, high-density lipoprotein cholesterol; TC, total cholesterol; ALT, alanine aminotransferase; AST, aspartate aminotransferase; WBC, white blood cell; NEU, neutrophil; LYM, lymphocyt; NLR, neutrophil-to-lymphocyte ratio; PLR, platelet-to-lymphocyte ratio; SII, systemic immune-inflammation index. | | | | | | |

**Table S5. Baseline characteristics of participants stratified by sarcopenia and obesity defined by TyG-WC.**

| **Variables** | **Total (n = 2326)** | **N (n = 501)** | **O (n = 1545)** | **S (n = 143)** | **S+TyG-WC (n = 137)** | ***P*** |
| --- | --- | --- | --- | --- | --- | --- |
| Age, Mean ± SD | 72.62 ± 5.56 | 71.48 ± 5.10 | 72.42 ± 5.35 | 74.80 ± 6.07 | 76.72 ± 6.48 | **<0.001** |
| Gender, n(%) |  |  |  |  |  | **0.002** |
| Female | 1301 (55.93) | 317 (63.27) | 830 (53.72) | 81 (56.64) | 73 (53.28) |  |
| Male | 1025 (44.07) | 184 (36.73) | 715 (46.28) | 62 (43.36) | 64 (46.72) |  |
| Education, n(%) |  |  |  |  |  | **0.044** |
| Below high school | 1871 (80.44) | 380 (75.85) | 1251 (80.97) | 121 (84.62) | 119 (86.86) |  |
| High school | 409 (17.58) | 111 (22.16) | 262 (16.96) | 20 (13.99) | 16 (11.68) |  |
| Above high school | 46 (1.98) | 10 (2.00) | 32 (2.07) | 2 (1.40) | 2 (1.46) |  |
| Marital, n(%) |  |  |  |  |  | **<0.001** |
| Married | 1956 (84.09) | 418 (83.43) | 1322 (85.57) | 118 (82.52) | 98 (71.53) |  |
| Others | 370 (15.91) | 83 (16.57) | 223 (14.43) | 25 (17.48) | 39 (28.47) |  |
| Living status, n(%) |  |  |  |  |  | **0.002** |
| Living alone | 328 (14.10) | 71 (14.17) | 209 (13.53) | 17 (11.89) | 31 (22.63) |  |
| Living with spouse and children | 147 (6.32) | 33 (6.59) | 100 (6.47) | 8 (5.59) | 6 (4.38) |  |
| Living with spouse | 1770 (76.10) | 375 (74.85) | 1196 (77.41) | 110 (76.92) | 89 (64.96) |  |
| Living with children | 81 (3.48) | 22 (4.39) | 40 (2.59) | 8 (5.59) | 11 (8.03) |  |
| Smoke, n(%) |  |  |  |  |  | 0.066 |
| Never | 1657 (71.24) | 385 (76.85) | 1075 (69.58) | 100 (69.93) | 97 (70.80) |  |
| Former | 289 (12.42) | 43 (8.58) | 208 (13.46) | 20 (13.99) | 18 (13.14) |  |
| Now | 380 (16.34) | 73 (14.57) | 262 (16.96) | 23 (16.08) | 22 (16.06) |  |
| Drink, n(%) |  |  |  |  |  | **0.013** |
| Never | 1624 (69.82) | 377 (75.25) | 1047 (67.77) | 103 (72.03) | 97 (70.80) |  |
| Former | 186 (8.00) | 31 (6.19) | 126 (8.16) | 17 (11.89) | 12 (8.76) |  |
| Now | 516 (22.18) | 93 (18.56) | 372 (24.08) | 23 (16.08) | 28 (20.44) |  |
| Sleep duration, n(%) |  |  |  |  |  | **0.003** |
| <6 | 503 (21.74) | 135 (27.44) | 304 (19.71) | 38 (26.57) | 26 (18.98) |  |
| 6-8 | 1297 (56.05) | 265 (53.86) | 877 (56.87) | 81 (56.64) | 74 (54.01) |  |
| >8 | 514 (22.21) | 92 (18.70) | 361 (23.41) | 24 (16.78) | 37 (27.01) |  |
| Physical activity, n(%) |  |  |  |  |  | **<0.001** |
| Low | 211 (9.12) | 23 (4.67) | 159 (10.31) | 12 (8.39) | 17 (12.41) |  |
| Moderate | 568 (24.55) | 97 (19.72) | 379 (24.58) | 44 (30.77) | 48 (35.04) |  |
| High | 1535 (66.34) | 372 (75.61) | 1004 (65.11) | 87 (60.84) | 72 (52.55) |  |
| MMSE, n(%) |  |  |  |  |  | 0.116 |
| Normal | 1621 (69.69) | 343 (68.46) | 1091 (70.61) | 103 (72.03) | 84 (61.31) |  |
| Mild cognitive impairment | 705 (30.31) | 158 (31.54) | 454 (29.39) | 40 (27.97) | 53 (38.69) |  |
| MOCA , n(%) |  |  |  |  |  | **0.003** |
| Normal | 781 (33.58) | 187 (37.33) | 522 (33.79) | 44 (30.77) | 28 (20.44) |  |
| Mild cognitive impairment | 1545 (66.42) | 314 (62.67) | 1023 (66.21) | 99 (69.23) | 109 (79.56) |  |
| Hypertension, n(%) |  |  |  |  |  | **<0.001** |
| NO | 817 (35.12) | 269 (53.69) | 417 (26.99) | 82 (57.34) | 49 (35.77) |  |
| YES | 1509 (64.88) | 232 (46.31) | 1128 (73.01) | 61 (42.66) | 88 (64.23) |  |
| Diabetes, n(%) |  |  |  |  |  | **<0.001** |
| NO | 1639 (70.46) | 429 (85.63) | 1004 (64.98) | 119 (83.22) | 87 (63.50) |  |
| YES | 687 (29.54) | 72 (14.37) | 541 (35.02) | 24 (16.78) | 50 (36.50) |  |
| Stroke, n(%) |  |  |  |  |  | **0.009** |
| NO | 1732 (74.46) | 395 (78.84) | 1137 (73.59) | 110 (76.92) | 90 (65.69) |  |
| YES | 594 (25.54) | 106 (21.16) | 408 (26.41) | 33 (23.08) | 47 (34.31) |  |
| Liver disease, n(%) |  |  |  |  |  | **<0.001** |
| NO | 497 (21.37) | 163 (32.53) | 255 (16.50) | 45 (31.47) | 34 (24.82) |  |
| YES | 1829 (78.63) | 338 (67.47) | 1290 (83.50) | 98 (68.53) | 103 (75.18) |  |
| Heart disease, n(%) |  |  |  |  |  | 0.666 |
| NO | 2147 (92.30) | 468 (93.41) | 1419 (91.84) | 132 (92.31) | 128 (93.43) |  |
| YES | 179 (7.70) | 33 (6.59) | 126 (8.16) | 11 (7.69) | 9 (6.57) |  |
| LDL-C, Mean ± SD | 2.92 ± 0.83 | 2.70 ± 0.74 | 3.00 ± 0.84 | 2.63 ± 0.79 | 3.07 ± 0.84 | **<0.001** |
| HDL-C, Mean ± SD | 1.85 ± 0.49 | 2.15 ± 0.48 | 1.71 ± 0.42 | 2.19 ± 0.54 | 1.85 ± 0.50 | **<0.001** |
| TC, Mean ± SD | 5.37 ± 1.11 | 5.32 ± 0.97 | 5.38 ± 1.15 | 5.27 ± 1.10 | 5.56 ± 1.20 | 0.088 |
| ALT, Mean ± SD | 12.21 ± 6.20 | 10.79 ± 5.31 | 12.88 ± 6.47 | 10.92 ± 5.76 | 11.18 ± 5.34 | **<0.001** |
| AST, Mean ± SD | 22.75 ± 6.93 | 22.76 ± 6.24 | 22.78 ± 7.20 | 23.26 ± 7.53 | 21.82 ± 5.28 | 0.356 |
| WBC, Mean ± SD | 6.64 ± 1.79 | 6.14 ± 1.61 | 6.81 ± 1.79 | 6.19 ± 1.66 | 7.09 ± 2.12 | **<0.001** |
| NEU, Mean ± SD | 3.82 ± 1.42 | 3.51 ± 1.36 | 3.91 ± 1.40 | 3.58 ± 1.43 | 4.17 ± 1.60 | **<0.001** |
| LYM, Mean ± SD | 2.21 ± 0.68 | 2.06 ± 0.57 | 2.27 ± 0.70 | 2.00 ± 0.59 | 2.28 ± 0.76 | **<0.001** |
| NLR, Mean ± SD | 1.87 ± 0.88 | 1.83 ± 0.89 | 1.86 ± 0.82 | 1.99 ± 1.27 | 1.98 ± 0.91 | 0.103 |
| PLR, Mean ± SD | 100.23 ± 35.66 | 104.93 ± 37.55 | 97.71 ± 33.42 | 110.31 ± 46.22 | 101.02 ± 37.30 | **<0.001** |
| SII, Mean ± SD | 387.13 ± 222.09 | 375.06 ± 223.28 | 385.38 ± 202.06 | 413.45 ± 365.80 | 423.49 ± 232.55 | 0.064 |
| Abbreviation: N, normal; O, obesity; S, sarcopenia; TyG-WC, triglyceride-glucose-waist circumference; MMSE, mini-mental state examination; MOCA, montreal cognitive assessment; LDL-C, low-density lipoprotein cholesterol; HDL-C, high-density lipoprotein cholesterol; TC, total cholesterol; ALT, alanine aminotransferase; AST, aspartate aminotransferase; WBC, white blood cell; NEU, neutrophil; LYM, lymphocyt; NLR, neutrophil-to-lymphocyte ratio; PLR, platelet-to-lymphocyte ratio; SII, systemic immune-inflammation index. | | | | | | |

**Table S6. Baseline characteristics of participants stratified by sarcopenia and obesity defined by TyG-WHR.**

| **Variables** | **Total (n = 2326)** | **N (n = 529)** | **O (n = 1517)** | **S (n = 105)** | **S+TyG-WHR (n = 175)** | ***P*** |
| --- | --- | --- | --- | --- | --- | --- |
| Age, Mean ± SD | 72.62 ± 5.56 | 71.52 ± 5.09 | 72.42 ± 5.36 | 75.18 ± 5.96 | 76.08 ± 6.55 | **<0.001** |
| Gender, n(%) |  |  |  |  |  | **<0.001** |
| Female | 1301 (55.93) | 340 (64.27) | 807 (53.20) | 60 (57.14) | 94 (53.71) |  |
| Male | 1025 (44.07) | 189 (35.73) | 710 (46.80) | 45 (42.86) | 81 (46.29) |  |
| Education, n(%) |  |  |  |  |  | 0.095 |
| Below high school | 1871 (80.44) | 406 (76.75) | 1225 (80.75) | 88 (83.81) | 152 (86.86) |  |
| High school | 409 (17.58) | 109 (20.60) | 264 (17.40) | 16 (15.24) | 20 (11.43) |  |
| Above high school | 46 (1.98) | 14 (2.65) | 28 (1.85) | 1 (0.95) | 3 (1.71) |  |
| Marital, n(%) |  |  |  |  |  | **<0.001** |
| Married | 1956 (84.09) | 438 (82.80) | 1302 (85.83) | 90 (85.71) | 126 (72.00) |  |
| Others | 370 (15.91) | 91 (17.20) | 215 (14.17) | 15 (14.29) | 49 (28.00) |  |
| Living status, n(%) |  |  |  |  |  | **0.002** |
| Living alone | 328 (14.10) | 81 (15.31) | 199 (13.12) | 11 (10.48) | 37 (21.14) |  |
| Living with spouse and children | 147 (6.32) | 34 (6.43) | 99 (6.53) | 5 (4.76) | 9 (5.14) |  |
| Living with spouse | 1770 (76.10) | 393 (74.29) | 1178 (77.65) | 84 (80.00) | 115 (65.71) |  |
| Living with children | 81 (3.48) | 21 (3.97) | 41 (2.70) | 5 (4.76) | 14 (8.00) |  |
| Smoke, n(%) |  |  |  |  |  | 0.066 |
| Never | 1657 (71.24) | 406 (76.75) | 1054 (69.48) | 71 (67.62) | 126 (72.00) |  |
| Former | 289 (12.42) | 50 (9.45) | 201 (13.25) | 14 (13.33) | 24 (13.71) |  |
| Now | 380 (16.34) | 73 (13.80) | 262 (17.27) | 20 (19.05) | 25 (14.29) |  |
| Drink, n(%) |  |  |  |  |  | **0.005** |
| Never | 1624 (69.82) | 400 (75.61) | 1024 (67.50) | 80 (76.19) | 120 (68.57) |  |
| Former | 186 (8.00) | 31 (5.86) | 126 (8.31) | 9 (8.57) | 20 (11.43) |  |
| Now | 516 (22.18) | 98 (18.53) | 367 (24.19) | 16 (15.24) | 35 (20.00) |  |
| Sleep duration, n(%) |  |  |  |  |  | 0.804 |
| <6 | 503 (21.74) | 122 (23.46) | 317 (20.94) | 27 (25.71) | 37 (21.14) |  |
| 6-8 | 1297 (56.05) | 288 (55.38) | 854 (56.41) | 58 (55.24) | 97 (55.43) |  |
| >8 | 514 (22.21) | 110 (21.15) | 343 (22.66) | 20 (19.05) | 41 (23.43) |  |
| Physical activity, n(%) |  |  |  |  |  | **<0.001** |
| Low | 211 (9.12) | 37 (7.12) | 145 (9.58) | 9 (8.57) | 20 (11.43) |  |
| Moderate | 568 (24.55) | 105 (20.19) | 371 (24.50) | 35 (33.33) | 57 (32.57) |  |
| High | 1535 (66.34) | 378 (72.69) | 998 (65.92) | 61 (58.10) | 98 (56.00) |  |
| MMSE, n(%) |  |  |  |  |  | 0.502 |
| Normal | 1621 (69.69) | 371 (70.13) | 1063 (70.07) | 74 (70.48) | 113 (64.57) |  |
| Mild cognitive impairment | 705 (30.31) | 158 (29.87) | 454 (29.93) | 31 (29.52) | 62 (35.43) |  |
| MOCA , n(%) |  |  |  |  |  | **<0.001** |
| Normal | 781 (33.58) | 202 (38.19) | 507 (33.42) | 37 (35.24) | 35 (20.00) |  |
| Mild cognitive impairment | 1545 (66.42) | 327 (61.81) | 1010 (66.58) | 68 (64.76) | 140 (80.00) |  |
| Hypertension, n(%) |  |  |  |  |  | **<0.001** |
| NO | 817 (35.12) | 275 (51.98) | 411 (27.09) | 61 (58.10) | 70 (40.00) |  |
| YES | 1509 (64.88) | 254 (48.02) | 1106 (72.91) | 44 (41.90) | 105 (60.00) |  |
| Diabetes, n(%) |  |  |  |  |  | **<0.001** |
| NO | 1639 (70.46) | 454 (85.82) | 979 (64.54) | 93 (88.57) | 113 (64.57) |  |
| YES | 687 (29.54) | 75 (14.18) | 538 (35.46) | 12 (11.43) | 62 (35.43) |  |
| Stroke, n(%) |  |  |  |  |  | **0.033** |
| NO | 1732 (74.46) | 414 (78.26) | 1118 (73.70) | 81 (77.14) | 119 (68.00) |  |
| YES | 594 (25.54) | 115 (21.74) | 399 (26.30) | 24 (22.86) | 56 (32.00) |  |
| Liver disease, n(%) |  |  |  |  |  | **<0.001** |
| NO | 497 (21.37) | 170 (32.14) | 248 (16.35) | 27 (25.71) | 52 (29.71) |  |
| YES | 1829 (78.63) | 359 (67.86) | 1269 (83.65) | 78 (74.29) | 123 (70.29) |  |
| Heart disease, n(%) |  |  |  |  |  | 0.466 |
| NO | 2147 (92.30) | 496 (93.76) | 1391 (91.69) | 97 (92.38) | 163 (93.14) |  |
| YES | 179 (7.70) | 33 (6.24) | 126 (8.31) | 8 (7.62) | 12 (6.86) |  |
| LDL-C, Mean ± SD | 2.92 ± 0.83 | 2.68 ± 0.72 | 3.02 ± 0.84 | 2.55 ± 0.75 | 3.03 ± 0.84 | **<0.001** |
| HDL-C, Mean ± SD | 1.85 ± 0.49 | 2.14 ± 0.47 | 1.71 ± 0.42 | 2.21 ± 0.53 | 1.91 ± 0.52 | **<0.001** |
| TC, Mean ± SD | 5.37 ± 1.11 | 5.28 ± 0.98 | 5.40 ± 1.15 | 5.16 ± 1.02 | 5.56 ± 1.21 | **0.004** |
| ALT, Mean ± SD | 12.21 ± 6.20 | 11.21 ± 5.32 | 12.77 ± 6.52 | 11.08 ± 6.14 | 11.03 ± 5.19 | **<0.001** |
| AST, Mean ± SD | 22.75 ± 6.93 | 22.90 ± 6.24 | 22.73 ± 7.22 | 23.84 ± 8.07 | 21.79 ± 5.32 | 0.101 |
| WBC, Mean ± SD | 6.64 ± 1.79 | 6.07 ± 1.53 | 6.85 ± 1.81 | 6.10 ± 1.49 | 6.95 ± 2.12 | **<0.001** |
| NEU, Mean ± SD | 3.82 ± 1.42 | 3.43 ± 1.28 | 3.95 ± 1.41 | 3.51 ± 1.37 | 4.08 ± 1.60 | **<0.001** |
| LYM, Mean ± SD | 2.21 ± 0.68 | 2.07 ± 0.56 | 2.27 ± 0.71 | 1.99 ± 0.57 | 2.23 ± 0.74 | **<0.001** |
| NLR, Mean ± SD | 1.87 ± 0.88 | 1.78 ± 0.84 | 1.87 ± 0.84 | 1.99 ± 1.33 | 1.98 ± 0.95 | **0.017** |
| PLR, Mean ± SD | 100.23 ± 35.66 | 103.63 ± 37.15 | 98.03 ± 33.56 | 109.71 ± 49.38 | 103.39 ± 37.33 | **<0.001** |
| SII, Mean ± SD | 387.13 ± 222.09 | 362.49 ± 215.26 | 389.95 ± 204.25 | 411.27 ± 402.00 | 422.62 ± 234.25 | **0.006** |
| Abbreviation: N, normal; O, obesity; S, sarcopenia; TyG-WHR: triglyceride-glucose-waist-to-hip ratio; MMSE, mini-mental state examination; MOCA, montreal cognitive assessment; LDL-C, low-density lipoprotein cholesterol; HDL-C, high-density lipoprotein cholesterol; TC, total cholesterol; ALT, alanine aminotransferase; AST, aspartate aminotransferase; WBC, white blood cell; NEU, neutrophil; LYM, lymphocyt; NLR, neutrophil-to-lymphocyte ratio; PLR, platelet-to-lymphocyte ratio; SII, systemic immune-inflammation index. | | | | | | |

**Table S7. Baseline characteristics of participants stratified by sarcopenia and obesity defined by TyG-WHtR.**

| **Variables** | **Total (n = 2326)** | **N (n = 640)** | **O (n = 1406)** | **S (n = 138)** | **S+TyG-WHtR (n = 142)** | ***P*** |
| --- | --- | --- | --- | --- | --- | --- |
| Age, Mean ± SD | 72.62 ± 5.56 | 71.51 ± 5.08 | 72.50 ± 5.38 | 74.78 ± 6.02 | 76.68 ± 6.52 | **<0.001** |
| Gender, n(%) |  |  |  |  |  | **<0.001** |
| Female | 1301 (55.93) | 310 (48.44) | 837 (59.53) | 70 (50.72) | 84 (59.15) |  |
| Male | 1025 (44.07) | 330 (51.56) | 569 (40.47) | 68 (49.28) | 58 (40.85) |  |
| Education, n(%) |  |  |  |  |  | **<0.001** |
| Below high school | 1871 (80.44) | 475 (74.22) | 1156 (82.22) | 116 (84.06) | 124 (87.32) |  |
| High school | 409 (17.58) | 146 (22.81) | 227 (16.15) | 20 (14.49) | 16 (11.27) |  |
| Above high school | 46 (1.98) | 19 (2.97) | 23 (1.64) | 2 (1.45) | 2 (1.41) |  |
| Marital, n(%) |  |  |  |  |  | **<0.001** |
| Married | 1956 (84.09) | 548 (85.62) | 1192 (84.78) | 114 (82.61) | 102 (71.83) |  |
| Others | 370 (15.91) | 92 (14.38) | 214 (15.22) | 24 (17.39) | 40 (28.17) |  |
| Living status, n(%) |  |  |  |  |  | **0.008** |
| Living alone | 328 (14.10) | 85 (13.28) | 195 (13.87) | 17 (12.32) | 31 (21.83) |  |
| Living with spouse and children | 147 (6.32) | 41 (6.41) | 92 (6.54) | 7 (5.07) | 7 (4.93) |  |
| Living with spouse | 1770 (76.10) | 494 (77.19) | 1077 (76.60) | 107 (77.54) | 92 (64.79) |  |
| Living with children | 81 (3.48) | 20 (3.12) | 42 (2.99) | 7 (5.07) | 12 (8.45) |  |
| Smoke, n(%) |  |  |  |  |  | 0.070 |
| Never | 1657 (71.24) | 433 (67.66) | 1027 (73.04) | 89 (64.49) | 108 (76.06) |  |
| Former | 289 (12.42) | 91 (14.22) | 160 (11.38) | 21 (15.22) | 17 (11.97) |  |
| Now | 380 (16.34) | 116 (18.12) | 219 (15.58) | 28 (20.29) | 17 (11.97) |  |
| Drink, n(%) |  |  |  |  |  | 0.117 |
| Never | 1624 (69.82) | 425 (66.41) | 999 (71.05) | 95 (68.84) | 105 (73.94) |  |
| Former | 186 (8.00) | 56 (8.75) | 101 (7.18) | 16 (11.59) | 13 (9.15) |  |
| Now | 516 (22.18) | 159 (24.84) | 306 (21.76) | 27 (19.57) | 24 (16.90) |  |
| Sleep duration, n(%) |  |  |  |  |  | 0.054 |
| <6 | 503 (21.74) | 158 (25.16) | 281 (19.99) | 37 (26.81) | 27 (19.01) |  |
| 6-8 | 1297 (56.05) | 349 (55.57) | 793 (56.40) | 75 (54.35) | 80 (56.34) |  |
| >8 | 514 (22.21) | 121 (19.27) | 332 (23.61) | 26 (18.84) | 35 (24.65) |  |
| Physical activity, n(%) |  |  |  |  |  | **<0.001** |
| Low | 211 (9.12) | 33 (5.25) | 149 (10.60) | 11 (7.97) | 18 (12.68) |  |
| Moderate | 568 (24.55) | 140 (22.29) | 336 (23.90) | 45 (32.61) | 47 (33.10) |  |
| High | 1535 (66.34) | 455 (72.45) | 921 (65.50) | 82 (59.42) | 77 (54.23) |  |
| MMSE, n(%) |  |  |  |  |  | 0.435 |
| Normal | 1621 (69.69) | 442 (69.06) | 992 (70.55) | 96 (69.57) | 91 (64.08) |  |
| Mild cognitive impairment | 705 (30.31) | 198 (30.94) | 414 (29.45) | 42 (30.43) | 51 (35.92) |  |
| MOCA , n(%) |  |  |  |  |  | **<0.001** |
| Normal | 781 (33.58) | 252 (39.38) | 457 (32.50) | 47 (34.06) | 25 (17.61) |  |
| Mild cognitive impairment | 1545 (66.42) | 388 (60.62) | 949 (67.50) | 91 (65.94) | 117 (82.39) |  |
| Hypertension, n(%) |  |  |  |  |  | **<0.001** |
| NO | 817 (35.12) | 322 (50.31) | 364 (25.89) | 75 (54.35) | 56 (39.44) |  |
| YES | 1509 (64.88) | 318 (49.69) | 1042 (74.11) | 63 (45.65) | 86 (60.56) |  |
| Diabetes, n(%) |  |  |  |  |  | **<0.001** |
| NO | 1639 (70.46) | 531 (82.97) | 902 (64.15) | 113 (81.88) | 93 (65.49) |  |
| YES | 687 (29.54) | 109 (17.03) | 504 (35.85) | 25 (18.12) | 49 (34.51) |  |
| Stroke, n(%) |  |  |  |  |  | **0.007** |
| NO | 1732 (74.46) | 507 (79.22) | 1025 (72.90) | 103 (74.64) | 97 (68.31) |  |
| YES | 594 (25.54) | 133 (20.78) | 381 (27.10) | 35 (25.36) | 45 (31.69) |  |
| Liver disease, n(%) |  |  |  |  |  | **<0.001** |
| NO | 497 (21.37) | 195 (30.47) | 223 (15.86) | 43 (31.16) | 36 (25.35) |  |
| YES | 1829 (78.63) | 445 (69.53) | 1183 (84.14) | 95 (68.84) | 106 (74.65) |  |
| Heart disease, n(%) |  |  |  |  |  | 0.661 |
| NO | 2147 (92.30) | 597 (93.28) | 1290 (91.75) | 128 (92.75) | 132 (92.96) |  |
| YES | 179 (7.70) | 43 (6.72) | 116 (8.25) | 10 (7.25) | 10 (7.04) |  |
| LDL-C, Mean ± SD | 2.92 ± 0.83 | 2.71 ± 0.75 | 3.03 ± 0.84 | 2.62 ± 0.77 | 3.07 ± 0.85 | **<0.001** |
| HDL-C, Mean ± SD | 1.85 ± 0.49 | 2.08 ± 0.49 | 1.70 ± 0.42 | 2.17 ± 0.55 | 1.89 ± 0.51 | **<0.001** |
| TC, Mean ± SD | 5.37 ± 1.11 | 5.26 ± 0.99 | 5.42 ± 1.15 | 5.22 ± 1.10 | 5.60 ± 1.18 | **<0.001** |
| ALT, Mean ± SD | 12.21 ± 6.20 | 11.24 ± 5.49 | 12.88 ± 6.53 | 11.17 ± 5.94 | 10.92 ± 5.17 | **<0.001** |
| AST, Mean ± SD | 22.75 ± 6.93 | 22.74 ± 6.08 | 22.79 ± 7.36 | 23.36 ± 7.70 | 21.78 ± 5.12 | 0.276 |
| WBC, Mean ± SD | 6.64 ± 1.79 | 6.26 ± 1.60 | 6.82 ± 1.82 | 6.19 ± 1.65 | 7.06 ± 2.12 | **<0.001** |
| NEU, Mean ± SD | 3.82 ± 1.42 | 3.59 ± 1.34 | 3.92 ± 1.41 | 3.56 ± 1.43 | 4.17 ± 1.59 | **<0.001** |
| LYM, Mean ± SD | 2.21 ± 0.68 | 2.07 ± 0.56 | 2.28 ± 0.72 | 2.01 ± 0.57 | 2.26 ± 0.77 | **<0.001** |
| NLR, Mean ± SD | 1.87 ± 0.88 | 1.85 ± 0.86 | 1.85 ± 0.83 | 1.96 ± 1.26 | 2.01 ± 0.94 | 0.117 |
| PLR, Mean ± SD | 100.23 ± 35.66 | 102.53 ± 35.30 | 98.09 ± 34.21 | 108.55 ± 45.47 | 103.05 ± 38.89 | **0.001** |
| SII, Mean ± SD | 387.13 ± 222.09 | 373.36 ± 210.72 | 387.17 ± 205.87 | 406.89 ± 367.42 | 429.52 ± 235.69 | **0.033** |
| Abbreviation: N, normal; O, obesity; S, sarcopenia; TyG-WHtR, triglyceride-glucose-waist-to-height ratio; MMSE, mini-mental state examination; MOCA, montreal cognitive assessment; LDL-C, low-density lipoprotein cholesterol; HDL-C, high-density lipoprotein cholesterol; TC, total cholesterol; ALT, alanine aminotransferase; AST, aspartate aminotransferase; WBC, white blood cell; NEU, neutrophil; LYM, lymphocyt; NLR, neutrophil-to-lymphocyte ratio; PLR, platelet-to-lymphocyte ratio; SII, systemic immune-inflammation index. | | | | | | |

**Table S8. Baseline characteristics of participants stratified by sarcopenia and obesity defined by TyG-ABSI.**

| **Variables** | **Total (n = 2326)** | **N (n = 509)** | **O (n = 1537)** | **S (n = 56)** | **S+TyG-ABSI (n = 224)** | ***P*** |
| --- | --- | --- | --- | --- | --- | --- |
| Age, Mean ± SD | 72.62 ± 5.56 | 71.42 ± 5.17 | 72.44 ± 5.32 | 74.86 ± 5.92 | 75.96 ± 6.43 | **<0.001** |
| Gender, n(%) |  |  |  |  |  | **0.019** |
| Female | 1301 (55.93) | 257 (50.49) | 890 (57.91) | 27 (48.21) | 127 (56.70) |  |
| Male | 1025 (44.07) | 252 (49.51) | 647 (42.09) | 29 (51.79) | 97 (43.30) |  |
| Education, n(%) |  |  |  |  |  | **0.017** |
| Below high school | 1871 (80.44) | 383 (75.25) | 1248 (81.20) | 47 (83.93) | 193 (86.16) |  |
| High school | 409 (17.58) | 113 (22.20) | 260 (16.92) | 9 (16.07) | 27 (12.05) |  |
| Above high school | 46 (1.98) | 13 (2.55) | 29 (1.89) | 0 (0.00) | 4 (1.79) |  |
| Marital, n(%) |  |  |  |  |  | **<0.001** |
| Married | 1956 (84.09) | 432 (84.87) | 1308 (85.10) | 50 (89.29) | 166 (74.11) |  |
| Others | 370 (15.91) | 77 (15.13) | 229 (14.90) | 6 (10.71) | 58 (25.89) |  |
| Living status, n(%) |  |  |  |  |  | **0.005** |
| Living alone | 328 (14.10) | 68 (13.36) | 212 (13.79) | 4 (7.14) | 44 (19.64) |  |
| Living with spouse and children | 147 (6.32) | 35 (6.88) | 98 (6.38) | 2 (3.57) | 12 (5.36) |  |
| Living with spouse | 1770 (76.10) | 389 (76.42) | 1182 (76.90) | 48 (85.71) | 151 (67.41) |  |
| Living with children | 81 (3.48) | 17 (3.34) | 45 (2.93) | 2 (3.57) | 17 (7.59) |  |
| Smoke, n(%) |  |  |  |  |  | 0.803 |
| Never | 1657 (71.24) | 351 (68.96) | 1109 (72.15) | 37 (66.07) | 160 (71.43) |  |
| Former | 289 (12.42) | 68 (13.36) | 183 (11.91) | 8 (14.29) | 30 (13.39) |  |
| Now | 380 (16.34) | 90 (17.68) | 245 (15.94) | 11 (19.64) | 34 (15.18) |  |
| Drink, n(%) |  |  |  |  |  | **0.036** |
| Never | 1624 (69.82) | 333 (65.42) | 1091 (70.98) | 42 (75.00) | 158 (70.54) |  |
| Former | 186 (8.00) | 44 (8.64) | 113 (7.35) | 8 (14.29) | 21 (9.38) |  |
| Now | 516 (22.18) | 132 (25.93) | 333 (21.67) | 6 (10.71) | 45 (20.09) |  |
| Sleep duration, n(%) |  |  |  |  |  | 0.511 |
| <6 | 503 (21.74) | 110 (21.91) | 329 (21.48) | 15 (26.79) | 49 (21.88) |  |
| 6-8 | 1297 (56.05) | 295 (58.76) | 847 (55.29) | 32 (57.14) | 123 (54.91) |  |
| >8 | 514 (22.21) | 97 (19.32) | 356 (23.24) | 9 (16.07) | 52 (23.21) |  |
| Physical activity, n(%) |  |  |  |  |  | **<0.001** |
| Low | 211 (9.12) | 24 (4.78) | 158 (10.31) | 3 (5.36) | 26 (11.61) |  |
| Moderate | 568 (24.55) | 108 (21.51) | 368 (24.02) | 17 (30.36) | 75 (33.48) |  |
| High | 1535 (66.34) | 370 (73.71) | 1006 (65.67) | 36 (64.29) | 123 (54.91) |  |
| MMSE, n(%) |  |  |  |  |  | 0.622 |
| Normal | 1621 (69.69) | 361 (70.92) | 1073 (69.81) | 39 (69.64) | 148 (66.07) |  |
| Mild cognitive impairment | 705 (30.31) | 148 (29.08) | 464 (30.19) | 17 (30.36) | 76 (33.93) |  |
| MOCA , n(%) |  |  |  |  |  | **<0.001** |
| Normal | 781 (33.58) | 194 (38.11) | 515 (33.51) | 21 (37.50) | 51 (22.77) |  |
| Mild cognitive impairment | 1545 (66.42) | 315 (61.89) | 1022 (66.49) | 35 (62.50) | 173 (77.23) |  |
| Hypertension, n(%) |  |  |  |  |  | **<0.001** |
| NO | 817 (35.12) | 235 (46.17) | 451 (29.34) | 31 (55.36) | 100 (44.64) |  |
| YES | 1509 (64.88) | 274 (53.83) | 1086 (70.66) | 25 (44.64) | 124 (55.36) |  |
| Diabetes, n(%) |  |  |  |  |  | **<0.001** |
| NO | 1639 (70.46) | 433 (85.07) | 1000 (65.06) | 50 (89.29) | 156 (69.64) |  |
| YES | 687 (29.54) | 76 (14.93) | 537 (34.94) | 6 (10.71) | 68 (30.36) |  |
| Stroke, n(%) |  |  |  |  |  | 0.127 |
| NO | 1732 (74.46) | 394 (77.41) | 1138 (74.04) | 44 (78.57) | 156 (69.64) |  |
| YES | 594 (25.54) | 115 (22.59) | 399 (25.96) | 12 (21.43) | 68 (30.36) |  |
| Liver disease, n(%) |  |  |  |  |  | **<0.001** |
| NO | 497 (21.37) | 135 (26.52) | 283 (18.41) | 13 (23.21) | 66 (29.46) |  |
| YES | 1829 (78.63) | 374 (73.48) | 1254 (81.59) | 43 (76.79) | 158 (70.54) |  |
| Heart disease, n(%) |  |  |  |  |  | 0.689 |
| NO | 2147 (92.30) | 468 (91.94) | 1419 (92.32) | 50 (89.29) | 210 (93.75) |  |
| YES | 179 (7.70) | 41 (8.06) | 118 (7.68) | 6 (10.71) | 14 (6.25) |  |
| LDL-C, Mean ± SD | 2.92 ± 0.83 | 2.61 ± 0.72 | 3.03 ± 0.83 | 2.49 ± 0.75 | 2.94 ± 0.84 | **<0.001** |
| HDL-C, Mean ± SD | 1.85 ± 0.49 | 2.06 ± 0.48 | 1.74 ± 0.44 | 2.26 ± 0.51 | 1.97 ± 0.54 | **<0.001** |
| TC, Mean ± SD | 5.37 ± 1.11 | 5.12 ± 0.96 | 5.45 ± 1.14 | 5.11 ± 0.99 | 5.49 ± 1.19 | **<0.001** |
| ALT, Mean ± SD | 12.21 ± 6.20 | 11.40 ± 5.53 | 12.69 ± 6.46 | 10.04 ± 4.80 | 11.30 ± 5.71 | **<0.001** |
| AST, Mean ± SD | 22.75 ± 6.93 | 23.02 ± 6.35 | 22.69 ± 7.17 | 23.71 ± 7.17 | 22.27 ± 6.37 | 0.382 |
| WBC, Mean ± SD | 6.64 ± 1.79 | 6.18 ± 1.53 | 6.80 ± 1.82 | 5.87 ± 1.08 | 6.82 ± 2.07 | **<0.001** |
| NEU, Mean ± SD | 3.82 ± 1.42 | 3.54 ± 1.29 | 3.91 ± 1.42 | 3.43 ± 1.03 | 3.98 ± 1.63 | **<0.001** |
| LYM, Mean ± SD | 2.21 ± 0.68 | 2.07 ± 0.57 | 2.27 ± 0.70 | 1.86 ± 0.52 | 2.20 ± 0.71 | **<0.001** |
| NLR, Mean ± SD | 1.87 ± 0.88 | 1.84 ± 0.87 | 1.85 ± 0.83 | 2.08 ± 1.41 | 1.96 ± 1.02 | 0.082 |
| PLR, Mean ± SD | 100.23 ± 35.66 | 102.01 ± 35.62 | 98.64 ± 34.23 | 118.41 ± 58.63 | 102.60 ± 36.58 | **<0.001** |
| SII, Mean ± SD | 387.13 ± 222.09 | 366.80 ± 208.80 | 388.17 ± 206.79 | 438.53 ± 454.64 | 413.33 ± 259.06 | **0.015** |
| Abbreviation: N, normal; O, obesity; S, sarcopenia; TyG-ABSI, triglyceride-glucose-a body shape index; MMSE, mini-mental state examination; MOCA, montreal cognitive assessment; LDL-C, low-density lipoprotein cholesterol; HDL-C, high-density lipoprotein cholesterol; TC, total cholesterol; ALT, alanine aminotransferase; AST, aspartate aminotransferase; WBC, white blood cell; NEU, neutrophil; LYM, lymphocyt; NLR, neutrophil-to-lymphocyte ratio; PLR, platelet-to-lymphocyte ratio; SII, systemic immune-inflammation index. | | | | | | |

**Table S9. Baseline characteristics of participants stratified by sarcopenia and obesity defined by TyG-BRI.**

| **Variables** | **Total (n = 2326)** | **N (n = 677)** | **O (n = 1369)** | **S (n = 142)** | **S+TyG-BRI (n = 138)** | ***P*** |
| --- | --- | --- | --- | --- | --- | --- |
| Age, Mean ± SD | 72.62 ± 5.56 | 71.36 ± 5.03 | 72.59 ± 5.39 | 74.64 ± 5.99 | 76.88 ± 6.50 | **<0.001** |
| Gender, n(%) |  |  |  |  |  | **<0.001** |
| Female | 1301 (55.93) | 329 (48.60) | 818 (59.75) | 70 (49.30) | 84 (60.87) |  |
| Male | 1025 (44.07) | 348 (51.40) | 551 (40.25) | 72 (50.70) | 54 (39.13) |  |
| Education, n(%) |  |  |  |  |  | **<0.001** |
| Below high school | 1871 (80.44) | 493 (72.82) | 1138 (83.13) | 119 (83.80) | 121 (87.68) |  |
| High school | 409 (17.58) | 166 (24.52) | 207 (15.12) | 20 (14.08) | 16 (11.59) |  |
| Above high school | 46 (1.98) | 18 (2.66) | 24 (1.75) | 3 (2.11) | 1 (0.72) |  |
| Marital, n(%) |  |  |  |  |  | **<0.001** |
| Married | 1956 (84.09) | 584 (86.26) | 1156 (84.44) | 118 (83.10) | 98 (71.01) |  |
| Others | 370 (15.91) | 93 (13.74) | 213 (15.56) | 24 (16.90) | 40 (28.99) |  |
| Living status, n(%) |  |  |  |  |  | **0.003** |
| Living alone | 328 (14.10) | 87 (12.85) | 193 (14.10) | 17 (11.97) | 31 (22.46) |  |
| Living with spouse and children | 147 (6.32) | 43 (6.35) | 90 (6.57) | 9 (6.34) | 5 (3.62) |  |
| Living with spouse | 1770 (76.10) | 525 (77.55) | 1046 (76.41) | 109 (76.76) | 90 (65.22) |  |
| Living with children | 81 (3.48) | 22 (3.25) | 40 (2.92) | 7 (4.93) | 12 (8.70) |  |
| Smoke, n(%) |  |  |  |  |  | **0.006** |
| Never | 1657 (71.24) | 456 (67.36) | 1004 (73.34) | 88 (61.97) | 109 (78.99) |  |
| Former | 289 (12.42) | 94 (13.88) | 157 (11.47) | 24 (16.90) | 14 (10.14) |  |
| Now | 380 (16.34) | 127 (18.76) | 208 (15.19) | 30 (21.13) | 15 (10.87) |  |
| Drink, n(%) |  |  |  |  |  | 0.053 |
| Never | 1624 (69.82) | 448 (66.17) | 976 (71.29) | 96 (67.61) | 104 (75.36) |  |
| Former | 186 (8.00) | 57 (8.42) | 100 (7.30) | 17 (11.97) | 12 (8.70) |  |
| Now | 516 (22.18) | 172 (25.41) | 293 (21.40) | 29 (20.42) | 22 (15.94) |  |
| Sleep duration, n(%) |  |  |  |  |  | **0.012** |
| <6 | 503 (21.74) | 166 (24.96) | 273 (19.94) | 39 (27.46) | 25 (18.12) |  |
| 6-8 | 1297 (56.05) | 375 (56.39) | 767 (56.03) | 78 (54.93) | 77 (55.80) |  |
| >8 | 514 (22.21) | 124 (18.65) | 329 (24.03) | 25 (17.61) | 36 (26.09) |  |
| Physical activity, n(%) |  |  |  |  |  | **<0.001** |
| Low | 211 (9.12) | 38 (5.71) | 144 (10.52) | 11 (7.75) | 18 (13.04) |  |
| Moderate | 568 (24.55) | 156 (23.46) | 320 (23.37) | 50 (35.21) | 42 (30.43) |  |
| High | 1535 (66.34) | 471 (70.83) | 905 (66.11) | 81 (57.04) | 78 (56.52) |  |
| MMSE, n(%) |  |  |  |  |  | 0.380 |
| Normal | 1621 (69.69) | 474 (70.01) | 960 (70.12) | 100 (70.42) | 87 (63.04) |  |
| Mild cognitive impairment | 705 (30.31) | 203 (29.99) | 409 (29.88) | 42 (29.58) | 51 (36.96) |  |
| MOCA , n(%) |  |  |  |  |  | **<0.001** |
| Normal | 781 (33.58) | 286 (42.25) | 423 (30.90) | 50 (35.21) | 22 (15.94) |  |
| Mild cognitive impairment | 1545 (66.42) | 391 (57.75) | 946 (69.10) | 92 (64.79) | 116 (84.06) |  |
| Hypertension, n(%) |  |  |  |  |  | **<0.001** |
| NO | 817 (35.12) | 329 (48.60) | 357 (26.08) | 76 (53.52) | 55 (39.86) |  |
| YES | 1509 (64.88) | 348 (51.40) | 1012 (73.92) | 66 (46.48) | 83 (60.14) |  |
| Diabetes, n(%) |  |  |  |  |  | **<0.001** |
| NO | 1639 (70.46) | 531 (78.43) | 902 (65.89) | 111 (78.17) | 95 (68.84) |  |
| YES | 687 (29.54) | 146 (21.57) | 467 (34.11) | 31 (21.83) | 43 (31.16) |  |
| Stroke, n(%) |  |  |  |  |  | **<0.001** |
| NO | 1732 (74.46) | 542 (80.06) | 990 (72.32) | 105 (73.94) | 95 (68.84) |  |
| YES | 594 (25.54) | 135 (19.94) | 379 (27.68) | 37 (26.06) | 43 (31.16) |  |
| Liver disease, n(%) |  |  |  |  |  | **<0.001** |
| NO | 497 (21.37) | 197 (29.10) | 221 (16.14) | 42 (29.58) | 37 (26.81) |  |
| YES | 1829 (78.63) | 480 (70.90) | 1148 (83.86) | 100 (70.42) | 101 (73.19) |  |
| Heart disease, n(%) |  |  |  |  |  | 0.303 |
| NO | 2147 (92.30) | 635 (93.80) | 1252 (91.45) | 132 (92.96) | 128 (92.75) |  |
| YES | 179 (7.70) | 42 (6.20) | 117 (8.55) | 10 (7.04) | 10 (7.25) |  |
| LDL-C, Mean ± SD | 2.92 ± 0.83 | 2.76 ± 0.75 | 3.01 ± 0.85 | 2.66 ± 0.77 | 3.04 ± 0.87 | **<0.001** |
| HDL-C, Mean ± SD | 1.85 ± 0.49 | 2.01 ± 0.50 | 1.73 ± 0.43 | 2.12 ± 0.52 | 1.93 ± 0.56 | **<0.001** |
| TC, Mean ± SD | 5.37 ± 1.11 | 5.28 ± 0.99 | 5.41 ± 1.16 | 5.22 ± 1.04 | 5.61 ± 1.24 | **0.002** |
| ALT, Mean ± SD | 12.21 ± 6.20 | 11.17 ± 5.51 | 12.96 ± 6.53 | 11.21 ± 5.97 | 10.88 ± 5.11 | **<0.001** |
| AST, Mean ± SD | 22.75 ± 6.93 | 22.52 ± 6.18 | 22.90 ± 7.34 | 23.03 ± 7.54 | 22.07 ± 5.33 | 0.404 |
| WBC, Mean ± SD | 6.64 ± 1.79 | 6.29 ± 1.61 | 6.82 ± 1.82 | 6.34 ± 1.78 | 6.93 ± 2.07 | **<0.001** |
| NEU, Mean ± SD | 3.82 ± 1.42 | 3.59 ± 1.33 | 3.92 ± 1.42 | 3.66 ± 1.52 | 4.08 ± 1.54 | **<0.001** |
| LYM, Mean ± SD | 2.21 ± 0.68 | 2.10 ± 0.57 | 2.28 ± 0.72 | 2.03 ± 0.59 | 2.24 ± 0.76 | **<0.001** |
| NLR, Mean ± SD | 1.87 ± 0.88 | 1.83 ± 0.85 | 1.86 ± 0.83 | 1.99 ± 1.28 | 1.98 ± 0.91 | 0.105 |
| PLR, Mean ± SD | 100.23 ± 35.66 | 101.79 ± 34.54 | 98.33 ± 34.59 | 108.15 ± 45.10 | 103.30 ± 39.17 | **0.004** |
| SII, Mean ± SD | 387.13 ± 222.09 | 371.04 ± 206.87 | 388.69 ± 207.56 | 416.30 ± 368.81 | 420.49 ± 228.98 | **0.027** |
| Abbreviation: N, normal; O, obesity; S, sarcopenia; TyG-BRI, triglyceride-glucose- body roundness index; MMSE, mini-mental state examination; MOCA, montreal cognitive assessment; LDL-C, low-density lipoprotein cholesterol; HDL-C, high-density lipoprotein cholesterol; TC, total cholesterol; ALT, alanine aminotransferase; AST, aspartate aminotransferase; WBC, white blood cell; NEU, neutrophil; LYM, lymphocyt; NLR, neutrophil-to-lymphocyte ratio; PLR, platelet-to-lymphocyte ratio; SII, systemic immune-inflammation index. | | | | | | |

**Table S10. Baseline characteristics of participants stratified by sarcopenia and obesity defined by TyG-WWI.**

| **Variables** | **Total (n = 2326)** | **N (n = 763)** | **O (n = 1283)** | **S (n = 107)** | **S+TyG-WWI(n = 173)** | ***P*** |
| --- | --- | --- | --- | --- | --- | --- |
| Age, Mean ± SD | 72.62 ± 5.56 | 71.58 ± 5.18 | 72.55 ± 5.34 | 75.31 ± 6.11 | 76.01 ± 6.48 | **<0.001** |
| Gender, n(%) |  |  |  |  |  | **<0.001** |
| Female | 1301 (55.93) | 331 (43.38) | 816 (63.60) | 46 (42.99) | 108 (62.43) |  |
| Male | 1025 (44.07) | 432 (56.62) | 467 (36.40) | 61 (57.01) | 65 (37.57) |  |
| Education, n(%) |  |  |  |  |  | **<0.001** |
| Below high school | 1871 (80.44) | 573 (75.10) | 1058 (82.46) | 88 (82.24) | 152 (87.86) |  |
| High school | 409 (17.58) | 167 (21.89) | 206 (16.06) | 17 (15.89) | 19 (10.98) |  |
| Above high school | 46 (1.98) | 23 (3.01) | 19 (1.48) | 2 (1.87) | 2 (1.16) |  |
| Marital, n(%) |  |  |  |  |  | **<0.001** |
| Married | 1956 (84.09) | 663 (86.89) | 1077 (83.94) | 91 (85.05) | 125 (72.25) |  |
| Others | 370 (15.91) | 100 (13.11) | 206 (16.06) | 16 (14.95) | 48 (27.75) |  |
| Living status, n(%) |  |  |  |  |  | **0.001** |
| Living alone | 328 (14.10) | 95 (12.45) | 185 (14.42) | 12 (11.21) | 36 (20.81) |  |
| Living with spouse and children | 147 (6.32) | 46 (6.03) | 87 (6.78) | 5 (4.67) | 9 (5.20) |  |
| Living with spouse | 1770 (76.10) | 601 (78.77) | 970 (75.60) | 86 (80.37) | 113 (65.32) |  |
| Living with children | 81 (3.48) | 21 (2.75) | 41 (3.20) | 4 (3.74) | 15 (8.67) |  |
| Smoke, n(%) |  |  |  |  |  | **<0.001** |
| Never | 1657 (71.24) | 500 (65.53) | 960 (74.82) | 65 (60.75) | 132 (76.30) |  |
| Former | 289 (12.42) | 117 (15.33) | 134 (10.44) | 18 (16.82) | 20 (11.56) |  |
| Now | 380 (16.34) | 146 (19.13) | 189 (14.73) | 24 (22.43) | 21 (12.14) |  |
| Drink, n(%) |  |  |  |  |  | **<0.001** |
| Never | 1624 (69.82) | 483 (63.30) | 941 (73.34) | 73 (68.22) | 127 (73.41) |  |
| Former | 186 (8.00) | 69 (9.04) | 88 (6.86) | 15 (14.02) | 14 (8.09) |  |
| Now | 516 (22.18) | 211 (27.65) | 254 (19.80) | 19 (17.76) | 32 (18.50) |  |
| Sleep duration, n(%) |  |  |  |  |  | 0.518 |
| <6 | 503 (21.74) | 159 (21.14) | 280 (21.84) | 26 (24.30) | 38 (21.97) |  |
| 6-8 | 1297 (56.05) | 443 (58.91) | 699 (54.52) | 57 (53.27) | 98 (56.65) |  |
| >8 | 514 (22.21) | 150 (19.95) | 303 (23.63) | 24 (22.43) | 37 (21.39) |  |
| Physical activity, n(%) |  |  |  |  |  | **<0.001** |
| Low | 211 (9.12) | 47 (6.25) | 135 (10.53) | 8 (7.48) | 21 (12.14) |  |
| Moderate | 568 (24.55) | 174 (23.14) | 302 (23.56) | 38 (35.51) | 54 (31.21) |  |
| High | 1535 (66.34) | 531 (70.61) | 845 (65.91) | 61 (57.01) | 98 (56.65) |  |
| MMSE, n(%) |  |  |  |  |  | 0.707 |
| Normal | 1621 (69.69) | 538 (70.51) | 896 (69.84) | 72 (67.29) | 115 (66.47) |  |
| Mild cognitive impairment | 705 (30.31) | 225 (29.49) | 387 (30.16) | 35 (32.71) | 58 (33.53) |  |
| MOCA , n(%) |  |  |  |  |  | **<0.001** |
| Normal | 781 (33.58) | 313 (41.02) | 396 (30.87) | 41 (38.32) | 31 (17.92) |  |
| Mild cognitive impairment | 1545 (66.42) | 450 (58.98) | 887 (69.13) | 66 (61.68) | 142 (82.08) |  |
| Hypertension, n(%) |  |  |  |  |  | **<0.001** |
| NO | 817 (35.12) | 341 (44.69) | 345 (26.89) | 57 (53.27) | 74 (42.77) |  |
| YES | 1509 (64.88) | 422 (55.31) | 938 (73.11) | 50 (46.73) | 99 (57.23) |  |
| Diabetes, n(%) |  |  |  |  |  | **<0.001** |
| NO | 1639 (70.46) | 622 (81.52) | 811 (63.21) | 92 (85.98) | 114 (65.90) |  |
| YES | 687 (29.54) | 141 (18.48) | 472 (36.79) | 15 (14.02) | 59 (34.10) |  |
| Stroke, n(%) |  |  |  |  |  | **0.017** |
| NO | 1732 (74.46) | 599 (78.51) | 933 (72.72) | 78 (72.90) | 122 (70.52) |  |
| YES | 594 (25.54) | 164 (21.49) | 350 (27.28) | 29 (27.10) | 51 (29.48) |  |
| Liver disease, n(%) |  |  |  |  |  | **<0.001** |
| NO | 497 (21.37) | 197 (25.82) | 221 (17.23) | 30 (28.04) | 49 (28.32) |  |
| YES | 1829 (78.63) | 566 (74.18) | 1062 (82.77) | 77 (71.96) | 124 (71.68) |  |
| Heart disease, n(%) |  |  |  |  |  | 0.650 |
| NO | 2147 (92.30) | 707 (92.66) | 1180 (91.97) | 97 (90.65) | 163 (94.22) |  |
| YES | 179 (7.70) | 56 (7.34) | 103 (8.03) | 10 (9.35) | 10 (5.78) |  |
| LDL-C, Mean ± SD | 2.92 ± 0.83 | 2.70 ± 0.74 | 3.07 ± 0.85 | 2.49 ± 0.74 | 3.07 ± 0.83 | **<0.001** |
| HDL-C, Mean ± SD | 1.85 ± 0.49 | 1.99 ± 0.48 | 1.72 ± 0.44 | 2.14 ± 0.55 | 1.95 ± 0.53 | **<0.001** |
| TC, Mean ± SD | 5.37 ± 1.11 | 5.18 ± 0.97 | 5.48 ± 1.16 | 5.05 ± 1.04 | 5.64 ± 1.17 | **<0.001** |
| ALT, Mean ± SD | 12.21 ± 6.20 | 11.58 ± 5.58 | 12.83 ± 6.60 | 10.93 ± 5.47 | 11.12 ± 5.62 | **<0.001** |
| AST, Mean ± SD | 22.75 ± 6.93 | 22.86 ± 6.55 | 22.72 ± 7.23 | 23.44 ± 6.87 | 22.01 ± 6.30 | 0.356 |
| WBC, Mean ± SD | 6.64 ± 1.79 | 6.25 ± 1.52 | 6.88 ± 1.87 | 6.25 ± 1.60 | 6.86 ± 2.11 | **<0.001** |
| NEU, Mean ± SD | 3.82 ± 1.42 | 3.57 ± 1.29 | 3.96 ± 1.44 | 3.62 ± 1.46 | 4.02 ± 1.58 | **<0.001** |
| LYM, Mean ± SD | 2.21 ± 0.68 | 2.09 ± 0.57 | 2.29 ± 0.73 | 2.00 ± 0.59 | 2.22 ± 0.73 | **<0.001** |
| NLR, Mean ± SD | 1.87 ± 0.88 | 1.83 ± 0.86 | 1.86 ± 0.83 | 2.05 ± 1.39 | 1.94 ± 0.89 | 0.064 |
| PLR, Mean ± SD | 100.23 ± 35.66 | 100.48 ± 35.11 | 98.88 ± 34.30 | 109.23 ± 49.10 | 103.62 ± 37.43 | **0.017** |
| SII, Mean ± SD | 387.13 ± 222.09 | 364.71 ± 207.06 | 393.64 ± 207.01 | 424.29 ± 407.38 | 414.70 ± 225.74 | **0.002** |
| Abbreviation: N, normal; O, obesity; S, sarcopenia; TyG-WWI, Triglyceride-glucose- Weight-adjusted waist index; MMSE, mini-mental state examination; MOCA, montreal cognitive assessment; LDL-C, low-density lipoprotein cholesterol; HDL-C, high-density lipoprotein cholesterol; TC, total cholesterol; ALT, alanine aminotransferase; AST, aspartate aminotransferase; WBC, white blood cell; NEU, neutrophil; LYM, lymphocyt; NLR, neutrophil-to-lymphocyte ratio; PLR, platelet-to-lymphocyte ratio; SII, systemic immune-inflammation index. | | | | | | |

**Table S11. Basic characteristics of participants by sarcopenia and obesity defined by TyG-WWI in CHARLS.**

| **Variables** | **Total (n = 3392)** | **N (n = 1271)** | **O (n = 1859)** | **S (n = 168)** | **S+TyG-WWI (n = 94)** | ***P*** |
| --- | --- | --- | --- | --- | --- | --- |
| Age, Mean ± SD | 66.70 ± 5.39 | 66.19 ± 5.02 | 66.55 ± 5.30 | 70.00 ± 6.07 | 70.73 ± 6.78 | **<0.001** |
| Gender, n(%) |  |  |  |  |  | **<0.001** |
| Female | 1377 (40.60) | 259 (20.38) | 1002 (53.90) | 57 (33.93) | 59 (62.77) |  |
| Male | 2015 (59.40) | 1012 (79.62) | 857 (46.10) | 111 (66.07) | 35 (37.23) |  |
| Education, n(%) |  |  |  |  |  | 0.055 |
| Below high school | 3034 (89.45) | 1129 (88.83) | 1659 (89.24) | 157 (93.45) | 89 (94.68) |  |
| High school | 288 (8.49) | 119 (9.36) | 156 (8.39) | 11 (6.55) | 2 (2.13) |  |
| Above high school | 70 (2.06) | 23 (1.81) | 44 (2.37) | 0 (0.00) | 3 (3.19) |  |
| Marital, n(%) |  |  |  |  |  | 0.097 |
| Married | 2817 (83.05) | 1067 (83.95) | 1546 (83.16) | 133 (79.17) | 71 (75.53) |  |
| Others | 575 (16.95) | 204 (16.05) | 313 (16.84) | 35 (20.83) | 23 (24.47) |  |
| Smoke, n(%) |  |  |  |  |  | **<0.001** |
| Never | 1575 (46.45) | 420 (33.07) | 1046 (56.27) | 54 (32.14) | 55 (58.51) |  |
| Former | 731 (21.56) | 303 (23.86) | 377 (20.28) | 34 (20.24) | 17 (18.09) |  |
| Now | 1085 (32.00) | 547 (43.07) | 436 (23.45) | 80 (47.62) | 22 (23.40) |  |
| Drink, n(%) |  |  |  |  |  | **<0.001** |
| Never | 1578 (46.56) | 442 (34.80) | 998 (53.74) | 81 (48.21) | 57 (60.64) |  |
| Former | 474 (13.99) | 191 (15.04) | 242 (13.03) | 32 (19.05) | 9 (9.57) |  |
| Now | 1337 (39.45) | 637 (50.16) | 617 (33.23) | 55 (32.74) | 28 (29.79) |  |
| Sleep duration, n(%) |  |  |  |  |  | **0.023** |
| <6 | 1096 (32.45) | 378 (29.79) | 618 (33.42) | 64 (38.32) | 36 (38.71) |  |
| 6-8 | 2022 (59.86) | 792 (62.41) | 1096 (59.28) | 89 (53.29) | 45 (48.39) |  |
| >8 | 260 (7.70) | 99 (7.80) | 135 (7.30) | 14 (8.38) | 12 (12.90) |  |
| Hypertension, n(%) |  |  |  |  |  | **<0.001** |
| NO | 1901 (59.72) | 818 (68.17) | 906 (52.28) | 118 (71.95) | 59 (68.60) |  |
| YES | 1282 (40.28) | 382 (31.83) | 827 (47.72) | 46 (28.05) | 27 (31.40) |  |
| Diabetes, n(%) |  |  |  |  |  | **<0.001** |
| NO | 2771 (88.11) | 1132 (95.21) | 1411 (82.66) | 154 (93.90) | 74 (87.06) |  |
| YES | 374 (11.89) | 57 (4.79) | 296 (17.34) | 10 (6.10) | 11 (12.94) |  |
| Stroke, n(%) |  |  |  |  |  | 0.113 |
| NO | 3054 (95.95) | 1165 (96.92) | 1649 (95.26) | 156 (95.12) | 84 (97.67) |  |
| YES | 129 (4.05) | 37 (3.08) | 82 (4.74) | 8 (4.88) | 2 (2.33) |  |
| Liver disease, n(%) |  |  |  |  |  | 0.757 |
| NO | 2942 (92.87) | 1109 (92.88) | 1598 (92.64) | 153 (93.87) | 82 (95.35) |  |
| YES | 226 (7.13) | 85 (7.12) | 127 (7.36) | 10 (6.13) | 4 (4.65) |  |
| Heart disease, n(%) |  |  |  |  |  | **<0.001** |
| NO | 2421 (76.57) | 974 (81.44) | 1245 (72.47) | 135 (82.32) | 67 (79.76) |  |
| YES | 741 (23.43) | 222 (18.56) | 473 (27.53) | 29 (17.68) | 17 (20.24) |  |
| LDL, Mean ± SD | 2.67 ± 0.74 | 2.60 ± 0.72 | 2.74 ± 0.76 | 2.47 ± 0.69 | 2.66 ± 0.70 | **<0.001** |
| HDL, Mean ± SD | 1.32 ± 0.31 | 1.40 ± 0.33 | 1.25 ± 0.26 | 1.49 ± 0.33 | 1.35 ± 0.33 | **<0.001** |
| TC, Mean ± SD | 4.77 ± 0.93 | 4.56 ± 0.88 | 4.94 ± 0.94 | 4.52 ± 0.88 | 4.80 ± 0.95 | **<0.001** |
| Mild cognitive impairment, n(%) |  |  |  |  |  | **0.002** |
| NO | 2810 (82.84) | 1071 (84.26) | 1544 (83.06) | 126 (75.00) | 69 (73.40) |  |
| YES | 582 (17.16) | 200 (15.74) | 315 (16.94) | 42 (25.00) | 25 (26.60) |  |
| Abbreviation: N, normal; O, obesity; S, sarcopenia; TyG-WWI, Triglyceride-glucose- Weight-adjusted waist index; LDL-C, low-density lipoprotein cholesterol; HDL-C, high-density lipoprotein cholesterol; TC, total cholesterol. | | | | | | |

**Table S12. Basic characteristics of participants by sarcopenia and obesity defined by TyG-WWI in ELSA.**

| **Variables** | **Total (n = 1825)** | **N (n = 1023)** | **O (n = 670)** | **S (n = 66)** | **S+TyG-WWI (n = 66)** | ***P*** |
| --- | --- | --- | --- | --- | --- | --- |
| Age, Mean ± SD | 67.99 ± 5.55 | 67.63 ± 5.32 | 67.89 ± 5.62 | 71.45 ± 5.95 | 71.11 ± 6.03 | **<0.001** |
| Gender, n(%) |  |  |  |  |  | **<0.001** |
| Female | 1051 (57.59) | 623 (60.90) | 338 (50.45) | 51 (77.27) | 39 (59.09) |  |
| Male | 774 (42.41) | 400 (39.10) | 332 (49.55) | 15 (22.73) | 27 (40.91) |  |
| Education, n(%) |  |  |  |  |  | **<0.001** |
| Below high school | 382 (22.90) | 172 (18.36) | 166 (26.99) | 17 (30.91) | 27 (44.26) |  |
| High school | 909 (54.50) | 523 (55.82) | 327 (53.17) | 27 (49.09) | 32 (52.46) |  |
| Above high school | 377 (22.60) | 242 (25.83) | 122 (19.84) | 11 (20.00) | 2 (3.28) |  |
| Marital, n(%) |  |  |  |  |  | 0.465 |
| Married | 1305 (71.55) | 736 (72.02) | 482 (71.94) | 42 (63.64) | 45 (68.18) |  |
| Others | 519 (28.45) | 286 (27.98) | 188 (28.06) | 24 (36.36) | 21 (31.82) |  |
| Smoke, n(%) |  |  |  |  |  | **0.031** |
| Never | 707 (38.74) | 420 (41.06) | 236 (35.22) | 29 (43.94) | 22 (33.33) |  |
| Former | 973 (53.32) | 539 (52.69) | 366 (54.63) | 31 (46.97) | 37 (56.06) |  |
| Now | 145 (7.95) | 64 (6.26) | 68 (10.15) | 6 (9.09) | 7 (10.61) |  |
| Drink, n(%) |  |  |  |  |  | **<0.001** |
| Never | 142 (8.18) | 52 (5.35) | 69 (10.78) | 10 (16.13) | 11 (17.74) |  |
| Former | 349 (20.10) | 196 (20.16) | 125 (19.53) | 14 (22.58) | 14 (22.58) |  |
| Now | 1245 (71.72) | 724 (74.49) | 446 (69.69) | 38 (61.29) | 37 (59.68) |  |
| Physical activity, n(%) |  |  |  |  |  | **<0.001** |
| Low | 497 (27.25) | 218 (21.31) | 213 (31.84) | 30 (45.45) | 36 (54.55) |  |
| Moderate | 880 (48.25) | 498 (48.68) | 332 (49.63) | 25 (37.88) | 25 (37.88) |  |
| High | 447 (24.51) | 307 (30.01) | 124 (18.54) | 11 (16.67) | 5 (7.58) |  |
| Sleep duration, n(%) |  |  |  |  |  | **<0.001** |
| <6 | 237 (12.99) | 111 (10.86) | 89 (13.28) | 16 (24.24) | 21 (31.82) |  |
| 6-8 | 1457 (79.88) | 850 (83.17) | 519 (77.46) | 47 (71.21) | 41 (62.12) |  |
| >8 | 130 (7.13) | 61 (5.97) | 62 (9.25) | 3 (4.55) | 4 (6.06) |  |
| Hypertension, n(%) |  |  |  |  |  | **<0.001** |
| NO | 1101 (60.33) | 682 (66.67) | 360 (53.73) | 38 (57.58) | 21 (31.82) |  |
| YES | 724 (39.67) | 341 (33.33) | 310 (46.27) | 28 (42.42) | 45 (68.18) |  |
| Diabetes, n(%) |  |  |  |  |  | **<0.001** |
| NO | 1757 (96.27) | 1001 (97.85) | 629 (93.88) | 66 (100.00) | 61 (92.42) |  |
| YES | 68 (3.73) | 22 (2.15) | 41 (6.12) | 0 (0.00) | 5 (7.58) |  |
| Stroke, n(%) |  |  |  |  |  | **0.002** |
| NO | 1781 (97.59) | 996 (97.36) | 662 (98.81) | 62 (93.94) | 61 (92.42) |  |
| YES | 44 (2.41) | 27 (2.64) | 8 (1.19) | 4 (6.06) | 5 (7.58) |  |
| Heart disease, n(%) |  |  |  |  |  | 0.166 |
| NO | 1534 (84.05) | 862 (84.26) | 570 (85.07) | 51 (77.27) | 51 (77.27) |  |
| YES | 291 (15.95) | 161 (15.74) | 100 (14.93) | 15 (22.73) | 15 (22.73) |  |
| LDL, Mean ± SD | 3.32 ± 1.03 | 3.30 ± 1.00 | 3.40 ± 1.07 | 3.16 ± 0.91 | 3.03 ± 1.14 | **0.012** |
| HDL, Mean ± SD | 1.71 ± 0.47 | 1.84 ± 0.46 | 1.50 ± 0.40 | 1.96 ± 0.51 | 1.52 ± 0.40 | **<0.001** |
| TC, Mean ± SD | 5.63 ± 1.16 | 5.61 ± 1.12 | 5.70 ± 1.21 | 5.60 ± 1.01 | 5.29 ± 1.25 | **0.046** |
| Mild cognitive impairment, n(%) |  |  |  |  |  | **0.003** |
| NO | 1598 (87.56) | 919 (89.83) | 569 (84.93) | 58 (87.88) | 52 (78.79) |  |
| YES | 227 (12.44) | 104 (10.17) | 101 (15.07) | 8 (12.12) | 14 (21.21) |  |
| Abbreviation: N, normal; O, obesity; S, sarcopenia; TyG-WWI, Triglyceride-glucose- Weight-adjusted waist index; LDL-C, low-density lipoprotein cholesterol; HDL-C, high-density lipoprotein cholesterol; TC, total cholesterol. | | | | | | |

**Table S13. Logistic regression analysis of the association between SO and MCI defined by MMSE.**

| **Variables** | **Model1** | | **Model2** | | **Model3** | | **Model4** | |
| --- | --- | --- | --- | --- | --- | --- | --- | --- |
|  | **OR (95%CI)** | ***P*** | **OR (95%CI)** | ***P*** | **OR (95%CI)** | ***P*** | **OR (95%CI)** | ***P*** |
| N | Reference |  | Reference |  | Reference |  | Reference |  |
| O | 1.01 (0.79 , 1.29) | 0.952 | 0.99 (0.77 , 1.27) | 0.945 | 1.00 (0.78 , 1.29) | 0.993 | 1.04 (0.80 , 1.36) | 0.765 |
| S | 0.91 (0.53 , 1.56) | 0.732 | 0.92 (0.53 , 1.59) | 0.764 | 0.92 (0.53 , 1.59) | 0.758 | 0.92 (0.53 , 1.60) | 0.764 |
| S+PBF/VFA | 1.29 (0.89 , 1.86) | 0.176 | 1.33 (0.91 , 1.93) | 0.143 | 1.35 (0.92 , 1.97) | 0.126 | 1.36 (0.93 , 1.99) | 0.116 |
| N | Reference |  | Reference |  | Reference |  | Reference |  |
| O | 0.84 (0.68 , 1.04) | 0.102 | 0.83 (0.67 , 1.02) | 0.075 | 0.83 (0.67 , 1.02) | 0.078 | 0.81 (0.64 , 1.03) | 0.088 |
| S | 0.90 (0.63 , 1.29) | 0.565 | 0.91 (0.64 , 1.31) | 0.631 | 0.91 (0.63 , 1.31) | 0.613 | 0.90 (0.63 , 1.30) | 0.578 |
| S+TyG-BMI | 1.33 (0.85 , 2.09) | 0.217 | 1.40 (0.88 , 2.22) | 0.158 | 1.42 (0.89 , 2.27) | 0.139 | 1.37 (0.85 , 2.22) | 0.197 |
| N | Reference |  | Reference |  | Reference |  | Reference |  |
| O | 0.90 (0.73 , 1.12) | 0.361 | 0.89 (0.72 , 1.11) | 0.317 | 0.90 (0.72 , 1.12) | 0.329 | 0.90 (0.70 , 1.16) | 0.422 |
| S | 0.84 (0.56 , 1.27) | 0.415 | 0.87 (0.57 , 1.31) | 0.495 | 0.87 (0.57 , 1.32) | 0.507 | 0.86 (0.56 , 1.31) | 0.478 |
| S+TyG-WC | 1.37 (0.93 , 2.03) | 0.116 | 1.42 (0.95 , 2.13) | 0.090 | 1.43 (0.95 , 2.15) | 0.086 | 1.41 (0.93 , 2.15) | 0.107 |
| N | Reference |  | Reference |  | Reference |  | Reference |  |
| O | 1.00 (0.81 , 1.24) | 0.979 | 0.99 (0.80 , 1.24) | 0.950 | 0.99 (0.80 , 1.24) | 0.943 | 1.01 (0.79 , 1.29) | 0.937 |
| S | 0.98 (0.62 , 1.56) | 0.944 | 1.01 (0.63 , 1.61) | 0.970 | 1.01 (0.63 , 1.62) | 0.957 | 1.01 (0.63 , 1.61) | 0.974 |
| S+TyG-WHR | 1.29 (0.90 , 1.85) | 0.169 | 1.33 (0.92 , 1.93) | 0.131 | 1.33 (0.92 , 1.94) | 0.131 | 1.32 (0.90 , 1.93) | 0.160 |
| N | Reference |  | Reference |  | Reference |  | Reference |  |
| O | 0.93 (0.76 , 1.14) | 0.494 | 0.93 (0.75 , 1.14) | 0.476 | 0.92 (0.75 , 1.14) | 0.455 | 0.93 (0.74 , 1.18) | 0.559 |
| S | 0.98 (0.66 , 1.46) | 0.908 | 1.00 (0.67 , 1.50) | 0.995 | 0.99 (0.66 , 1.49) | 0.970 | 0.99 (0.66 , 1.49) | 0.957 |
| S+TyG-WHtR | 1.25 (0.85 , 1.83) | 0.250 | 1.31 (0.88 , 1.94) | 0.182 | 1.32 (0.89 , 1.96) | 0.174 | 1.30 (0.86 , 1.95) | 0.211 |
| N | Reference |  | Reference |  | Reference |  | Reference |  |
| O | 1.05 (0.85 , 1.31) | 0.635 | 1.06 (0.85 , 1.32) | 0.625 | 1.05 (0.84 , 1.31) | 0.683 | 1.05 (0.82 , 1.33) | 0.717 |
| S | 1.06 (0.58 , 1.94) | 0.841 | 1.08 (0.59 , 1.98) | 0.805 | 1.09 (0.59 , 2.00) | 0.782 | 1.08 (0.59 , 1.99) | 0.804 |
| S+TyG-ABSI | 1.25 (0.89 , 1.75) | 0.189 | 1.31 (0.93 , 1.86) | 0.126 | 1.30 (0.92 , 1.85) | 0.138 | 1.26 (0.89 , 1.81) | 0.196 |
| N | Reference |  | Reference |  | Reference |  | Reference |  |
| O | 0.99 (0.81 , 1.22) | 0.959 | 0.99 (0.81 , 1.22) | 0.957 | 0.99 (0.81 , 1.22) | 0.927 | 1.03 (0.82 , 1.29) | 0.811 |
| S | 0.98 (0.66 , 1.46) | 0.923 | 1.01 (0.68 , 1.52) | 0.945 | 1.01 (0.67 , 1.51) | 0.976 | 1.00 (0.66 , 1.50) | 0.987 |
| S+TyG-BRI | 1.37 (0.93 , 2.01) | 0.108 | 1.43 (0.96 , 2.13) | 0.077 | 1.44 (0.97 , 2.16) | 0.072 | 1.46 (0.97 , 2.19) | 0.067 |
| N | Reference |  | Reference |  | Reference |  | Reference |  |
| O | 1.03 (0.85 , 1.26) | 0.747 | 1.04 (0.85 , 1.27) | 0.701 | 1.02 (0.83 , 1.25) | 0.836 | 1.03 (0.82 , 1.29) | 0.804 |
| S | 1.16 (0.75 , 1.79) | 0.496 | 1.20 (0.77 , 1.86) | 0.418 | 1.19 (0.77 , 1.86) | 0.437 | 1.19 (0.76 , 1.86) | 0.440 |
| S+TyG-WWI | 1.21 (0.85 , 1.71) | 0.297 | 1.27 (0.88 , 1.83) | 0.205 | 1.25 (0.87 , 1.81) | 0.226 | 1.22 (0.84 , 1.77) | 0.302 |
| Note: Model 1 was crude model; Model 2 was adjusted for age, gender, education, marital and living status; Model 3 was adjusted as model 2 plus lifestyle factors (smoking, alcohol consumption, physical activity, and sleep duration). Model 4: was adjusted as model 3 plus LDL-C, HDL-C, TC, hypertension, diabetes, stroke, liver disease, heart disease. Abbreviation: OR, odd ration; CI, confidence interval; N, without sarcopenia and obesity; O, only Obesity; S, only Sarcopenia; S+PBF/VFA, sarcopenia and high body fat percentage or visceral fat area; S+TyG-BMI, sarcopenia and high triglyceride-glucose-body mass index; S+TyG-WC, sarcopenia and high triglyceride-glucose-waist circumference; S+TyG-WHR, sarcopenia and high triglyceride-glucose-waist-to-hip ratio; S+TyG-WHtR, sarcopenia and high triglyceride-glucose-waist-to-height ratio; S+TyG-ABSI, sarcopenia and high triglyceride-glucose-a body shape index; S+TyG-BRI: triglyceride-glucose-body roundness index;S+ TyG-WWI, sarcopenia and high triglyceride-glucose-waist-to-weight index. | | | | | | | | |

**Table S14. Logistic regression analysis of the association between SO and MCI defined by MOCA.**

| **Variables** | **Model1** | | **Model2** | | **Model3** | | **Model4** | |
| --- | --- | --- | --- | --- | --- | --- | --- | --- |
|  | **OR (95%CI)** | ***P*** | **OR (95%CI)** | ***P*** | **OR (95%CI)** | ***P*** | **OR (95%CI)** | ***P*** |
| N | Reference |  | Reference |  | Reference |  | Reference |  |
| O | 1.32 (1.04 , 1.66) | **0.020** | 1.27 (0.99 , 1.63) | 0.060 | 1.25 (0.97 , 1.61) | 0.079 | 1.30 (1.00 , 1.70) | 0.053 |
| S | 1.62 (0.95 , 2.74) | 0.075 | 1.26 (0.72 , 2.21) | 0.412 | 1.34 (0.76 , 2.36) | 0.310 | 1.32 (0.74 , 2.34) | 0.344 |
| S+PBF/VFA | 2.06 (1.40 , 3.02) | **<0.001** | 1.67 (1.11 , 2.51) | **0.015** | 1.66 (1.10 , 2.52) | **0.016** | 1.72 (1.13 , 2.63) | **0.011** |
| N | Reference |  | Reference |  | Reference |  | Reference |  |
| O | 1.16 (0.95 , 1.42) | 0.143 | 1.09 (0.88 , 1.35) | 0.414 | 1.07 (0.86 , 1.33) | 0.530 | 1.10 (0.86 , 1.41) | 0.447 |
| S | 1.43 (1.00 , 2.04) | 0.051 | 1.13 (0.78 , 1.65) | 0.517 | 1.17 (0.80 , 1.71) | 0.424 | 1.18 (0.80 , 1.73) | 0.412 |
| S+TyG-BMI | 2.61 (1.50 , 4.53) | **<0.001** | 2.06 (1.15 , 3.70) | **0.015** | 2.01 (1.11 , 3.63) | **0.021** | 2.06 (1.13 , 3.78) | **0.019** |
| N | Reference |  | Reference |  | Reference |  | Reference |  |
| O | 1.17 (0.95 , 1.44) | 0.148 | 1.10 (0.88 , 1.37) | 0.410 | 1.08 (0.86 , 1.35) | 0.526 | 1.07 (0.83 , 1.38) | 0.621 |
| S | 1.34 (0.90 , 2.00) | 0.150 | 1.09 (0.71 , 1.66) | 0.693 | 1.13 (0.74 , 1.73) | 0.572 | 1.12 (0.73 , 1.73) | 0.592 |
| S+TyG-WC | 2.32 (1.47 , 3.65) | **<0.001** | 1.79 (1.11 , 2.90) | **0.018** | 1.76 (1.08 , 2.86) | **0.024** | 1.73 (1.05 , 2.85) | **0.032** |
| N | Reference |  | Reference |  | Reference |  | Reference |  |
| O | 1.23 (1.01 , 1.51) | **0.048** | 1.19 (0.96 , 1.49) | 0.111 | 1.19 (0.96 , 1.49) | 0.118 | 1.23 (0.96 , 1.58) | 0.107 |
| S | 1.14 (0.73 , 1.76) | 0.569 | 0.92 (0.58 , 1.46) | 0.723 | 0.95 (0.60 , 1.52) | 0.843 | 0.96 (0.60 , 1.53) | 0.855 |
| S+TyG-WHR | 2.47 (1.64 , 3.72) | **<0.001** | 2.02 (1.31 , 3.13) | **0.002** | 2.06 (1.32 , 3.19) | **0.001** | 2.07 (1.32 , 3.26) | **0.002** |
| N | Reference |  | Reference |  | Reference |  | Reference |  |
| O | 1.35 (1.11 , 1.64) | **0.002** | 1.14 (0.93 , 1.40) | 0.215 | 1.10 (0.89 , 1.36) | 0.366 | 1.10 (0.86 , 1.39) | 0.449 |
| S | 1.26 (0.85 , 1.85) | 0.245 | 0.97 (0.64 , 1.46) | 0.878 | 0.98 (0.65 , 1.49) | 0.938 | 0.97 (0.64 , 1.47) | 0.880 |
| S+TyG-WHtR | 3.04 (1.92 , 4.82) | **<0.001** | 2.19 (1.34 , 3.56) | **0.002** | 2.16 (1.32 , 3.53) | **0.002** | 2.14 (1.29 , 3.54) | **0.003** |
| N | Reference |  | Reference |  | Reference |  | Reference |  |
| O | 1.22 (0.99 , 1.50) | 0.059 | 1.07 (0.86 , 1.33) | 0.557 | 1.04 (0.83 , 1.30) | 0.730 | 1.00 (0.78 , 1.27) | 0.984 |
| S | 1.03 (0.58 , 1.81) | 0.928 | 0.79 (0.44 , 1.44) | 0.444 | 0.85 (0.47 , 1.55) | 0.598 | 0.86 (0.47 , 1.59) | 0.633 |
| S+TyG-ABSI | 2.09 (1.46 , 2.99) | **<0.001** | 1.56 (1.06 , 2.28) | **0.024** | 1.54 (1.04 , 2.27) | **0.030** | 1.48 (0.99 , 2.20) | 0.054 |
| N | Reference |  | Reference |  | Reference |  | Reference |  |
| O | 1.64 (1.35 , 1.98) | **<0.001** | 1.36 (1.11 , 1.67) | **0.003** | 1.32 (1.07 , 1.62) | **0.009** | 1.36 (1.09 , 1.71) | **0.007** |
| S | 1.35 (0.92 , 1.96) | 0.122 | 1.05 (0.71 , 1.56) | 0.805 | 1.08 (0.72 , 1.61) | 0.714 | 1.07 (0.71 , 1.61) | 0.742 |
| S+TyG-BRI | 3.86 (2.39 , 6.24) | **<0.001** | 2.70 (1.63 , 4.48) | **<0.001** | 2.64 (1.59 , 4.40) | **<0.001** | 2.66 (1.58 , 4.45) | **<0.001** |
| N | Reference |  | Reference |  | Reference |  | Reference |  |
| O | 1.56 (1.29 , 1.88) | **<0.001** | 1.32 (1.08 , 1.61) | **0.007** | 1.28 (1.04 , 1.56) | **0.019** | 1.29 (1.03 , 1.62) | **0.026** |
| S | 1.12 (0.74 , 1.70) | 0.594 | 0.88 (0.57 , 1.37) | 0.580 | 0.90 (0.58 , 1.41) | 0.647 | 0.87 (0.56 , 1.37) | 0.561 |
| S+TyG-WWI | 3.19 (2.11 , 4.82) | **<0.001** | 2.33 (1.50 , 3.62) | **<0.001** | 2.31 (1.49 , 3.60) | **<0.001** | 2.31 (1.47 , 3.63) | **<0.001** |
| Note: Model 1 was crude model; Model 2 was adjusted for age, gender, education, marital and living status; Model 3 was adjusted as model 2 plus lifestyle factors (smoking, alcohol consumption, physical activity, and sleep duration). Model 4: was adjusted as model 3 plus LDL-C, HDL-C, TC, hypertension, diabetes, stroke, liver disease, heart disease. Abbreviation: OR, odd ration; CI, confidence interval; N, without sarcopenia and obesity; O, only Obesity; S, only Sarcopenia; S+PBF/VFA, sarcopenia and high body fat percentage or visceral fat area; S+TyG-BMI, sarcopenia and high triglyceride-glucose-body mass index; S+TyG-WC, sarcopenia and high triglyceride-glucose-waist circumference; S+TyG-WHR, sarcopenia and high triglyceride-glucose-waist-to-hip ratio; S+TyG-WHtR, sarcopenia and high triglyceride-glucose-waist-to-height ratio; S+TyG-ABSI, sarcopenia and high triglyceride-glucose-a body shape index; S+TyG-BRI: triglyceride-glucose-body roundness index;S+ TyG-WWI, sarcopenia and high triglyceride-glucose-waist-to-weight index. | | | | | | | | |

**Table S15. The ability of obesity indicators in identifying mild cognitive impairment defined by MMSE in older people with sarcopenia.**

|  | **AUC (95%CI)** | **Accuracy (95%CI)** | **Sensitivity (95%CI)** | **Specificity (95%CI)** | **PPV (95%CI)** | **NPV (95%CI)** | **Cut off** | ***P*** |
| --- | --- | --- | --- | --- | --- | --- | --- | --- |
| VFA | 0.55 (0.48-0.63) | 0.57 (0.51-0.63) | 0.58 (0.51 - 0.65) | 0.54 (0.44 - 0.64) | 0.72 (0.65 - 0.79) | 0.39 (0.31 - 0.48) | 78.35 | ref |
| PBF | 0.49 (0.42-0.56) | 0.47 (0.41-0.53) | 0.48 (0.41 - 0.55) | 0.45 (0.35 - 0.55) | 0.64 (0.56 - 0.72) | 0.30 (0.23 - 0.38) | 29.75 | 0.38 |
| TyG-BMI | 0.56 (0.49-0.64) | 0.68 (0.62-0.73) | 0.88 (0.83 - 0.92) | 0.27 (0.18 - 0.36) | 0.71 (0.65 - 0.77) | 0.52 (0.38 - 0.66) | 214.088 | 0.68 |
| TyG-WC | 0.58 (0.51-0.65) | 0.65 (0.59-0.70) | 0.77 (0.71 - 0.83) | 0.40 (0.30 - 0.50) | 0.72 (0.66 - 0.78) | 0.46 (0.35 - 0.57) | 777.13 | 0.36 |
| TYG-WHR | 0.56 (0.48-0.63) | 0.46 (0.40-0.52) | 0.25 (0.19 - 0.31) | 0.88 (0.82 - 0.95) | 0.81 (0.71 - 0.91) | 0.37 (0.31 - 0.43) | 6.938 | 0.96 |
| TyG-WHtR | 0.55 (0.48-0.63) | 0.65 (0.59-0.71) | 0.82 (0.77 - 0.88) | 0.31 (0.22 - 0.41) | 0.71 (0.65 - 0.77) | 0.47 (0.34 - 0.59) | 5.1 | 0.97 |
| TyG-ABSI | 0.55 (0.47-0.62) | 0.62 (0.56-0.68) | 0.73 (0.66 - 0.79) | 0.40 (0.30 - 0.50) | 0.71 (0.64 - 0.77) | 0.42 (0.32 - 0.52) | 7.728 | 0.89 |
| TyG-BRI | 0.56 (0.49-0.63) | 0.46 (0.40-0.52) | 0.27 (0.20 - 0.33) | 0.86 (0.79 - 0.93) | 0.79 (0.69 - 0.89) | 0.37 (0.30 - 0.43) | 26.862 | 0.92 |
| TyG-WWI | 0.54 (0.46-0.61) | 0.55 (0.49-0.61) | 0.57 (0.50 - 0.64) | 0.53 (0.43 - 0.63) | 0.71 (0.63 - 0.78) | 0.38 (0.29 - 0.46) | 99.165 | 0.65 |
| Abbreviation: AUC, area under the curve; PPV, positive predictive value; NPV, negative predictive value; PBF, body fat percentage; VFA, visceral fat area; TyG-BMI, triglyceride-glucose-body mass index; TyG-WC, triglyceride-glucose-waist circumference; TyG-WHR, triglyceride-glucose-waist-to-hip ratio; TyG-WHtR, triglyceride-glucose-waist-to-height ratio; TyG-ABSI, triglyceride-glucose-a body shape index; TyG-BRI: triglyceride-glucose-body roundness index; TyG-WWI, triglyceride-glucose-waist-to-weight index. | | | | | | | | |

**Table S16. The ability of obesity indicators in identifying mild cognitive impairment defined by MOCA in older people with sarcopenia.**

|  | **AUC (95%CI)** | **Accuracy (95%CI)** | **Sensitivity (95%CI)** | **Specificity (95%CI)** | **PPV (95%CI)** | **NPV (95%CI)** | **Cut off** | ***P*** |
| --- | --- | --- | --- | --- | --- | --- | --- | --- |
| VFA | 0.55 (0.47-0.62) | 0.41 (0.36-0.47) | 0.86 (0.78 - 0.94) | 0.26 (0.20 - 0.32) | 0.29 (0.23 - 0.35) | 0.84 (0.75 - 0.93) | 107.75 | ref |
| PBF | 0.54 (0.47-0.62) | 0.38 (0.32-0.44) | 0.94 (0.89 - 1.00) | 0.18 (0.13 - 0.24) | 0.29 (0.23 - 0.34) | 0.90 (0.82 - 0.99) | 37.35 | 0.77 |
| TyG-BMI | 0.54 (0.47-0.62) | 0.42 (0.37-0.49) | 0.89 (0.82 - 0.96) | 0.26 (0.20 - 0.32) | 0.29 (0.23 - 0.36) | 0.87 (0.79 - 0.96) | 209.23 | 0.92 |
| TyG-WC | 0.61 (0.54-0.69) | 0.62 (0.56-0.68) | 0.54 (0.43 - 0.66) | 0.64 (0.58 - 0.71) | 0.35 (0.26 - 0.43) | 0.80 (0.74 - 0.86) | 694.3 | **0.049** |
| TYG-WHR | 0.63 (0.55-0.70) | 0.50 (0.44-0.56) | 0.83 (0.75 - 0.92) | 0.38 (0.32 - 0.45) | 0.32 (0.25 - 0.39) | 0.87 (0.80 - 0.94) | 8.037 | 0.060 |
| TyG-WHtR | 0.64 (0.57-0.71) | 0.56 (0.50-0.62) | 0.79 (0.70 - 0.89) | 0.48 (0.41 - 0.55) | 0.35 (0.27 - 0.42) | 0.87 (0.81 - 0.93) | 4.76 | **0.005** |
| TyG-ABSI | 0.64 (0.56-0.71) | 0.62 (0.57-0.68) | 0.64 (0.53 - 0.75) | 0.62 (0.55 - 0.69) | 0.37 (0.28 - 0.45) | 0.83 (0.77 - 0.89) | 7.279 | **0.04** |
| TyG-BRI | 0.67 (0.60-0.74) | 0.57 (0.51-0.63) | 0.79 (0.70 - 0.89) | 0.49 (0.42 - 0.56) | 0.35 (0.28 - 0.42) | 0.87 (0.81 - 0.93) | 36.901 | **<0.001** |
| TyG-WWI | 0.66 (0.59-0.73) | 0.62 (0.57-0.68) | 0.71 (0.60 - 0.81) | 0.60 (0.53 - 0.66) | 0.38 (0.30 - 0.46) | 0.86 (0.80 - 0.91) | 97.295 | **<0.001** |
| Abbreviation: AUC, area under the curve; PPV, positive predictive value; NPV, negative predictive value; PBF, body fat percentage; VFA, visceral fat area; TyG-BMI, triglyceride-glucose-body mass index; TyG-WC, triglyceride-glucose-waist circumference; TyG-WHR, triglyceride-glucose-waist-to-hip ratio; TyG-WHtR, triglyceride-glucose-waist-to-height ratio; TyG-ABSI, triglyceride-glucose-a body shape index; TyG-BRI: triglyceride-glucose-body roundness index; TyG-WWI, triglyceride-glucose-waist-to-weight index. | | | | | | | | |

**Table S17. The ability of obesity indicators in identifying mild cognitive impairment defined by MOCA in women with sarcopenia.**

|  | **AUC (95%CI)** | **Accuracy (95%CI)** | **Sensitivity (95%CI)** | **Specificity (95%CI)** | **PPV (95%CI)** | **NPV (95%CI)** | **Cut off** | ***P*** |
| --- | --- | --- | --- | --- | --- | --- | --- | --- |
| VFA | 0.54 (0.44-0.63) | 0.47 (0.39-0.55) | 0.84 (0.73 - 0.96) | 0.34 (0.26 - 0.43) | 0.30 (0.21 - 0.38) | 0.87 (0.77 - 0.97) | 107.95 | ref |
| PBF | 0.53 (0.44-0.63) | 0.43 (0.35-0.51) | 0.92 (0.84 - 1.00) | 0.27 (0.19 - 0.35) | 0.29 (0.21 - 0.37) | 0.91 (0.82 - 1.00) | 37.35 | 0.80 |
| TyG-BMI | 0.55 (0.45-0.65) | 0.44 (0.36-0.52) | 0.89 (0.80 - 0.99) | 0.29 (0.21 - 0.38) | 0.29 (0.21 - 0.38) | 0.89 (0.80 - 0.99) | 209.23 | 0.61 |
| TyG-WC | 0.67 (0.57-0.77) | 0.62 (0.54-0.70) | 0.68 (0.54 - 0.83) | 0.60 (0.51 - 0.69) | 0.36 (0.25 - 0.47) | 0.85 (0.78 - 0.93) | 699.592 | **0.001** |
| TYG-WHR | 0.70 (0.60-0.80) | 0.70 (0.62-0.77) | 0.63 (0.48 - 0.78) | 0.72 (0.64 - 0.81) | 0.43 (0.30 - 0.56) | 0.86 (0.79 - 0.93) | 7.285 | **0.002** |
| TyG-WHtR | 0.68 (0.58-0.78) | 0.60 (0.52-0.68) | 0.79 (0.66 - 0.92) | 0.54 (0.45 - 0.63) | 0.36 (0.26 - 0.46) | 0.89 (0.81 - 0.96) | 4.794 | **<0.001** |
| TyG-ABSI | 0.72 (0.62-0.82) | 0.69 (0.62-0.77) | 0.68 (0.54 - 0.83) | 0.70 (0.61 - 0.78) | 0.43 (0.30 - 0.55) | 0.87 (0.80 - 0.94) | 7.279 | **<0.001** |
| TyG-BRI | 0.71 (0.61-0.80) | 0.64 (0.56-0.71) | 0.82 (0.69 - 0.94) | 0.58 (0.49 - 0.67) | 0.39 (0.28 - 0.49) | 0.91 (0.84 - 0.97) | 36.984 | **<0.001** |
| TyG-WWI | 0.73 (0.63-0.83) | 0.70 (0.62-0.77) | 0.68 (0.54 - 0.83) | 0.71 (0.62 - 0.79) | 0.43 (0.31 - 0.56) | 0.87 (0.80 - 0.94) | 97.295 | **<0.001** |
| Abbreviation: AUC, area under the curve; PPV, positive predictive value; NPV, negative predictive value; PBF, body fat percentage; VFA, visceral fat area; TyG-BMI, triglyceride-glucose-body mass index; TyG-WC, triglyceride-glucose-waist circumference; TyG-WHR, triglyceride-glucose-waist-to-hip ratio; TyG-WHtR, triglyceride-glucose-waist-to-height ratio; TyG-ABSI, triglyceride-glucose-a body shape index; TyG-BRI: triglyceride-glucose-body roundness index; TyG-WWI, triglyceride-glucose-waist-to-weight index. | | | | | | | | |

**Table S18. The ability of obesity indicators in identifying mild cognitive impairment defined by MOCA in men with sarcopenia.**

|  | **AUC (95%CI)** | **Accuracy (95%CI)** | **Sensitivity (95%CI)** | **Specificity (95%CI)** | **PPV (95%CI)** | **NPV (95%CI)** | **Cut off** | ***P*** |
| --- | --- | --- | --- | --- | --- | --- | --- | --- |
| VFA | 0.56 (0.45-0.68) | 0.55 (0.46-0.64) | 0.62 (0.45 - 0.78) | 0.52 (0.42 - 0.62) | 0.32 (0.21 - 0.44) | 0.79 (0.68 - 0.89) | 71.7 | ref |
| PBF | 0.56 (0.45-0.68) | 0.66 (0.57-0.74) | 0.53 (0.36 - 0.70) | 0.71 (0.61 - 0.80) | 0.40 (0.26 - 0.54) | 0.80 (0.72 - 0.89) | 23.75 | 0.98 |
| TyG-BMI | 0.53 (0.43-0.64) | 0.40 (0.31-0.49) | 0.91 (0.82 - 1.00) | 0.21 (0.12 - 0.29) | 0.30 (0.21 - 0.39) | 0.86 (0.72 - 1.00) | 211.844 | 0.47 |
| TyG-WC | 0.56 (0.45-0.66) | 0.44 (0.36-0.54) | 0.91 (0.82 - 1.00) | 0.27 (0.18 - 0.36) | 0.32 (0.22 - 0.41) | 0.89 (0.78 - 1.00) | 807.87 | 0.85 |
| TYG-WHR | 0.54 (0.43-0.65) | 0.57 (0.48-0.66) | 0.59 (0.42 - 0.75) | 0.57 (0.46 - 0.67) | 0.33 (0.21 - 0.45) | 0.79 (0.69 - 0.89) | 7.629 | 0.71 |
| TyG-WHtR | 0.58 (0.47-0.69) | 0.52 (0.43-0.61) | 0.79 (0.66 - 0.93) | 0.41 (0.31 - 0.51) | 0.33 (0.23 - 0.44) | 0.84 (0.74 - 0.95) | 4.686 | 0.81 |
| TyG-ABSI | 0.53 (0.42-0.64) | 0.52 (0.43-0.61) | 0.68 (0.52 - 0.83) | 0.47 (0.37 - 0.57) | 0.32 (0.21 - 0.43) | 0.80 (0.69 - 0.90) | 7.411 | 0.58 |
| TyG-BRI | 0.61 (0.50-0.71) | 0.44 (0.36-0.54) | 0.97 (0.91 - 1.00) | 0.25 (0.16 - 0.34) | 0.32 (0.23 - 0.41) | 0.96 (0.88 - 1.00) | 40.976 | 0.39 |
| TyG-WWI | 0.56 (0.45-0.67) | 0.53 (0.44-0.62) | 0.74 (0.59 - 0.88) | 0.46 (0.35 - 0.56) | 0.33 (0.23 - 0.44) | 0.82 (0.72 - 0.93) | 97.288 | 0.98 |
| Abbreviation: AUC, area under the curve; PPV, positive predictive value; NPV, negative predictive value; PBF, body fat percentage; VFA, visceral fat area; TyG-BMI, triglyceride-glucose-body mass index; TyG-WC, triglyceride-glucose-waist circumference; TyG-WHR, triglyceride-glucose-waist-to-hip ratio; TyG-WHtR, triglyceride-glucose-waist-to-height ratio; TyG-ABSI, triglyceride-glucose-a body shape index; TyG-BRI: triglyceride-glucose-body roundness index; TyG-WWI, triglyceride-glucose-waist-to-weight index. | | | | | | | | |

**Table S19. The ability of obesity indicators in identifying mild cognitive impairment defined by MOCA with sarcopenia aged 60-75.**

|  | **AUC (95%CI)** | **Accuracy (95%CI)** | **Sensitivity (95%CI)** | **Specificity (95%CI)** | **PPV (95%CI)** | **NPV (95%CI)** | **Cut off** | ***P*** |
| --- | --- | --- | --- | --- | --- | --- | --- | --- |
| VFA | 0.52 (0.42-0.62) | 0.46 (0.37-0.54) | 0.39 (0.24 - 0.53) | 0.49 (0.39 - 0.59) | 0.27 (0.16 - 0.37) | 0.62 (0.51 - 0.74) | 70.7 | ref |
| PBF | 0.51 (0.41-0.61) | 0.47 (0.38-0.56) | 0.27 (0.14 - 0.40) | 0.57 (0.46 - 0.67) | 0.23 (0.12 - 0.35) | 0.62 (0.52 - 0.72) | 27.7 | 0.80 |
| TyG-BMI | 0.51 (0.41-0.61) | 0.46 (0.38-0.55) | 0.27 (0.14 - 0.40) | 0.55 (0.45 - 0.66) | 0.23 (0.11 - 0.34) | 0.61 (0.51 - 0.72) | 176.772 | 0.91 |
| TyG-WC | 0.58 (0.47-0.68) | 0.51 (0.43-0.60) | 0.75 (0.62 - 0.88) | 0.40 (0.30 - 0.50) | 0.38 (0.27 - 0.48) | 0.77 (0.65 - 0.89) | 744.22 | 0.52 |
| TYG-WHR | 0.60 (0.50-0.70) | 0.51 (0.42-0.59) | 0.89 (0.79 - 0.98) | 0.33 (0.23 - 0.42) | 0.39 (0.29 - 0.48) | 0.86 (0.74 - 0.97) | 8.095 | 0.30 |
| TyG-WHtR | 0.60 (0.50-0.70) | 0.54 (0.45-0.62) | 0.84 (0.73 - 0.95) | 0.39 (0.29 - 0.49) | 0.40 (0.30 - 0.50) | 0.84 (0.73 - 0.95) | 4.794 | 0.35 |
| TyG-ABSI | 0.62 (0.52-0.72) | 0.58 (0.49-0.66) | 0.75 (0.62 - 0.88) | 0.50 (0.40 - 0.60) | 0.42 (0.31 - 0.53) | 0.81 (0.70 - 0.91) | 7.413 | 0.19 |
| TyG-BRI | 0.63 (0.53-0.72) | 0.57 (0.48-0.65) | 0.86 (0.76 - 0.97) | 0.42 (0.32 - 0.52) | 0.42 (0.32 - 0.52) | 0.87 (0.77 - 0.97) | 36.984 | 0.24 |
| TyG-WWI | 0.64 (0.54-0.73) | 0.62 (0.54-0.71) | 0.73 (0.60 - 0.86) | 0.58 (0.48 - 0.68) | 0.45 (0.33 - 0.57) | 0.82 (0.72 - 0.91) | 97.295 | 0.15 |
| Abbreviation: AUC, area under the curve; PPV, positive predictive value; NPV, negative predictive value; PBF, body fat percentage; VFA, visceral fat area; TyG-BMI, triglyceride-glucose-body mass index; TyG-WC, triglyceride-glucose-waist circumference; TyG-WHR, triglyceride-glucose-waist-to-hip ratio; TyG-WHtR, triglyceride-glucose-waist-to-height ratio; TyG-ABSI, triglyceride-glucose-a body shape index; TyG-BRI: triglyceride-glucose-body roundness index; TyG-WWI, triglyceride-glucose-waist-to-weight index. | | | | | | | | |

**Table S20. The ability of obesity indicators in identifying mild cognitive impairment defined by MOCA with sarcopenia aged 75 above.**

|  | **AUC (95%CI)** | **Accuracy (95%CI)** | **Sensitivity (95%CI)** | **Specificity (95%CI)** | **PPV (95%CI)** | **NPV (95%CI)** | **Cut off** | ***P*** |
| --- | --- | --- | --- | --- | --- | --- | --- | --- |
| VFA | 0.62 (0.51-0.75) | 0.75 (0.67-0.82) | 0.43 (0.25 - 0.61) | 0.83 (0.76 - 0.90) | 0.38 (0.21 - 0.54) | 0.86 (0.79 - 0.92) | 23.7 | ref |
| PBF | 0.63 (0.51-0.75) | 0.75 (0.67-0.82) | 0.43 (0.25 - 0.61) | 0.83 (0.76 - 0.90) | 0.38 (0.21 - 0.54) | 0.86 (0.79 - 0.92) | 23.7 | 0.57 |
| TyG-BMI | 0.59 (0.48-0.70) | 0.55 (0.46-0.63) | 0.75 (0.59 - 0.91) | 0.50 (0.41 - 0.59) | 0.27 (0.17 - 0.36) | 0.89 (0.82 - 0.97) | 192.825 | 0.48 |
| TyG-WC | 0.63 (0.52-0.75) | 0.67 (0.59-0.75) | 0.54 (0.35 - 0.72) | 0.71 (0.62 - 0.79) | 0.31 (0.18 - 0.44) | 0.86 (0.79 - 0.93) | 694.432 | 0.76 |
| TYG-WHR | 0.64 (0.52-0.76) | 0.55 (0.46-0.63) | 0.79 (0.63 - 0.94) | 0.49 (0.40 - 0.58) | 0.27 (0.17 - 0.37) | 0.90 (0.83 - 0.98) | 7.846 | 0.68 |
| TyG-WHtR | 0.66 (0.55-0.78) | 0.60 (0.52-0.68) | 0.75 (0.59 - 0.91) | 0.57 (0.48 - 0.66) | 0.30 (0.19 - 0.40) | 0.90 (0.84 - 0.97) | 4.679 | 0.29 |
| TyG-ABSI | 0.64 (0.52-0.75) | 0.67 (0.58-0.74) | 0.61 (0.43 - 0.79) | 0.68 (0.60 - 0.77) | 0.31 (0.19 - 0.44) | 0.88 (0.81 - 0.95) | 7.226 | 0.75 |
| TyG-BRI | 0.69 (0.57-0.80) | 0.72 (0.63-0.79) | 0.57 (0.39 - 0.75) | 0.75 (0.67 - 0.83) | 0.36 (0.22 - 0.50) | 0.88 (0.81 - 0.94) | 30.841 | 0.11 |
| TyG-WWI | 0.67 (0.56-0.79) | 0.69 (0.61-0.77) | 0.61 (0.43 - 0.79) | 0.72 (0.63 - 0.80) | 0.34 (0.21 - 0.47) | 0.88 (0.82 - 0.95) | 94.682 | 0.29 |
| Abbreviation: AUC, area under the curve; PPV, positive predictive value; NPV, negative predictive value; PBF, body fat percentage; VFA, visceral fat area; TyG-BMI, triglyceride-glucose-body mass index; TyG-WC, triglyceride-glucose-waist circumference; TyG-WHR, triglyceride-glucose-waist-to-hip ratio; TyG-WHtR, triglyceride-glucose-waist-to-height ratio; TyG-ABSI, triglyceride-glucose-a body shape index; TyG-BRI: triglyceride-glucose-body roundness index; TyG-WWI, triglyceride-glucose-waist-to-weight index. | | | | | | | | |

**Table S21. The ability of obesity indicators in identifying mild cognitive impairment defined by MMSE or MOCA in older people with sarcopenia.**

|  | **AUC (95%CI)** | **Accuracy (95%CI)** | **Sensitivity (95%CI)** | **Specificity (95%CI)** | **PPV (95%CI)** | **NPV (95%CI)** | **Cut off** | ***P*** |
| --- | --- | --- | --- | --- | --- | --- | --- | --- |
| VFA | 0.56 (0.48-0.64) | 0.39 (0.33-0.45) | 0.88 (0.80 - 0.96) | 0.26 (0.20 - 0.32) | 0.24 (0.18 - 0.30) | 0.89 (0.81 - 0.97) | 107.75 | ref |
| PBF | 0.55 (0.47-0.63) | 0.35 (0.29-0.41) | 0.97 (0.92 - 1.00) | 0.18 (0.13 - 0.23) | 0.24 (0.19 - 0.29) | 0.95 (0.89 - 1.00) | 37.35 | 0.22 |
| TyG-BMI | 0.58 (0.50-0.65) | 0.41 (0.36-0.47) | 0.95 (0.89 - 1.00) | 0.27 (0.21 - 0.33) | 0.26 (0.20 - 0.32) | 0.95 (0.90 - 1.00) | 209.23 | 0.65 |
| TyG-WC | 0.66 (0.58-0.74) | 0.46 (0.40-0.52) | 0.90 (0.82 - 0.98) | 0.35 (0.29 - 0.41) | 0.27 (0.21 - 0.33) | 0.93 (0.87 - 0.98) | 774.743 | **0.007** |
| TYG-WHR | 0.68 (0.60-0.76) | 0.70 (0.64-0.75) | 0.54 (0.42 - 0.67) | 0.74 (0.68 - 0.80) | 0.36 (0.26 - 0.46) | 0.86 (0.81 - 0.91) | 7.278 | **0.01** |
| TyG-WHtR | 0.68 (0.61-0.75) | 0.56 (0.50-0.62) | 0.88 (0.80 - 0.96) | 0.47 (0.40 - 0.54) | 0.31 (0.24 - 0.38) | 0.94 (0.89 - 0.98) | 4.794 | **<0.001** |
| TyG-ABSI | 0.69 (0.62-0.77) | 0.68 (0.62-0.74) | 0.68 (0.56 - 0.80) | 0.68 (0.62 - 0.74) | 0.36 (0.27 - 0.45) | 0.89 (0.84 - 0.94) | 7.132 | **0.007** |
| TyG-BRI | 0.69 (0.62-0.77) | 0.57 (0.51-0.63) | 0.85 (0.76 - 0.94) | 0.49 (0.43 - 0.56) | 0.31 (0.24 - 0.38) | 0.92 (0.88 - 0.97) | 36.841 | **<0.001** |
| TyG-WWI | 0.71 (0.63-0.78) | 0.63 (0.57-0.69) | 0.76 (0.65 - 0.87) | 0.59 (0.53 - 0.66) | 0.33 (0.25 - 0.41) | 0.90 (0.86 - 0.95) | 97.295 | **0.001** |
| Abbreviation: AUC, area under the curve; PPV, positive predictive value; NPV, negative predictive value; PBF, body fat percentage; VFA, visceral fat area; TyG-BMI, triglyceride-glucose-body mass index; TyG-WC, triglyceride-glucose-waist circumference; TyG-WHR, triglyceride-glucose-waist-to-hip ratio; TyG-WHtR, triglyceride-glucose-waist-to-height ratio; TyG-ABSI, triglyceride-glucose-a body shape index; TyG-BRI: triglyceride-glucose-body roundness index; TyG-WWI, triglyceride-glucose-waist-to-weight index. | | | | | | | | |

**Table S22. The ability of obesity indicators in identifying mild cognitive impairment defined by MMSE or MOCA in women with sarcopenia.**

|  | **AUC (95%CI)** | **Accuracy (95%CI)** | **Sensitivity (95%CI)** | **Specificity (95%CI)** | **PPV (95%CI)** | **NPV (95%CI)** | **Cut off** | ***P*** |
| --- | --- | --- | --- | --- | --- | --- | --- | --- |
| VFA | 0.56 (0.45-0.66) | 0.45 (0.37-0.53) | 0.85 (0.73 - 0.97) | 0.34 (0.25 - 0.42) | 0.26 (0.18 - 0.34) | 0.89 (0.80 - 0.98) | 107.95 | ref |
| PBF | 0.54 (0.44-0.64) | 0.41 (0.33-0.49) | 0.94 (0.86 - 1.00) | 0.26 (0.19 - 0.34) | 0.26 (0.18 - 0.34) | 0.94 (0.86 - 1.00) | 37.35 | 0.13 |
| TyG-BMI | 0.56 (0.45-0.67) | 0.42 (0.34-0.50) | 0.91 (0.81 - 1.00) | 0.29 (0.21 - 0.37) | 0.26 (0.18 - 0.34) | 0.92 (0.84 - 1.00) | 209.23 | 0.96 |
| TyG-WC | 0.70 (0.59-0.80) | 0.69 (0.61-0.76) | 0.64 (0.47 - 0.80) | 0.70 (0.62 - 0.78) | 0.37 (0.24 - 0.49) | 0.88 (0.81 - 0.94) | 680.28 | **0.002** |
| TYG-WHR | 0.71 (0.60-0.82) | 0.72 (0.64-0.79) | 0.67 (0.51 - 0.83) | 0.74 (0.66 - 0.81) | 0.41 (0.28 - 0.54) | 0.89 (0.83 - 0.95) | 7.239 | **0.006** |
| TyG-WHtR | 0.70 (0.59-0.80) | 0.60 (0.52-0.68) | 0.82 (0.69 - 0.95) | 0.54 (0.45 - 0.63) | 0.33 (0.22 - 0.43) | 0.92 (0.85 - 0.98) | 4.794 | **0.002** |
| TyG-ABSI | 0.75 (0.65-0.85) | 0.77 (0.70-0.84) | 0.67 (0.51 - 0.83) | 0.80 (0.73 - 0.87) | 0.48 (0.33 - 0.62) | 0.90 (0.84 - 0.96) | 7.036 | **<0.001** |
| TyG-BRI | 0.71 (0.61-0.81) | 0.62 (0.54-0.70) | 0.82 (0.69 - 0.95) | 0.57 (0.48 - 0.66) | 0.34 (0.24 - 0.45) | 0.92 (0.86 - 0.98) | 36.841 | **<0.001** |
| TyG-WWI | 0.74 (0.64-0.85) | 0.77 (0.70-0.84) | 0.61 (0.44 - 0.77) | 0.82 (0.75 - 0.89) | 0.48 (0.33 - 0.63) | 0.88 (0.82 - 0.94) | 93.939 | **<0.001** |
| Abbreviation: AUC, area under the curve; PPV, positive predictive value; NPV, negative predictive value; PBF, body fat percentage; VFA, visceral fat area; TyG-BMI, triglyceride-glucose-body mass index; TyG-WC, triglyceride-glucose-waist circumference; TyG-WHR, triglyceride-glucose-waist-to-hip ratio; TyG-WHtR, triglyceride-glucose-waist-to-height ratio; TyG-ABSI, triglyceride-glucose-a body shape index; TyG-BRI: triglyceride-glucose-body roundness index; TyG-WWI, triglyceride-glucose-waist-to-weight index. | | | | | | | | |

**Table S23. The ability of obesity indicators in identifying mild cognitive impairment defined by MMSE or MOCA in men with sarcopenia.**

|  | **AUC (95%CI)** | **Accuracy (95%CI)** | **Sensitivity (95%CI)** | **Specificity (95%CI)** | **PPV (95%CI)** | **NPV (95%CI)** | **Cut off** | ***P*** |
| --- | --- | --- | --- | --- | --- | --- | --- | --- |
| VFA | 0.59 (0.47-0.72) | 0.55 (0.46-0.64) | 0.65 (0.47 - 0.84) | 0.52 (0.42 - 0.62) | 0.26 (0.15 - 0.37) | 0.85 (0.76 - 0.94) | 71.7 | ref |
| PBF | 0.59 (0.47-0.71) | 0.66 (0.57-0.74) | 0.54 (0.35 - 0.73) | 0.69 (0.60 - 0.78) | 0.31 (0.18 - 0.45) | 0.85 (0.77 - 0.93) | 23.75 | 0.93 |
| TyG-BMI | 0.60 (0.50-0.71) | 0.46 (0.37-0.55) | 0.92 (0.82 - 1.00) | 0.34 (0.25 - 0.43) | 0.27 (0.18 - 0.36) | 0.94 (0.87 - 1.00) | 201.254 | 0.80 |
| TyG-WC | 0.63 (0.52-0.73) | 0.51 (0.42-0.60) | 0.92 (0.82 - 1.00) | 0.40 (0.30 - 0.50) | 0.29 (0.19 - 0.38) | 0.95 (0.89 - 1.00) | 774.743 | 0.48 |
| TYG-WHR | 0.63 (0.53-0.74) | 0.50 (0.41-0.59) | 0.88 (0.76 - 1.00) | 0.40 (0.30 - 0.50) | 0.28 (0.18 - 0.37) | 0.93 (0.85 - 1.00) | 7.999 | 0.50 |
| TyG-WHtR | 0.65 (0.55-0.75) | 0.52 (0.43-0.61) | 0.96 (0.89 - 1.00) | 0.40 (0.30 - 0.50) | 0.29 (0.20 - 0.39) | 0.98 (0.93 - 1.00) | 4.762 | 0.28 |
| TyG-ABSI | 0.61 (0.50-0.72) | 0.60 (0.51-0.69) | 0.69 (0.51 - 0.87) | 0.58 (0.48 - 0.68) | 0.30 (0.18 - 0.42) | 0.88 (0.80 - 0.96) | 7.138 | 0.78 |
| TyG-BRI | 0.66 (0.56-0.77) | 0.53 (0.44-0.62) | 0.85 (0.71 - 0.98) | 0.45 (0.35 - 0.55) | 0.29 (0.18 - 0.39) | 0.92 (0.84 - 1.00) | 35.995 | 0.15 |
| TyG-WWI | 0.65 (0.54-0.75) | 0.55 (0.46-0.64) | 0.85 (0.71 - 0.98) | 0.47 (0.37 - 0.57) | 0.29 (0.19 - 0.40) | 0.92 (0.85 - 1.00) | 97.288 | 0.44 |
| Abbreviation: AUC, area under the curve; PPV, positive predictive value; NPV, negative predictive value; PBF, body fat percentage; VFA, visceral fat area; TyG-BMI, triglyceride-glucose-body mass index; TyG-WC, triglyceride-glucose-waist circumference; TyG-WHR, triglyceride-glucose-waist-to-hip ratio; TyG-WHtR, triglyceride-glucose-waist-to-height ratio; TyG-ABSI, triglyceride-glucose-a body shape index; TyG-BRI: triglyceride-glucose-body roundness index; TyG-WWI, triglyceride-glucose-waist-to-weight index. | | | | | | | | |

**Table S24. The ability of obesity indicators in identifying mild cognitive impairment defined by MMSE or MOCA with sarcopenia aged 60-75.**

|  | **AUC (95%CI)** | **Accuracy (95%CI)** | **Sensitivity (95%CI)** | **Specificity (95%CI)** | **PPV (95%CI)** | **NPV (95%CI)** | **Cut off** | ***P*** |
| --- | --- | --- | --- | --- | --- | --- | --- | --- |
| VFA | 0.49 (0.39-0.59) | 0.48 (0.39-0.57) | 0.41 (0.25 - 0.56) | 0.51 (0.41 - 0.60) | 0.23 (0.13 - 0.34) | 0.69 (0.59 - 0.80) | 70.7 | ref |
| PBF | 0.50 (0.40-0.60) | 0.43 (0.34-0.51) | 1.00 (1.00 - 1.00) | 0.21 (0.13 - 0.29) | 0.32 (0.24 - 0.41) | 1.00 (1.00 - 1.00) | 37.35 | 0.93 |
| TyG-BMI | 0.47 (0.37-0.57) | 0.49 (0.40-0.57) | 0.38 (0.22 - 0.53) | 0.53 (0.43 - 0.62) | 0.23 (0.12 - 0.34) | 0.69 (0.59 - 0.80) | 181.282 | 0.58 |
| TyG-WC | 0.62 (0.52-0.73) | 0.63 (0.55-0.71) | 0.54 (0.38 - 0.70) | 0.67 (0.57 - 0.76) | 0.38 (0.25 - 0.51) | 0.80 (0.71 - 0.88) | 680.28 | 0.15 |
| TYG-WHR | 0.65 (0.55-0.76) | 0.65 (0.56-0.73) | 0.57 (0.41 - 0.73) | 0.68 (0.58 - 0.77) | 0.40 (0.26 - 0.53) | 0.81 (0.72 - 0.89) | 7.255 | 0.05 |
| TyG-WHtR | 0.64 (0.54-0.74) | 0.53 (0.44-0.62) | 0.89 (0.79 - 0.99) | 0.39 (0.30 - 0.49) | 0.35 (0.26 - 0.45) | 0.91 (0.82 - 0.99) | 4.794 | 0.10 |
| TyG-ABSI | 0.67 (0.57-0.77) | 0.64 (0.55-0.72) | 0.70 (0.56 - 0.85) | 0.62 (0.52 - 0.71) | 0.41 (0.29 - 0.53) | 0.85 (0.76 - 0.93) | 7.216 | **0.02** |
| TyG-BRI | 0.65 (0.55-0.75) | 0.55 (0.46-0.64) | 0.89 (0.79 - 0.99) | 0.42 (0.33 - 0.52) | 0.37 (0.27 - 0.47) | 0.91 (0.83 - 0.99) | 36.841 | 0.08 |
| TyG-WWI | 0.68 (0.58-0.78) | 0.62 (0.53-0.70) | 0.76 (0.62 - 0.90) | 0.57 (0.47 - 0.66) | 0.39 (0.28 - 0.51) | 0.86 (0.78 - 0.95) | 97.295 | **0.02** |
| Abbreviation: AUC, area under the curve; PPV, positive predictive value; NPV, negative predictive value; PBF, body fat percentage; VFA, visceral fat area; TyG-BMI, triglyceride-glucose-body mass index; TyG-WC, triglyceride-glucose-waist circumference; TyG-WHR, triglyceride-glucose-waist-to-hip ratio; TyG-WHtR, triglyceride-glucose-waist-to-height ratio; TyG-ABSI, triglyceride-glucose-a body shape index; TyG-BRI: triglyceride-glucose-body roundness index; TyG-WWI, triglyceride-glucose-waist-to-weight index. | | | | | | | | |

**Table S25. The ability of obesity indicators in identifying mild cognitive impairment defined by MMSE or MOCA with sarcopenia aged 75 above.**

|  | **AUC (95%CI)** | **Accuracy (95%CI)** | **Sensitivity (95%CI)** | **Specificity (95%CI)** | **PPV (95%CI)** | **NPV (95%CI)** | **Cut off** | ***P*** |
| --- | --- | --- | --- | --- | --- | --- | --- | --- |
| VFA | 0.63 (0.50-0.76) | 0.58 (0.50-0.66) | 0.68 (0.49 - 0.88) | 0.57 (0.48 - 0.65) | 0.22 (0.12 - 0.32) | 0.91 (0.84 - 0.97) | 75.65 | ref |
| PBF | 0.63 (0.50-0.77) | 0.76 (0.69-0.83) | 0.45 (0.25 - 0.66) | 0.82 (0.75 - 0.89) | 0.31 (0.15 - 0.47) | 0.89 (0.84 - 0.95) | 23.7 | 0.92 |
| TyG-BMI | 0.62 (0.50-0.74) | 0.55 (0.46-0.63) | 0.82 (0.66 - 0.98) | 0.50 (0.41 - 0.59) | 0.23 (0.14 - 0.32) | 0.94 (0.88 - 1.00) | 192.825 | 0.68 |
| TyG-WC | 0.68 (0.56-0.80) | 0.49 (0.41-0.58) | 0.91 (0.79 - 1.00) | 0.42 (0.33 - 0.51) | 0.22 (0.13 - 0.30) | 0.96 (0.91 - 1.00) | 764.901 | 0.27 |
| TYG-WHR | 0.70 (0.58-0.82) | 0.62 (0.54-0.70) | 0.77 (0.60 - 0.95) | 0.60 (0.51 - 0.69) | 0.26 (0.15 - 0.36) | 0.94 (0.88 - 0.99) | 7.634 | 0.22 |
| TyG-WHtR | 0.71 (0.59-0.83) | 0.62 (0.53-0.70) | 0.86 (0.72 - 1.00) | 0.57 (0.49 - 0.66) | 0.27 (0.16 - 0.37) | 0.96 (0.91 - 1.00) | 4.679 | **0.045** |
| TyG-ABSI | 0.71 (0.59-0.82) | 0.72 (0.63-0.79) | 0.68 (0.49 - 0.88) | 0.72 (0.64 - 0.80) | 0.31 (0.18 - 0.44) | 0.93 (0.87 - 0.98) | 7.126 | 0.24 |
| TyG-BRI | 0.72 (0.60-0.84) | 0.73 (0.65-0.80) | 0.64 (0.44 - 0.84) | 0.75 (0.67 - 0.82) | 0.31 (0.18 - 0.45) | 0.92 (0.87 - 0.97) | 30.841 | **0.02** |
| TyG-WWI | 0.73 (0.61-0.85) | 0.56 (0.48-0.64) | 0.91 (0.79 - 1.00) | 0.50 (0.41 - 0.59) | 0.25 (0.15 - 0.34) | 0.97 (0.92 - 1.00) | 102.462 | **0.049** |
| Abbreviation: AUC, area under the curve; PPV, positive predictive value; NPV, negative predictive value; PBF, body fat percentage; VFA, visceral fat area; TyG-BMI, triglyceride-glucose-body mass index; TyG-WC, triglyceride-glucose-waist circumference; TyG-WHR, triglyceride-glucose-waist-to-hip ratio; TyG-WHtR, triglyceride-glucose-waist-to-height ratio; TyG-ABSI, triglyceride-glucose-a body shape index; TyG-BRI: triglyceride-glucose-body roundness index; TyG-WWI, triglyceride-glucose-waist-to-weight index. | | | | | | | | |

**Table S26. Subgroup analysis of the association between sarcopenic obesity (defined by the combination of sarcopenia and TyG-WWI) and mild cognitive impairment defined by MOCA or MMSE.**

| **Variables** | **OR (95%CI)** | ***P*** | ***P* for interaction** |
| --- | --- | --- | --- |
| Age |  |  | 0.229 |
| 60-75 | 2.92 (1.51 , 5.63) | **0.001** |  |
| Above75 | 3.95 (1.48 , 10.58) | **0.006** |  |
| Gender |  |  | 0.852 |
| Female | 3.21 (1.59 , 6.50) | **0.001** |  |
| Male | 3.65 (1.53 , 8.68) | **0.003** |  |
| Education |  |  | 0.835 |
| Below high school | 3.74 (1.95 , 7.17) | **<0.001** |  |
| High school | 2.84 (0.88 , 9.21) | 0.082 |  |
| Above high school | 0 (0.00 , Inf) | 1.000 |  |
| Marital |  |  | 0.066 |
| Married | 2.61 (1.47 , 4.64) | **0.001** |  |
| Others | 19.43 (3.24 , 116.45) | **0.001** |  |
| Smoke |  |  | 0.299 |
| Never | 4.19 (2.16 , 8.14) | **<0.001** |  |
| Former | 3.74 (0.62 , 22.64) | 0.151 |  |
| Now | 1.66 (0.46 , 6.01) | 0.437 |  |
| Drink |  |  | 0.752 |
| Never | 3.88 (2.04 , 7.39) | **<0.001** |  |
| Former | 3.20 (0.32 , 31.70) | 0.320 |  |
| Now | 3.17 (0.81 , 12.41) | 0.098 |  |
| Physical activity |  |  | 0.903 |
| Low | 0.66 (0.02 , 19.47) | 0.809 |  |
| Moderate | 3.86 (1.35 , 11.02) | **0.012** |  |
| High | 3.71 (1.84 , 7.51) | **<0.001** |  |
| Sleep duration |  |  | 0.144 |
| <6 | 2.21 (0.68 , 7.15) | 0.186 |  |
| 6-8 | 3.40 (1.72 , 6.71) | **<0.001** |  |
| >8 | 14.02 (1.59 , 123.87) | **0.018** |  |
| Hypertension |  |  | 0.377 |
| NO | 4.93 (1.90 , 12.76) | **0.001** |  |
| YES | 2.85 (1.43 , 5.69) | **0.003** |  |
| Diabetes |  |  | 0.235 |
| NO | 2.75 (1.49 , 5.06) | **0.001** |  |
| YES | 8.11 (2.28 , 28.79) | **0.001** |  |
| Stroke |  |  | 0.385 |
| NO | 3.20 (1.74 , 5.87) | **<0.001** |  |
| YES | 4.69 (1.35 , 16.29) | **0.015** |  |
| Liver disease |  |  | 0.056 |
| NO | 20.36 (2.52 , 164.49) | **0.005** |  |
| YES | 2.61 (1.45 , 4.69) | **0.001** |  |
| Heart disease |  |  | 0.976 |
| NO | 3.42 (1.99 , 5.89) | **<0.001** |  |
| YES | 0 (0.00 , Inf) | 1 |  |

**Table S27. Association between SO (defined by sarcopenia and TyG-WWI) and cognitive subdomains in the Chongming cohort (assessed by MoCA)**

| **Cognitive subdomains** | **Models** | **Non‐sarcopenia and non-obesity** | **S+TyG-WWI** | |
| --- | --- | --- | --- | --- |
|  |  |  | **β (95% CI)** | ***P*** |
| Visuoconstructional skills | Model 1 | Reference | -0.78 (-0.97 , -0.60) | **<0.001** |
|  | Model 2 | Reference | -0.56 (-0.75 , -0.37) | **<0.001** |
|  | Model 3 | Reference | -0.55 (-0.74 , -0.36) | **<0.001** |
|  | Model 4 | Reference | -0.56 (-0.76 , -0.36) | **<0.001** |
| Executive function | Model 1 | Reference | -0.46 (-0.71 , -0.20) | **<0.001** |
|  | Model 2 | Reference | -0.27 (-0.52 , -0.02) | **0.035** |
|  | Model 3 | Reference | -0.30 (-0.55 , -0.05) | **0.020** |
|  | Model 4 | Reference | -0.28 (-0.54 , -0.02) | **0.036** |
| Naming | Model 1 | Reference | -0.42 (-0.60 , -0.24) | **<0.001** |
|  | Model 2 | Reference | -0.30 (-0.49 , -0.12) | **0.002** |
|  | Model 3 | Reference | -0.33 (-0.51 , -0.14) | **<0.001** |
|  | Model 4 | Reference | -0.32 (-0.51 , -0.13) | **<0.001** |
| Repetition | Model 1 | Reference | -0.16 (-0.30 , -0.03) | **0.019** |
|  | Model 2 | Reference | -0.06 (-0.20 , 0.08) | 0.410 |
|  | Model 3 | Reference | -0.08 (-0.22 , 0.06) | 0.257 |
|  | Model 4 | Reference | -0.10 (-0.24 , 0.04) | 0.169 |
| Recall | Model 1 | Reference | -0.34 (-0.58 , -0.11) | **0.005** |
|  | Model 2 | Reference | -0.19 (-0.43 , 0.05) | 0.116 |
|  | Model 3 | Reference | -0.21 (-0.45 , 0.03) | 0.082 |
|  | Model 4 | Reference | -0.26 (-0.51 , -0.01) | **0.038** |
| Oritention | Model 1 | Reference | -0.08 (-0.18 , 0.03) | 0.162 |
|  | Model 2 | Reference | -0.05 (-0.16 , 0.07) | 0.412 |
|  | Model 3 | Reference | -0.03 (-0.14 , 0.09) | 0.628 |
|  | Model 4 | Reference | -0.02 (-0.14 , 0.10) | 0.727 |
| Caculation | Model 1 | Reference | -0.25 (-0.43 , -0.06) | **0.010** |
|  | Model 2 | Reference | -0.17 (-0.36 , 0.03) | 0.101 |
|  | Model 3 | Reference | -0.15 (-0.35 , 0.04) | 0.128 |
|  | Model 4 | Reference | -0.08 (-0.29 , 0.12) | 0.437 |
| Attention | Model 1 | Reference | -0.05 (-0.10 , 0.00) | 0.064 |
|  | Model 2 | Reference | -0.01 (-0.07 , 0.05) | 0.738 |
|  | Model 3 | Reference | -0.01 (-0.07 , 0.05) | 0.737 |
|  | Model 4 | Reference | -0.01 (-0.07 , 0.05) | 0.820 |
| Note: Model 1 was crude model; Model 2 was adjusted for age, gender, education, marital and living status; Model 3 was adjusted as model 2 plus lifestyle factors (smoking, alcohol consumption, physical activity, and sleep duration). Model 4: was adjusted as model 3 plus LDL-C, HDL-C, TC, hypertension, diabetes, stroke, liver disease, heart disease. Abbreviation: CI, confidence interval; S, Sarcopenia; TyG-WWI, triglyceride-glucose-waist-to-weight index. | | | | |

**Table S28. Association between SO (defined by sarcopenia and TyG-WWI) and cognitive subdomains in the CHARLS cohort (Wave 3, assessed by AACD criteria)**

| **Cognitive subdomains** | **Models** | **Non‐sarcopenia and non-obesity** | **S+TyG-WWI** | |
| --- | --- | --- | --- | --- |
|  |  |  | **β (95% CI)** | ***P*** |
| Memory | Model 1 | Reference | -1.03 (-1.65 , -0.41) | **0.001** |
|  | Model 2 | Reference | -0.69 (-1.30 , -0.08) | **0.027** |
|  | Model 3 | Reference | -0.66 (-1.27 , -0.05) | **0.033** |
|  | Model 4 | Reference | -0.69 (-1.31 , -0.08) | **0.026** |
| Oritention | Model 1 | Reference | -0.16 (-0.35 , 0.03) | 0.090 |
|  | Model 2 | Reference | -0.08 (-0.27 , 0.11) | 0.413 |
|  | Model 3 | Reference | -0.07 (-0.26 , 0.12) | 0.449 |
|  | Model 4 | Reference | -0.08 (-0.27 , 0.11) | 0.390 |
| Draw | Model 1 | Reference | -0.24 (-0.34 , -0.16) | **<0.001** |
|  | Model 2 | Reference | -0.17 (-0.26 , -0.08) | **<0.001** |
|  | Model 3 | Reference | -0.17 (-0.26 , -0.08) | **<0.001** |
|  | Model 4 | Reference | -0.18 (-0.27 , -0.09） | **<0.001** |
| Computation | Model 1 | Reference | -0.41 (-0.65 , -0.16) | **0.001** |
|  | Model 2 | Reference | -0.22 (-0.46 , 0.031) | 0.086 |
|  | Model 3 | Reference | -0.21 (-0.46 , 0.04) | 0.093 |
|  | Model 4 | Reference | -0.23 (-0.48 , 0.02) | 0.067 |
| Note: Model 1 was crude model; Model 2 was adjusted for age, gender, education, marital; Model 3 was adjusted as model 2 plus lifestyle factors (smoking, alcohol consumption and sleep duration). Model 4: was adjusted as model 3 plus LDL-C, HDL-C, TC, hypertension, diabetes, stroke, liver disease, heart disease. Abbreviation: CI, confidence interval; S, Sarcopenia; TyG-WWI, triglyceride-glucose-waist-to-weight index. | | | | |

**Table S29. Association between SO (defined by sarcopenia and TyG-WWI) and cognitive subdomains in the ELSA cohort (Wave 6, assessed by AACD criteria)**

| **Cognitive subdomains** | **Models** | **Non‐sarcopenia and non-obesity** | **S+TyG-WWI** | |
| --- | --- | --- | --- | --- |
|  |  |  | **β (95% CI)** | ***P*** |
| Memory | Model 1 | Reference | -1.88 (-2.50 , -1.25) | **<0.001** |
|  | Model 2 | Reference | -1.06 (-1.64 , -0.47) | **<0.001** |
|  | Model 3 | Reference | -0.93 (-1.52 , -0.34) | **0.002** |
|  | Model 4 | Reference | -0.98 (-1.59 , -0.37) | **0.002** |
| Oritention | Model 1 | Reference | -0.09 (-0.18 , 0.00) | 0.059 |
|  | Model 2 | Reference | -0.06 (-0.16 , 0.03) | 0.173 |
|  | Model 3 | Reference | -0.04 (-0.14 , 0.05) | 0.370 |
|  | Model 4 | Reference | -0.06 (-0.15 , 0.04) | 0.231 |
| Note: Model 1 was crude model; Model 2 was adjusted for age, gender, education, marital; Model 3 was adjusted as model 2 plus lifestyle factors (smoking, alcohol consumption, physical activity and sleep duration). Model 4: was adjusted as model 3 plus LDL-C, HDL-C, TC, hypertension, diabetes, stroke, heart disease. Abbreviation: CI, confidence interval; S, Sarcopenia; TyG-WWI, triglyceride-glucose-waist-to-weight index. | | | | |

**Table S30. The mediating effect of inflammatory markers on the association between sarcopenic obesity (defined by the combination of sarcopenia and TyG-WWI) and mild cognitive impairment defined by MOCA or MMSE.**

|  | **Effect** | **β (95%CI)** | ***P*** | **Mediation** |
| --- | --- | --- | --- | --- |
| ALT | Indirect | 0.00 (-0.00 ~ 0.00) | 0.740 | 0.12 |
|  | Direct | 0.20 (0.13 ~ 0.26) | **<0.001** | 99.88 |
|  | Total | 0.20 (0.13 ~ 0.26) | **<0.001** | 100.00 |
| AST | Indirect | -0.00 (-0.01 ~ 0.00) | 0.620 | -0.26 |
|  | Direct | 0.20 (0.13 ~ 0.26) | **<0.001** | 100.26 |
|  | Total | 0.20 (0.13 ~ 0.26) | **<0.001** | 100.00 |
| WBC | Indirect | 0.00 (-0.01 ~ 0.01) | 0.520 | 1.21 |
|  | Direct | 0.20 (0.12 ~ 0.26) | **<0.001** | 98.79 |
|  | Total | 0.20 (0.13 ~ 0.26) | **<0.001** | 100.00 |
| NEU | Indirect | 0.00 (-0.00 ~ 0.01) | 0.440 | 1.81 |
|  | Direct | 0.19 (0.12 ~ 0.26) | **<0.001** | 98.19 |
|  | Total | 0.20 (0.13 ~ 0.26) | **<0.001** | 100.00 |
| LYM | Indirect | -0.00 (-0.00 ~ 0.00) | 0.800 | -0.10 |
|  | Direct | 0.20 (0.13 ~ 0.26) | **<0.001** | 100.10 |
|  | Total | 0.20 (0.13 ~ 0.26) | **<0.001** | 100.00 |
| NLR | Indirect | 0.00 (-0.00 ~ 0.01) | 0.580 | 0.61 |
|  | Direct | 0.20 (0.12 ~ 0.26) | **<0.001** | 99.39 |
|  | Total | 0.20 (0.13 ~ 0.26) | **<0.001** | 100.00 |
| PLR | Indirect | 0.00 (-0.00 ~ 0.00) | 0.900 | 0.02 |
|  | Direct | 0.20 (0.13 ~ 0.26) | **<0.001** | 99.98 |
|  | Total | 0.20 (0.13 ~ 0.26) | **<0.001** | 100.00 |
| SII | Indirect | 0.00 (-0.01 ~ 0.01) | 0.800 | 0.35 |
|  | Direct | 0.20 (0.13 ~ 0.26) | **<0.001** | 99.65 |
|  | Total | 0.20 (0.12 ~ 0.26) | **<0.001** | 100.00 |
| Abbreviations: ALT, alanine aminotransferase; AST, aspartate aminotransferase; WBC, white blood cell; NEU, neutrophil; LYM, lymphocyt; NLR, neutrophil-to-lymphocyte ratio; PLR, platelet-to-lymphocyte ratio; SII, systemic immune-inflammation index. | | | | |

| 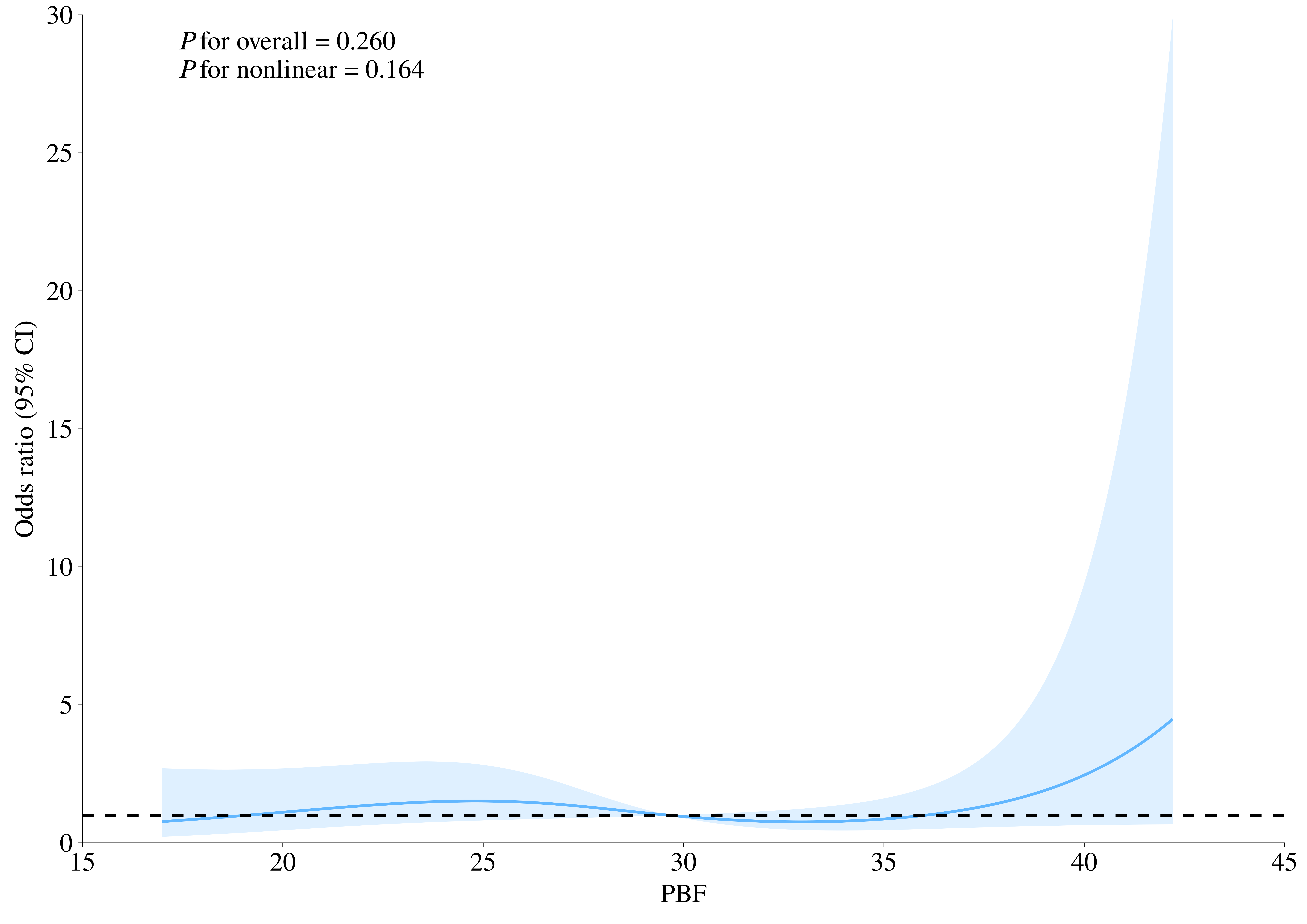 | 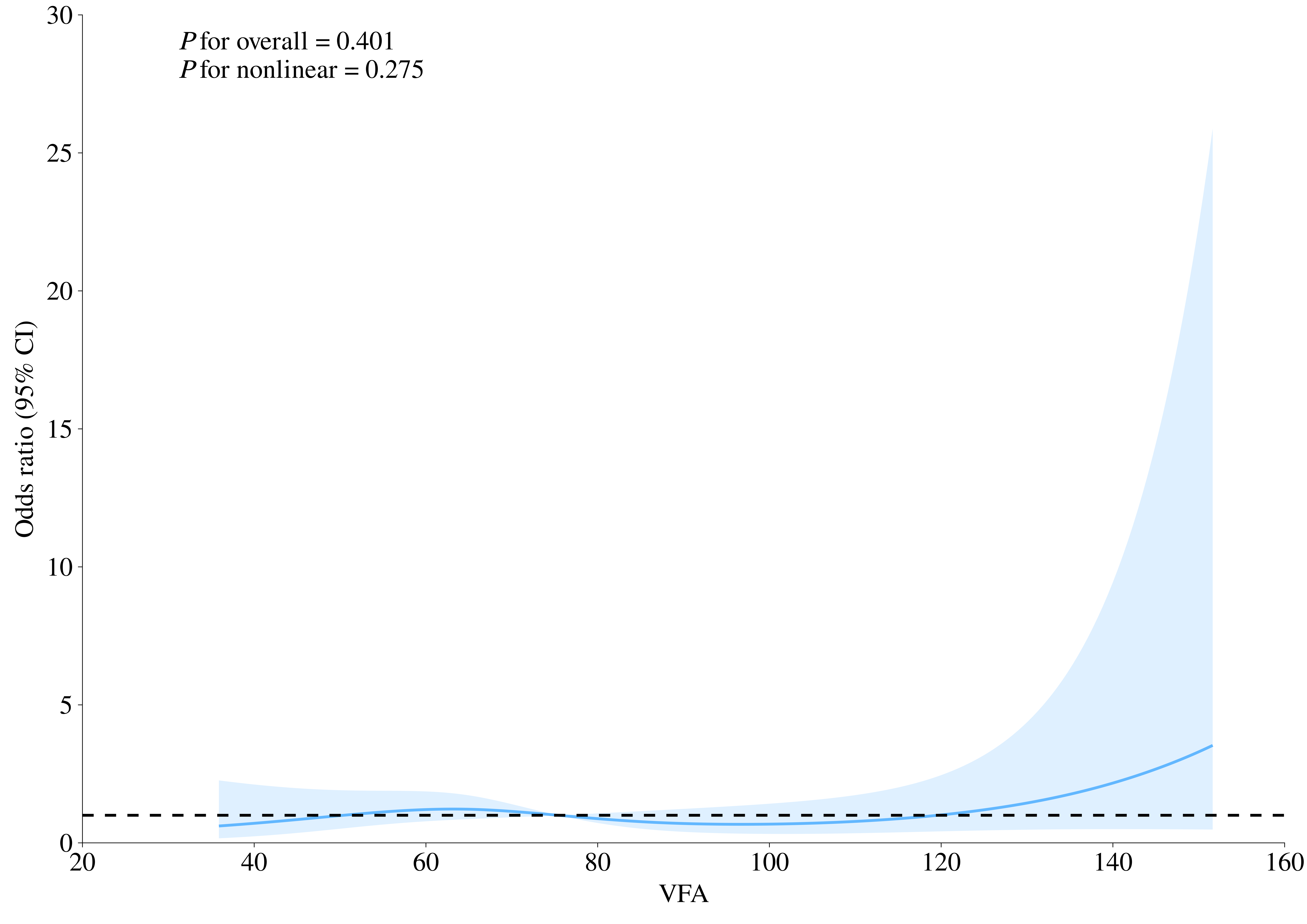 |
| --- | --- |
| 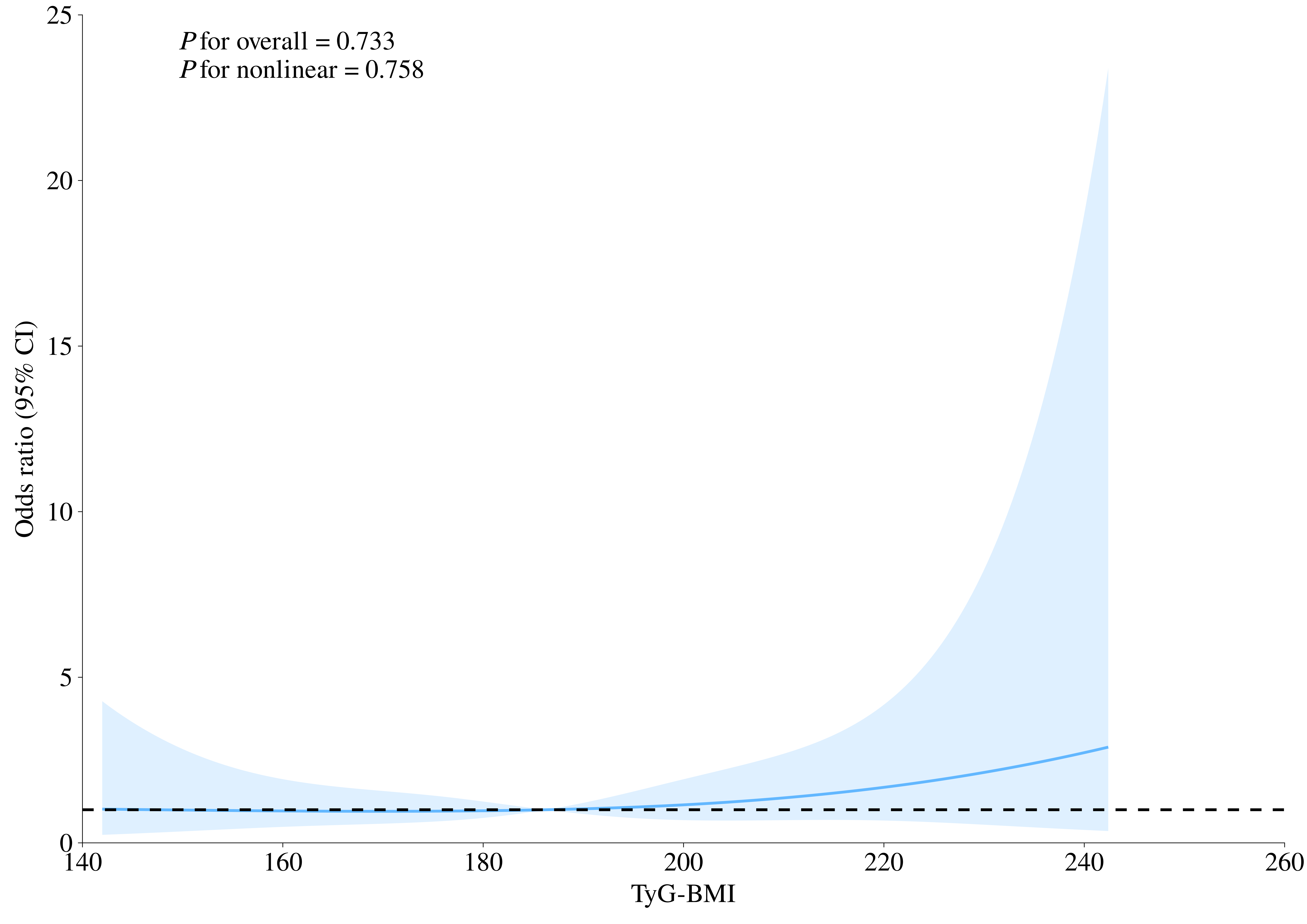 |  |

**Figure S1. RCS of association between obesity indices and MCI defined by MOCA or MMSE in sarcopenia.** Abbreviation: OR, odd ration; PBF, body fat percentage; VFA, visceral fat area; TyG-BMI, triglyceride-glucose-body mass index.


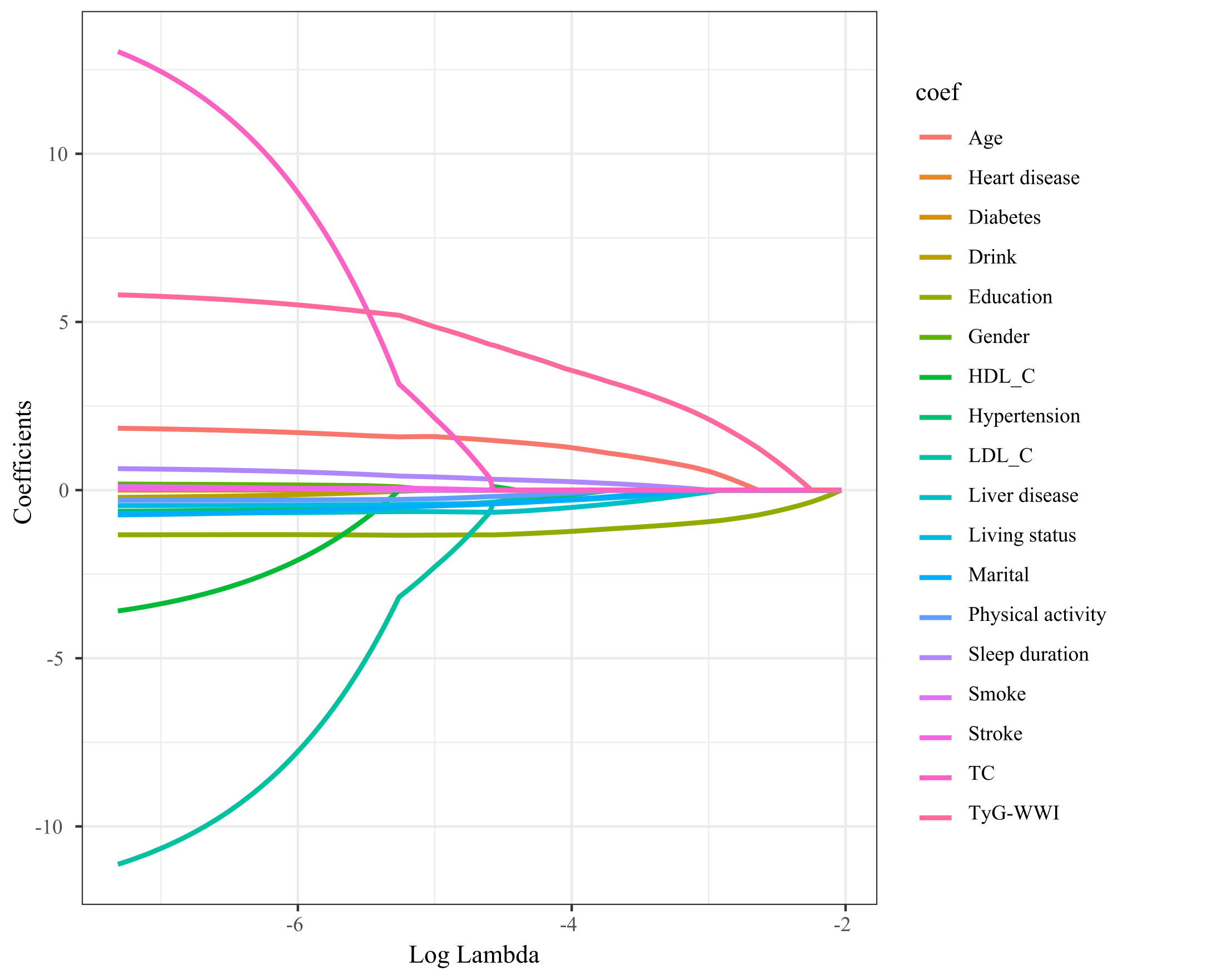


**Figure S2. LASSO regression coefficient path plot.** Abbreviation: LDL-C, low-density lipoprotein cholesterol; HDL-C, high-density lipoprotein cholesterol; TC, total cholesterol; TyG-WWI, triglyceride-glucose index-waist-to-weight index.


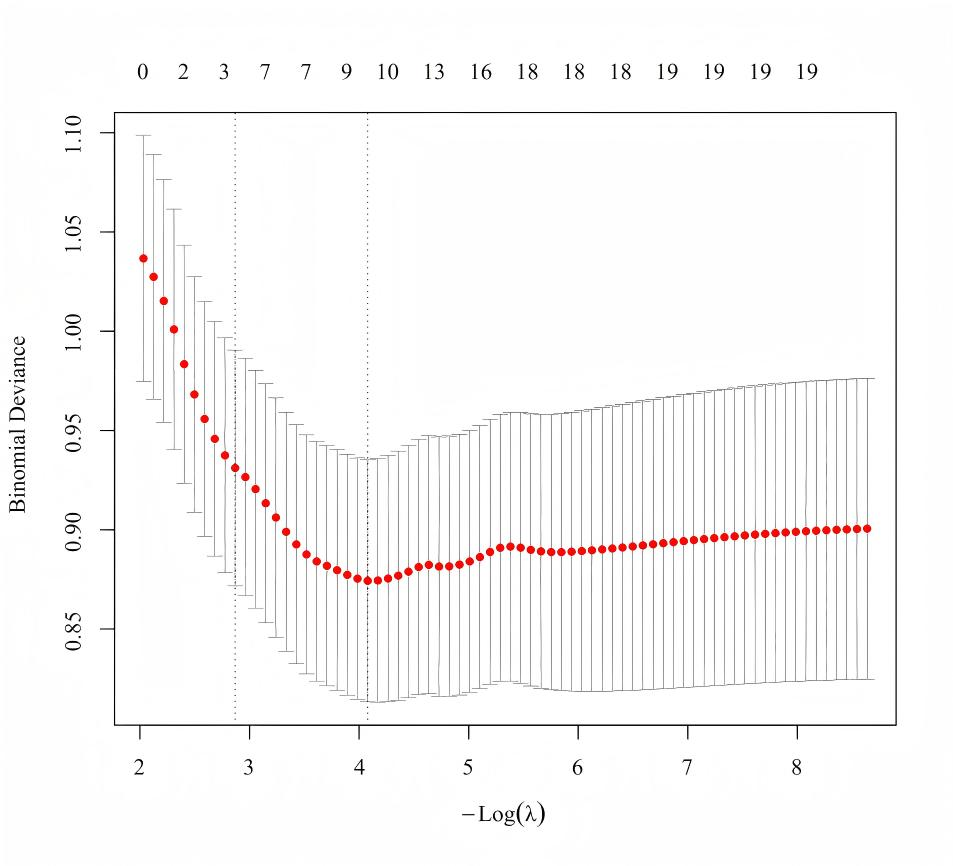


**Figure S3. LASSO regression cross validation results.**


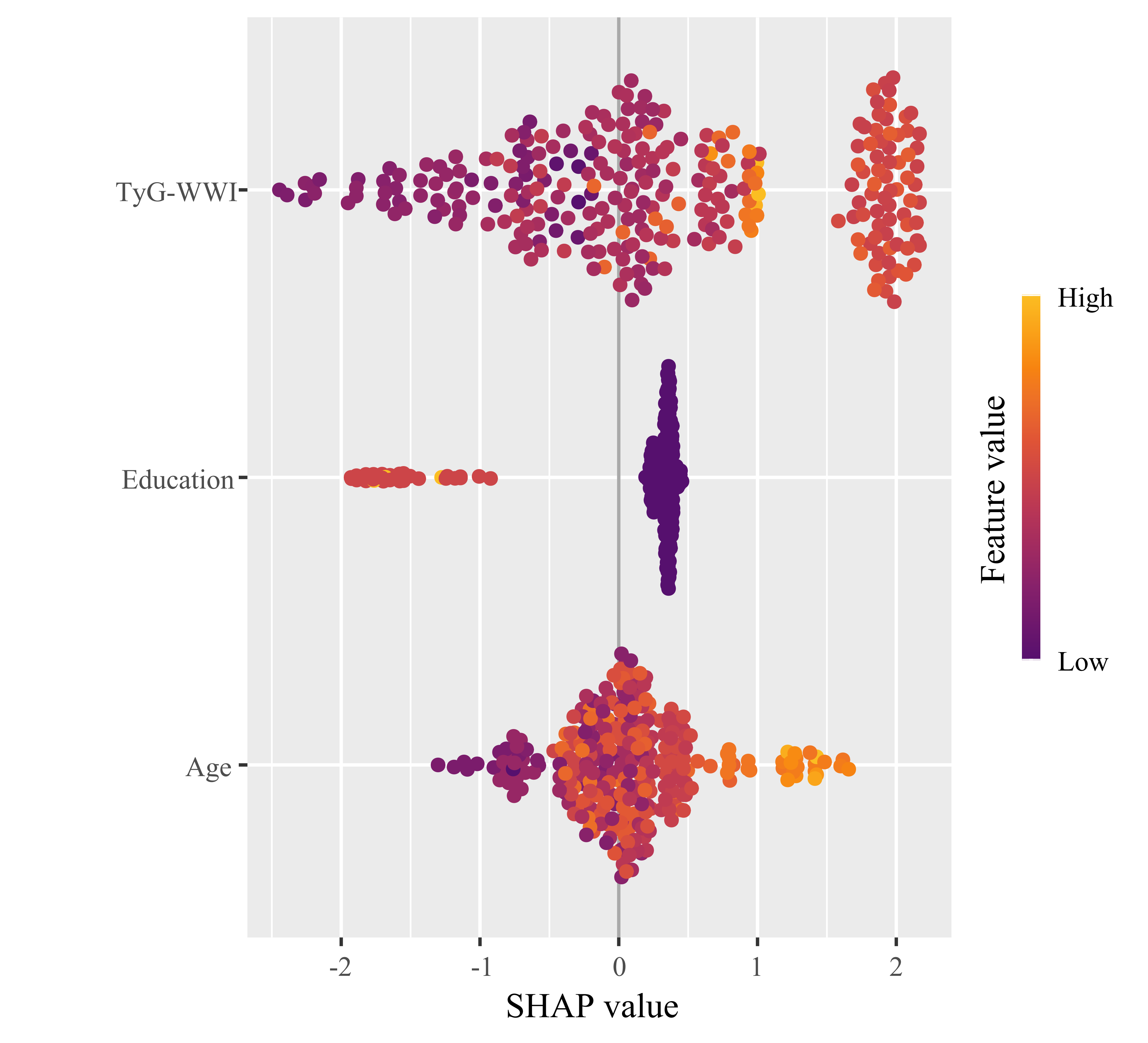


**Figure S4. The model’s interpretation.** Abbreviation: TyG-WWI, triglyceride-glucose index-waist-to-weight index.
