# Supplementary material for: Triglyceride–Glucose–Waist‐to‐Weight Index: Novel Biomarker for Mild Cognitive Impairment in Older People With Sarcopenic Obesity
Source: J Cachexia Sarcopenia Muscle. 2026 May 11;17(3):e70307. doi: 10.1002/jcsm.70307 (PMC13161463; doi:10.1002/jcsm.70307)
Supplement: Supplementary file 2 — Data S1: Supporting information. [file JCSM-17-e70307-s001.pdf]

KG Support Limited
www.kgsupport.com
info@kgsupport.com
Phone/Fax +852 3018 1216

REVIEW CERTIFICATION

DATE: MARCH 4, 2026

TO WHOM IT MAY CONCERN:

This memo certifies that the author of the below mentioned document had contracted our English language document review service for one (1) file, the particulars of which are listed below. The English review was conducted using a two-stage process, in which two editors reviewed the file, both of whom are native English speakers.

We therefore attest to the quality of the reviewed document.

KG Support Limited (Hong Kong)
www.kgsupport.com
info@kgsupport.com

Date of Review	DESCRIPTION (file name)
March 3, 2026	Triglyceride-glucose-waist-to-weight index: Novel biomarker for mild cognitive impairment in older people with sarcopenic obesity
